# Unraveling the anti-neuroinflammatory mechanisms of Cervus cucumis polypeptide injection in Alzheimer’s disease: insights from network pharmacology, molecular docking, molecular dynamics simulation, and experimental validation

**DOI:** 10.3389/fnagi.2026.1797302

**Published:** 2026-04-22

**Authors:** Xiaowei Fu, Jiahui Huang, Yan Liu, He Li, Yantao Zhang

**Affiliations:** 1 School of Pharmaceutical Sciences, Guangzhou University of Chinese Medicine, Guangzhou, China; 2 School of Pharmacy, Guangzhou Health Science College, Guangzhou, China

**Keywords:** Alzheimer’s disease, Cervus Cucumis Polypeptide Injection, molecular docking, molecular dynamics simulation, network pharmacology, neuroinflammation

## Abstract

**Objective:**

Alzheimer’s disease (AD) is a progressive neurodegenerative disorder with increasing global prevalence, in which neuroinflammation serves as a critical pathological driver exacerbating cognitive decline. While current therapies offer limited symptomatic relief, multi-target strategies are urgently needed. Cervus cucumis polypeptide injection (CCPI), a traditional Chinese medicine (TCM) formulation, has demonstrated anti-inflammatory properties; however, its mechanisms of action against AD remain unclear. This study aimed to elucidate the anti-AD potential mechanisms of CCPI using an integrated approach combining network pharmacology, molecular docking, molecular dynamics (MD) simulation, and experimental validation.

**Methods:**

Active components and corresponding targets of CCPI were retrieved from the TCMSP database, while AD-related targets were collected from Genecards, OMIM, and DrugBank. Potential therapeutic targets were identified by intersecting drug and disease targets, followed by protein–protein interaction (PPI) network construction, Gene Ontology (GO), and Kyoto Encyclopedia of Genes and Genomes (KEGG) enrichment analyses. Molecular docking and MD simulations were performed to evaluate interactions between potential active components and key targets. *In vitro* experiments were conducted on Aβ_25-35_-induced BV2 microglial cells to assess cell viability (CCK-8 assay), inflammatory cytokine levels (ELISA), and protein expression (Western blot) related to the neuroinflammation pathway and microglial polarization.

**Results:**

A total of 28 active components and 50 common targets of CCPI for AD treatment were identified. Linoleic acid (LA) was determined to be a potential active component, with IL-6 as the key target based on PPI network topology. Molecular docking and MD simulation confirmed a stable binding affinity between LA and IL-6. KEGG analysis revealed significant enrichment in the HIF-1 signaling pathway, particularly the IL-6/STAT3/VEGF signaling pathway. *In vitro*, CCPI treatment significantly enhanced cell viability and attenuated the pro-inflammatory response, as evidenced by reduced levels of IL-6, IL-1β, and TNF-α, decreased the expression of the pro-inflammatory marker iNOS. Concurrently, it elevated the expression of the anti-inflammatory/repair-associated marker CD206. Western blot analysis further verified that CCPI suppressed IL-6/STAT3 activation while upregulating VEGF expression. Additionally, LA alone significantly reduced IL-6 levels and STAT3 phosphorylation, decreased the expression of iNOS, and increased the expression of CD206, with therapeutic efficacy comparable to CCPI.

**Conclusion:**

CCPI exerts neuroprotective effects in AD models by regulating the IL-6/STAT3/VEGF pathway, downregulating the expression of the inflammation-related iNOS protein, upregulating the expression of the CD206 protein associated with anti-inflammatory and reparative functions, remodeling the functional state of microglia, inhibiting their pro-inflammatory responses, and enhancing their reparative functions. Its potential active component, LA, likely mediates this effect by stably binding to and inhibiting IL-6, thus suppressing the downstream STAT3 phosphorylation that drives inflammatory activation.

## 1 Introduction

Alzheimer’s Disease (AD) is a progressive, irreversible neurodegenerative disorder, characterized by memory degeneration and other cognitive disturbances, which poses a severe threat to the patients’ quality of life (Frisoni et al., 2010; Hamelin et al., 2016). With the aging of the global population, the number of AD patients is projected to reach 152 million by 2050, making it a critical public health challenge (Scheltens et al., 2021). The primary pathological hallmarks of AD include the accumulation of amyloid-β (Aβ) plaques and neurofibrillary tangles composed of hyperphosphorylated of intraneuronal tau, causing neuronal and synaptic loss, ultimately leading to cognitive impairment (Long and Holtzman, 2019; Braak and Braak, 1991; Hardy and Selkoe, 2002). Aβ deposition triggers a persistent neuroinflammatory response by activating microglia; this inflammatory response not only drives the abnormal phosphorylation and fibrillary aggregation of tau protein but also induces astrocytes to transform into a pro-inflammatory phenotype, forming a positive feedback loop that further promotes Aβ accumulation and accelerates neuronal functional degeneration and structural damage (Hickman et al., 2008; Thakur et al., 2023; Paudel et al., 2020; Singh, 2022). Microglia, as the primary innate immune phagocytes in the central nervous system (CNS), initiate a series of dynamic responses when stimulated by Aβ pathological deposition; these responses include local proliferation, physical adhesion to Aβ plaques, and significant transcriptional reprogramming, ultimately leading to a characteristic activated state defined as disease-associated microglia (DAM) (Prinz et al., 2021; Thion et al., 2018; Keren-Shaul et al., 2017; Friedman et al., 2018; Krasemann et al., 2017; Marsh et al., 2022). More importantly, neuroinflammation is recognized as an independent factor in the pathogenesis of AD since it exacerbate neuronal damage and even leading to neuronal death by releasing pro-inflammatory cytokines, such as interleukin-1β (IL-1β) and tumor necrosis factor α (TNF-α) (Dai et al., 2018; Schöll et al., 2012; Berger et al., 2007; Edison et al., 2008; Xie et al., 2020). Neuronal death may disrupt the homeostatic phenotype of microglia (Krasemann et al., 2017) and may also lead to the release of damage associated molecular patterns (DAMPs), further exacerbating inflammation and creating a vicious cycle (Sarhan et al., 2018). Current clinical treatments for AD, including cholinesterase inhibitors like galantamine, primarily offer symptomatic relief but fail to halt the disease’s progression due to their single-target nature and associated side effects (Wang et al., 2024; Konecny et al., 2020; Luo Z. et al., 2020). Given the complex nature of AD, targeting a single pathological factor through pharmacotherapy may prove insufficient, which highlights the necessity for the development of multi-target therapies that can effectively modulate complex pathways like neuroinflammation (Liu et al., 2018; Liu et al., 2024).

Traditional Chinese Medicine (TCM), known for its comprehensive and multi-target approaches with fewer side effects, has been suggested to be beneficial in treating complex neurodegenerative diseases, emerging as a promising strategy against AD (Lv et al., 2024). In TCM, AD belongs to the categories of “dementia,” “madness,” and “amnesia,” with its pathogenesis attributed to kidney essence deficiency, marrow depletion, and phlegm-turbidity obstruction, leading to the loss of mental clarity. Therefore, therapeutic strategies emphasize nourishing the kidney, replenishing essence, and resolving phlegm to restore cognitive function (Pei et al., 2020).

In classical Chinese medicine texts, the limbs of the sika deer (*Cervus nippon* Temminck) are known as deer bone, which enters the kidney meridian. The Thousand Gold Prescriptions records “deer bone decoction,” which warms kidney yang, strengthens tendons and bones, and nourishes deficiency and emaciation (Sun et al., 1955). Meanwhile, the dried mature seeds of the melon plant (*Cucumis melo* L.), known as muskmelon seeds, are documented in the Chinese Materia Medica as possessing properties that generate dampness and heat, yet also possess effects such as relieving cough and resolving phlegm, tonifying the kidneys and replenishing essence (State Administration of Traditional Chinese Medicine “Chinese Materia Medica” Editorial Board, 1999).

In modern times, a sterile aqueous solution derived from separate extracts of sika deer bone and muskmelon seeds forms the basis of a new compound traditional Chinese medicine preparation, Cervus cucumis polypeptide injection (CCPI, Songmeile®), which exhibits anti-inflammatory and bone-strengthening effects (Guo et al., 2008; Zhang et al., 2017). Currently, CCPI is applied in China for treating various orthopedic conditions, including rheumatoid arthritis, with notable clinical efficacy (Zhang et al., 2025). Furthermore, prior pharmacological studies indicate that CCPI can suppress inflammatory exudation (Qi et al., 2020; Wei et al., 2016; Xu et al., 2013). Among them, the bone-induced polypeptide biological factors present in deer melon polypeptide can transform into growth factor-β (TGF-β), which effectively inhibits the inflammatory response and reduces the concentration of TNF-α in the serum (Xiao et al., 2018; Bian et al., 2018). The mature seeds of the gourd melon in the deer melon polypeptide can reduce the capillary permeability in the local tissue and the exudation of inflammatory factors (Han et al., 2019).

Although CCPI was not developed as a therapeutic agent for AD, the convergence of AD’s neuroinflammatory pathogenesis and the known anti-inflammatory effects of CCPI suggests its considerable potential for AD treatment. However, systematic research on the direct effects and molecular mechanisms of CCPI in combating neuroinflammation in AD remains lacking. To this end, the present study is designed to validate the anti-AD efficacy of CCPI and to decipher its multi-target mechanisms through an integrated approach. Network pharmacology, as a cost-effective alternative to traditional drug discovery, particularly excels in elucidating the complex relationships between multi-component traditional Chinese medicines and their targets (Tian et al., 2021; Lv et al., 2024). Leveraging this approach, high-throughput bioinformatics techniques can construct “drug-compound-target-disease” network models (Tang and Aittokallio, 2014). Meanwhile, molecular docking analysis and molecular dynamics (MD) simulation further validate the binding patterns between the main components of TCM and specific targets. In this study, we utilized network pharmacology methods and molecular docking techniques to predict the active components and mechanisms of CCPI in alleviating AD, validated the findings through experimental approaches to establish a foundation for future research on advancing the development of CCPI for treating AD as a new indication.

## 2 Materials and methods

### 2.1 Screening for potential active ingredients and targets of CCPI

The active ingredients of CCPI were searched through the Traditional Chinese Medicine Systems Pharmacology Database and Analysis Platform (TCMSP).^1^ The active ingredients of CCPI and their corresponding targets were screened based on the following criteria: oral bioavailability (OB) ≥ 30%, drug-likeness (DL) ≥ 0.18, and blood–brain barrier (BBB) permeability ≥ − 0.30 (Cheung et al., 2024; Li et al., 2020). The Uniprot database^2^ was utilized to search for the gene name of the target protein, with the “Organisms” parameter set to “*Homo sapiens*.” The results from these databases were merged and duplicate targets were removed to obtain the targets of CCPI.

### 2.2 Screening of AD related targets and Venn diagram analysis

Using the search term “Alzheimer’s disease” and setting the species to *Homo sapiens*, the DrugBank database,^3^ the OMIM database,^4^ and the GeneCards database^5^ were utilized to acquire AD-related targets. Active targets retrieved from the Genecards database were collected with a relevance score of ≥10 as a screening criterion (Shi et al., 2023). The targets of CCPI and the disease targets of AD were entered into the VENNY 2.1.0^6^ to obtain the intersecting targets and draw a Venn diagram.

### 2.3 Construction of the protein–protein interaction (PPI) network

The Cytoscape 3.7.1 software was used to construct and analyze the “ingredient-target” network diagram of CCPI. The intersection targets were imported into STRING^7^ with the PPI network constructed. The topological properties of the network were analyzed by the network analyzer in Cytoscape 3.7.1 software, and the targets were ranked according to the node degree value.

### 2.4 Enrichment analysis of the intersected genes

The obtained core targets were imported into the DAVID^8^ for Gene Ontology (GO) enrichment analysis and Kyoto Encyclopedia of Genes and Genomes (KEGG) pathway enrichment analysis, with *p* < 0.05 were considered significantly enriched. The results of pathway enrichment analysis were plotted by the Bioinformatics^9^ online platform.

### 2.5 Molecular docking

The screened key genes served as the key targets for receptors, and the information on receptor structures was obtained from PDB.^10^ The 2D structures of active ingredients were obtained from TSCMP databases, and the optimized ligands were used as starting points for docking. The energy minimization was carried out with the Minimize mode of Chem3D software. Subsequently, the file was converted to Mol2 format, and then the Mol2 format was transformed into pdbqt format using AutoDock Vina 1.1.2. The conformation and the lowest binding energy of ligand-receptor interactions was obtained after the docking search was completed. Finally, the receptor-ligand binding image was visualized by PyMOL software.

### 2.6 MD simulation

MD simulation of the complex was performed using the GROMACS 2020.3 software package with the CHARM 36 all-atom force field and TIP3P water model, with a simulation time of 50,000 ps. The post-docking small-molecule ligand was hydrogenated using Avogadro 1.2.0 and uploaded to the CGenFF website to construct a topology file, which was subsequently combined with the protein topology file to yield the complete system topology file. The complex was placed within a dodecahedral box of simple point charge (SPC) water, with a 1 nm distance maintained between the complex and the box edges. Sodium or chloride ions were added to the system to neutralize the charges. After energy minimization, the system was equilibrated at 310 K and 1 bar, followed by a 20 ns simulation. Finally, the trajectory files were analyzed to obtain system properties including root mean square deviation (RMSD), root mean square fluctuation (RMSF), protein radius of gyration (Rg), solvent accessible surface area (SASA) and the number of hydrogen bonds, which were visualized using Origin Lab 2018b.

The visualization of the free energy landscape (FEL) was constructed by calculating Gibbs free energy as a function of RMSD and Rg. The Gibbs free energy was mapped onto a two-dimensional space to visualize the free energy surface. Subsequently, the free energy surface was presented simultaneously as a three-dimensional surface plot and a two-dimensional contour plot. The three-dimensional plot employed color coding: red indicates low-energy states, while blue represented high-energy regions. The two-dimensional contour plot used a similar color scheme, where red areas corresponded to low free energy distributions, and blue areas corresponded to high free energy states.

### 2.7 Experimental validation

#### 2.7.1 Drug preparation and cell culture

CCPI consists of *Cervus nippon* Temminck extract and *Cucumis melo* L. extract. LA was detected in CCPI using reversed-phase liquid chromatography-hydrophilic interaction liquid chromatography–tandem mass spectrometry (RPLC-HILIC-MS/MS) (Liu et al., 2020), but no quantitative analysis was performed. However, Linoleic acid (LA) is naturally present in *Cucumis melo* L., and the LA content in *Cucumis melo* L. obtained by supercritical CO_2_ extraction reached as high as 64.293%, while that in *Cucumis melo* L. produced by traditional Soxhlet extraction also reached 56.995% (KaHar, 2014).

The peptide Aβ_25-35_ (CAS No. 131602-53-4, MCE, United States) was completely dissolved in three-distilled water, then stored at −20 °C and incubated at 37 °C for 7–14 d before being used to complete the aging treatment. CCPI (Heilongjiang Dilong Pharmaceutical Co., Ltd., Harbin, China) conforms to the standard (No. WS1-XG-002-2002-2005) set by the Chinese Pharmacopoeia Commission. It is diluted to a final concentration in Phosphate Buffer Saline (PBS) buffer (CAS No. 12111-21-6, Macklin, China) before use. To prepare a 20 mM LA (cell culture grade standard, purity > 99%, CAS No. 60-33-3, Sigma, United States) stock solution, combine 345 μL of LA with 50 mL of 0.1 M NaOH solution under constant stirring. The stock solution was filtered through a 0.22 μm membrane filter and subsequently stored at −20 °C.

BV2 microglial cells (Nantong Feiyu Biotechnology Co., LTD., China) were cultured in high glucose Dulbecco’s Modified Eagle Medium (DMEM, CAS No. C11995500BT, Thermo Fisher, United States) medium containing 10% fetal bovine serum (FBS, CAS No. BMC1020, Abbkine, United States) and 1% penicillin–streptomycin (PS, CAS No. V900929, Sigma, United States) in a humidified incubator at 37 °C and 5% CO_2_.

#### 2.7.2 Cell viability assay

The cell counting kit-8 (CCK-8, Beyotime, Shanghai, China) assay was used to determine the viability of BV2 microglial cells. BV2 microglial cells are seeded in 96-well microtiter plates at a density of approximately 1 × 10^4^ cells per well. To define the optimal concentration and exposure window of CCPI, the cells were incubated in fresh complete culture medium containing CCPI (0, 15, 30, 60, 120, and 240 μg/mL) and LA (0, 10, 20, 40, 80, and 160 μM) for 24 h or treated with 120 μg/mL CCPI for 8, 16, 24 and 48 h. The Optical Density (OD) was measured at 450 nm using a Multifunctional enzyme-linked immunosorbent assay reader (BioTek Synergy H1, Agilent) in accordance with the manufacturer’s instructions. Similarly, the viability of BV2 microglial cells induced by different concentrations of CCPI (15, 60, and 120 μg/mL) or LA (10, 40, and 80 μM) in combination with 20 μM Aβ_25-35_ (AD model cells) was measured via the CCK-8 assay. Cell viability (%) = [(OD of experimental group − OD of zero adjustment group)/(OD of control group − OD of zero adjustment group)] × 100%.

#### 2.7.3 Drug treatment

##### 2.7.3.1 Treatment with CCPI

BV2 microglial cells were divided into five groups: the Control group (BV2 microglial cells without any treatment), the AD model group [BV2 microglial cells treated with Aβ_25-35_ at a concentration of 20 μM (Cheng et al., 2025) for 24 h], and the CCPI group (BV2 microglial cells pre-treated with Aβ_25-35_ and then post-treated with CCPI at concentrations of 15, 60, and 120 μg/mL for 24 h).

##### 2.7.3.2 Treatment with LA

BV2 microglial cells were divided into five groups: the Control group, the AD model group, and the LA group (10, 40, and 80 μM).

##### 2.7.3.3 Comparison of CCPI and LA treatment

BV2 microglial cells were divided into four groups: the Control group, the AD model group, the CCPI group (120 μg/mL), and the LA group (80 μM).

#### 2.7.4 Enzyme-linked immunosorbent assay (ELISA)

After treatment, the culture media of BV2 microglial cells from experimental groups were collected (*n* = 6 each). The ELISA kit was then used according to the manufacturer’s instructions to measure the levels of IL-6, IL-1β, and TNF-α. Absorbance was measured at a wavelength of 450 nm using a Multifunctional enzyme-linked immunosorbent assay reader.

#### 2.7.5 Western blot

BV2 microglial cells were incubated in Radio-Immunoprecipitation Assay (RIPA) buffer (CAS No. R0278, Sigma, United States) with protease inhibitors to prepare whole-cell lysates. The protein concentration of the lysates was detected by applying BCA kit (Art. No. P0010, Beyotime, China). Equivalent amounts of protein for each sample were denatured by boiling at 95 °C for 5 min and separately using SDS-polyacrylamide (SDS-PAGE) gel (CAS No. ZS306-2, ZOMANBIO, China) electrophoresis, which were then transferred to polyvinylidene difluoride (PVDF) membranes (CAS No. 24937-79-9, Sigma, United States). After being blocked in 5% skim milk for 2 h at room temperature, the membranes were incubated overnight with the following primary antibodies: inducible nitric oxide synthase (iNOS) mouse antibody (CAS No. IC259554, Abmart, China, 1:1,000), CD206 mouse antibody (CAS No. ZY-5843R, Abmart, China, 1:1,000), IL-6 mouse antibody (CAS No. 66146-2, Abmart, China, 1:1,000), STAT3 mouse antibody (CAS No. YA056, Abmart, China, 1:1,000), STAT3 phosphorylation mouse antibody (CAS No. 05-485, Sigma, United States, 1:1,000), VEGF rabbit antibody (CAS No. AF1309, Abmart, China, 1:1,000) and GAPDH rabbit antibody (CAS No. 10494-1-AP, Abmart, China, 1:1,000). Then the PVDF membranes were incubated with Goat anti-rabbit HRP secondary antibody (CAS No. 31460, Invitrogen, United States, 1:5,000) and Goat anti-mouse secondary antibody (CAS No. SA00001-5, Proteintech, United States, 1:5,000) for 1 h at 37 °C. The membranes were examined using a gel imaging device (ChemiDoc MP, United States), and the gray scale value of blots were analyzed using ImageJ software.

#### 2.7.6 Statistical analysis

**Figure 1:**
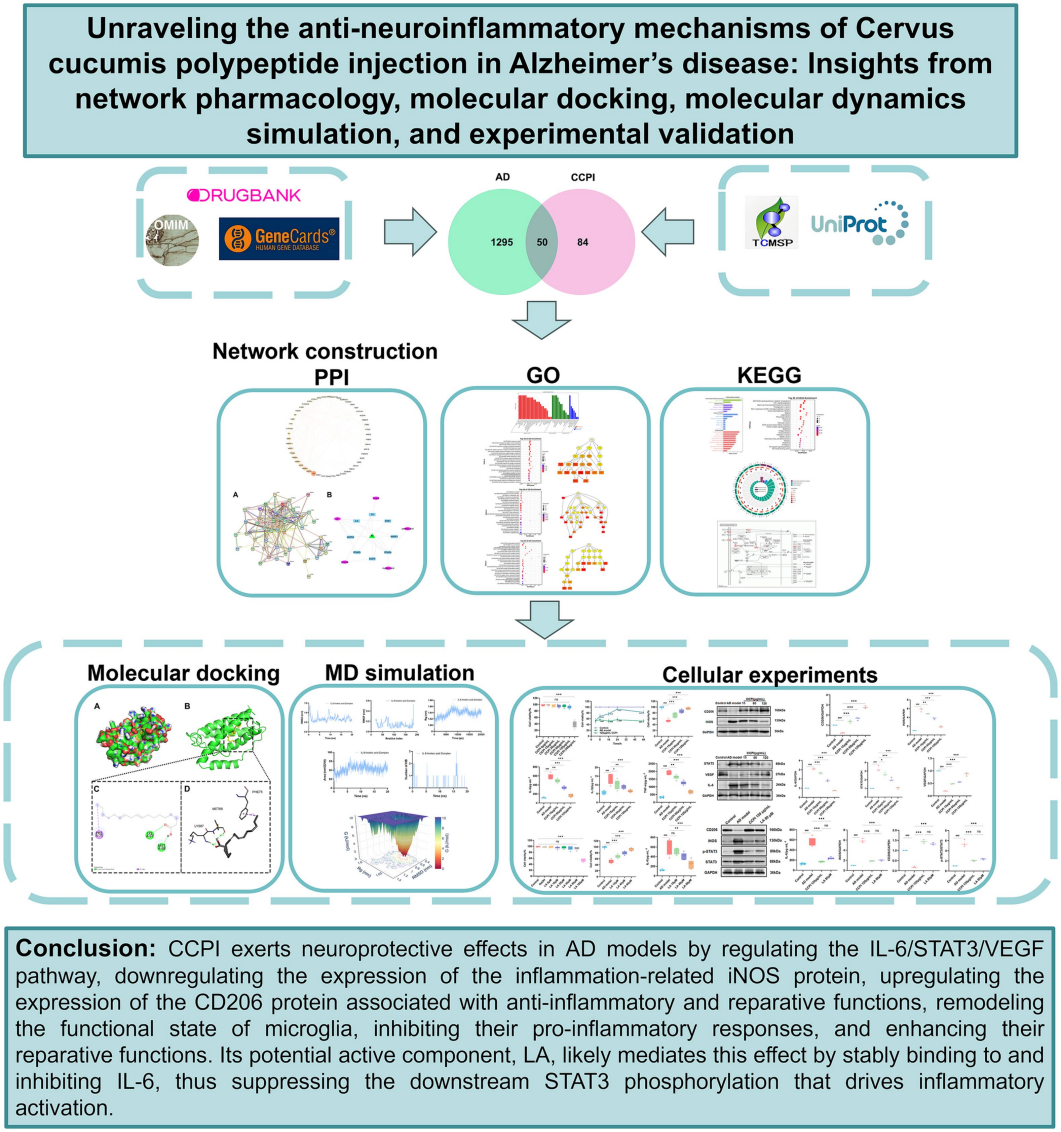
Flowchart showing the network pharmacological and experimental studies for the investigation of the anti-neuroinflammation effects of CCPI in AD.

Statistical analysis and figure generation were performed by GraphPad Prism software version 10.0 (LaJolla, CA, United States). All data were presented as the mean ± standard deviation (SD). Data meeting normal distribution criteria were evaluated using one-way analysis of variance (ANOVA) followed by Student–Newman–Keuls post-hoc test. Data not meeting normal distribution criteria were analyzed using the Kruskal-Wallis test. A value of *p* < 0.05 was considered statistically significant (Figure 1).

## 3 Results

### 3.1 Identification of predicted bioactive compounds of CCPI and AD-related targets

After searching the TCMSP database, 28 predicted bioactive compounds and 134 potential therapeutic targets of CCPI were obtained (Supplementary Table 1). Subsequent the OB, DL, and BBB permeability of these components was predicted using Discovery Studio software, indicating that compound LA exhibited the highest BBB permeability score (Table 1).

**Table 1:** The OB, DL, and BBB permeability of the active ingredients in CCPI.

No.	Active components	OB/%	DL	BBB
1	Linoleic acid (LA)	32.0	0.32	1.226
2	Glucose	45.3	0.45	−0.085
3	Guanosine	42.1	0.41	−0.073
4	Shikimic acid	38.9	0.36	−0.052
5	Chloroacetic acid (CLR)	48.5	0.47	−0.034

**Figure 2:**
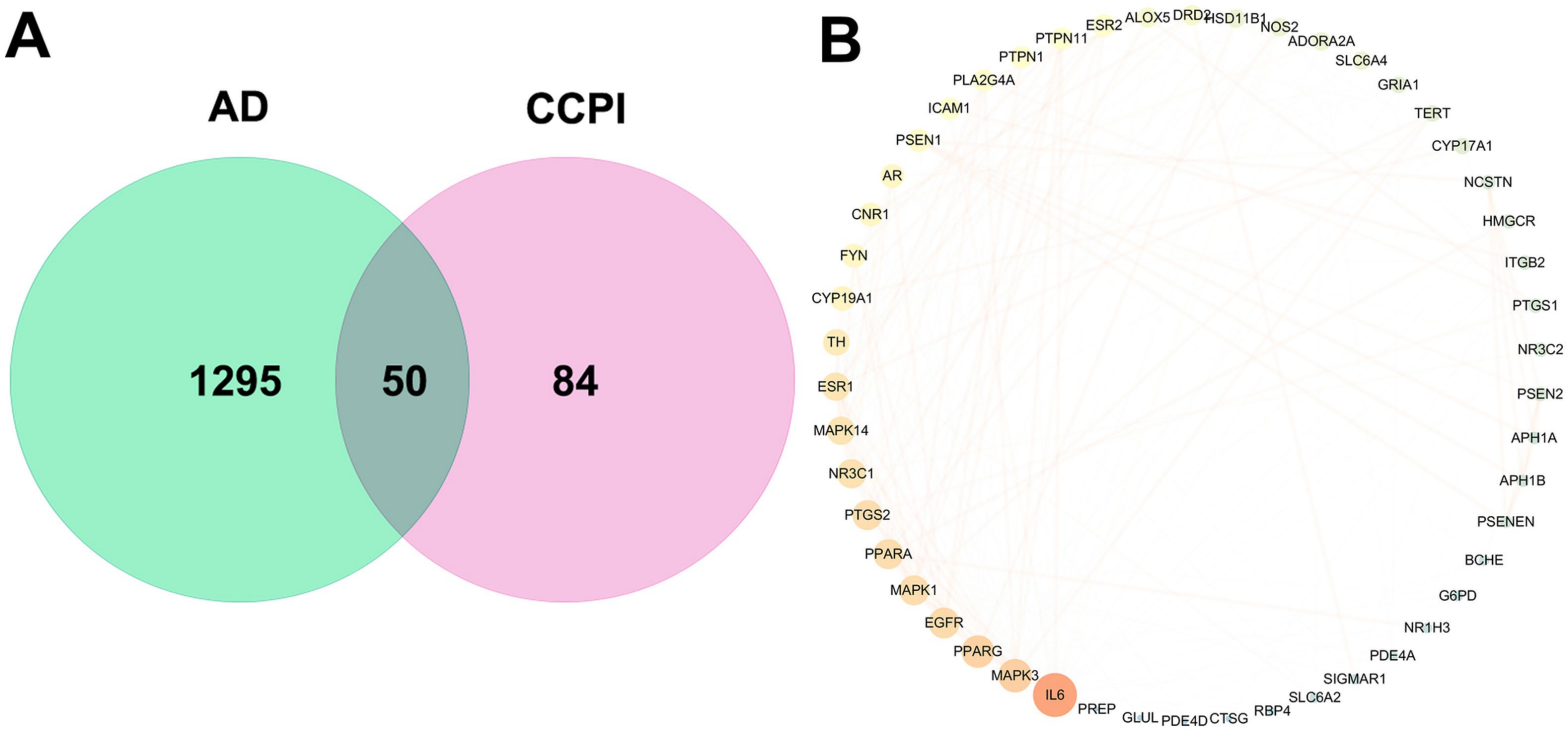
Analysis of key targets for CCPI in treating AD. **(A)** Venn diagram of potential therapeutic targets for CCPI in AD. **(B)** Core network diagram of CCPI targets (nodes represent target proteins, with node size proportional to the degree values; edges denote protein–protein interactions).

After retrieval from the DrugBank, OMIM, and GeneCards databases and removal of redundant entries, 1,795 AD-related targets were identified. The screening criteria were set with a “Closeness” threshold of 0.070, a “Betweenness” threshold of 174.804, and a “Degree” threshold of 6.187. The obtained protein-interaction data were imported into Cytoscape software to construct network diagrams, and the final identification of 50 disease targets for AD was achieved through Venn diagram (Figures 2A,B; Supplementary Table 2).

### 3.2 Construction and analysis of PPI network

**Figure 3:**
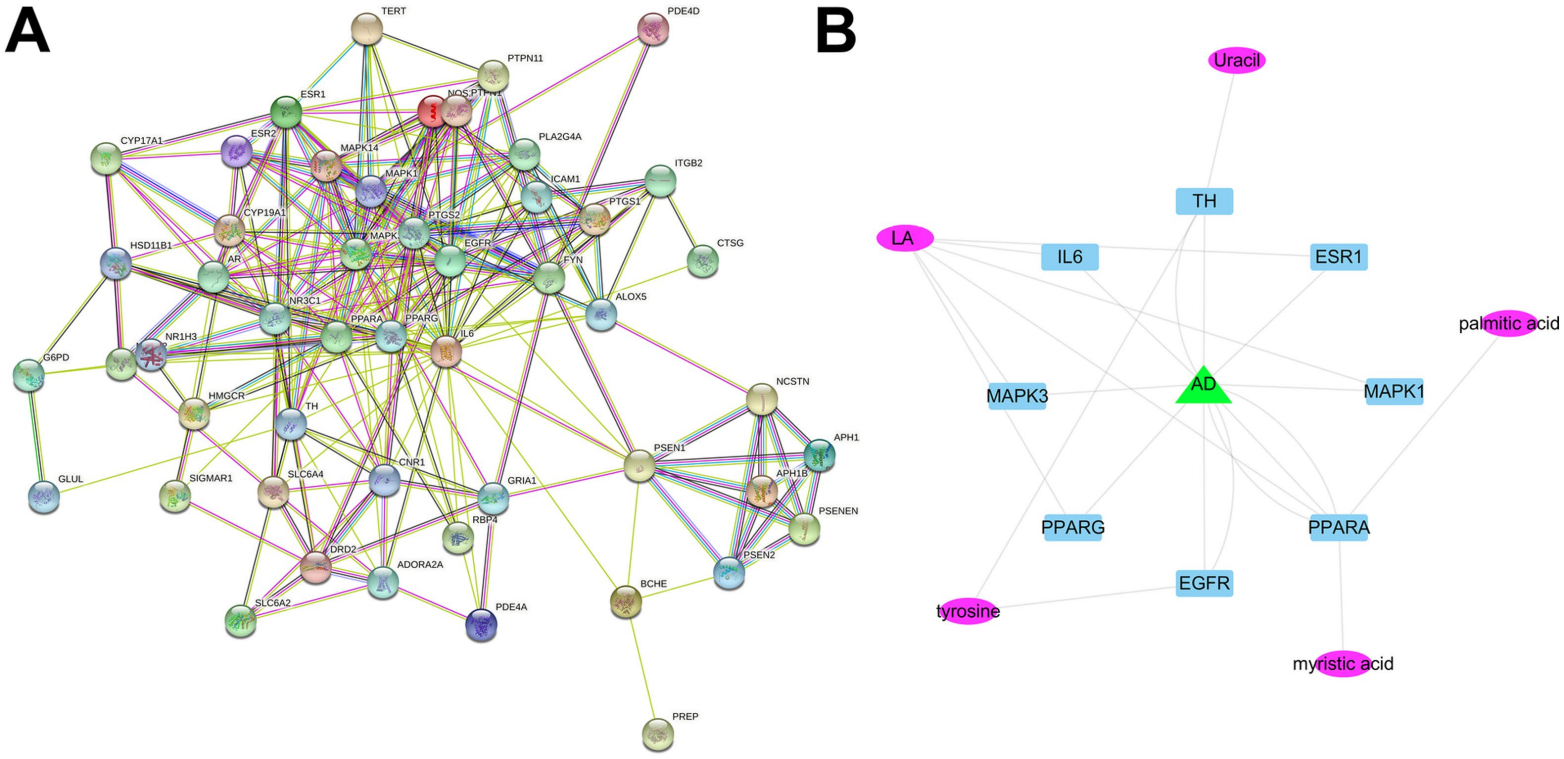
CCPI active components-AD targets PPI network. **(A)** PPI network of potential CCPI active targets. **(B)** Core PPI network of CCPI active components and AD targets.

To further investigate the mechanism of CCPI in treating AD, we utilized 50 common targets and input them into the STRING database for PPI network analysis. The resulting PPI network comprised 50 target nodes connected by 245 edges (Figure 3A). Targets with degree values ≥15 were identified as the core therapeutic targets of CCPI for AD (Figure 3B). IL-6 ranked first with a degree value of 35 among the network of these 50 overlapping targets (Table 2; Supplementary Table 3), exhibiting connectivity nearly 1.5 times higher than that of MAPK3 (degree value = 23), which ranked second. This indicates that in the network pharmacology-predicted model, IL-6 interacts proteomically with the vast majority of other key AD-related targets.

**Table 2:** Targets with degree values ≥15.

No.	Targets	Degree values
1	IL6	35
2	MAPK3	23
3	PPARG	22
4	EGFR	20
5	MAPK1	19
6	PPARA	19
7	PTGS2	19
8	NR3C1	18
9	MAPK14	17
10	ESR1	17
11	TH	15

After identifying highly interactive proteins (degree value ≥ 15) through network topology analysis, we integrated the known pathophysiological background of AD to conduct prioritized screening for key potential targets such as IL-6, which has been widely reported in key AD processes including neuroinflammation and Aβ cascade reactions.

### 3.3 Go enrichment analysis

**Figure 4:**
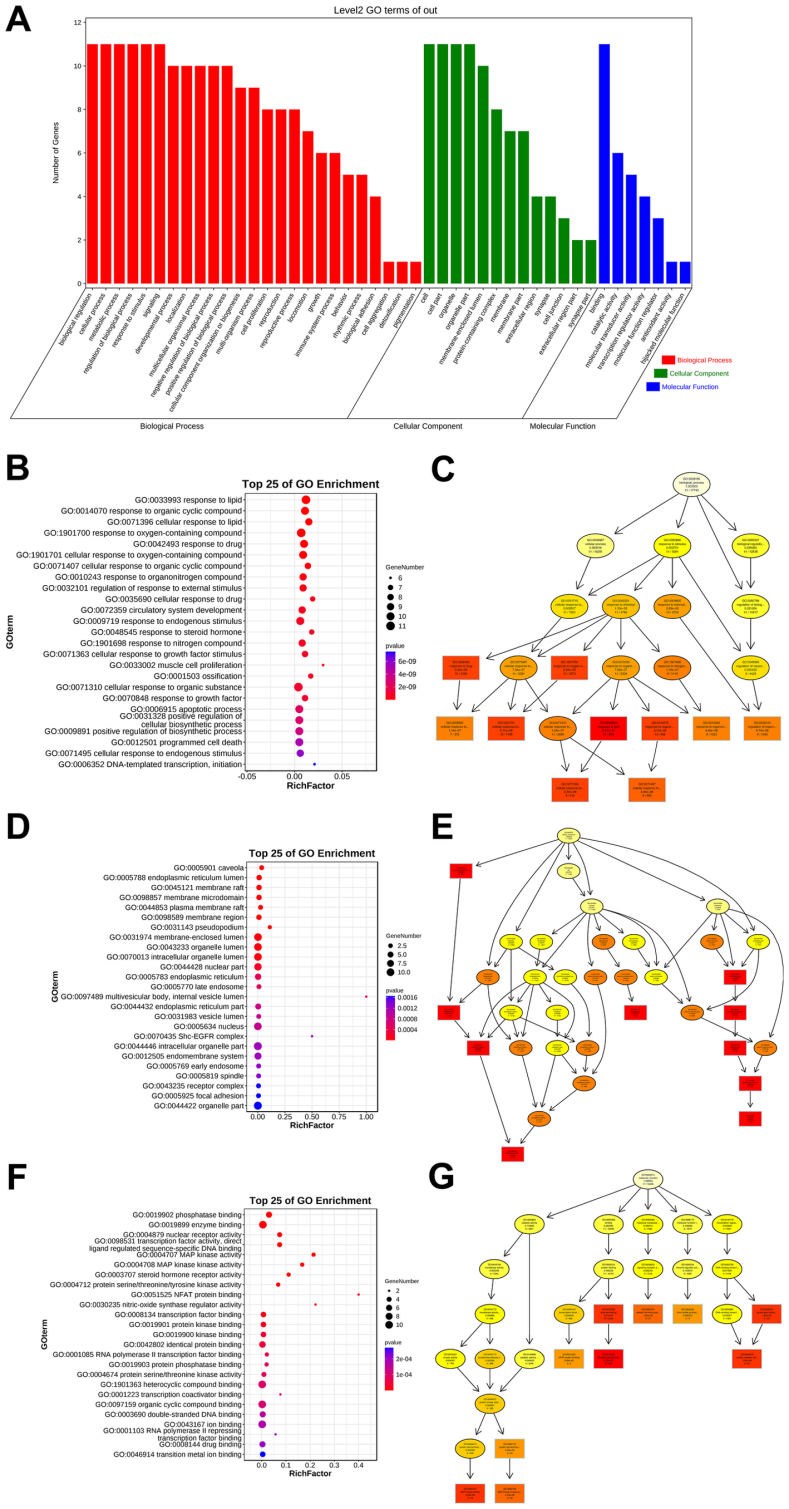
GO enrichment analysis. **(A)** Enriched GO terms and GO number. **(B,C)** The top 25 terms of BP and GO ID number. **(D,E)** The top 25 terms of CC and GO ID number. **(F,G)** The top 25 terms of MF and GO ID number.

To elucidate the therapeutic mechanisms of CCPI, functional enrichment analysis was performed using DAVID pathway analysis on core targets overlapping with AD pathogenesis and CCPI-active components. GO analysis revealed 2,631 significant terms (Supplementary Table 4), comprising 2,246 GO biological process (BP), 194 GO cellular component (CC), and 191 GO molecular function (MF) (Figure 4A). Significantly enriched BP terms primarily included response to lipid, response to oxygen-containing compound, and cellular response to organic substance (Figures 4B,C). CC terms included membrane-enclosed lumen, organelle lumen, and intracellular organelle lumen (Figures 4D,E). MF terms encompassed enzyme binding, phosphatase binding, and identical protein binding (Figures 4F,G). Take together, our results indicate that the effect of CCPI on AD is primarily associated with these biological processes, cellular component, and molecular function.

### 3.4 KEGG pathway enrichment analysis

**Figure 5:**
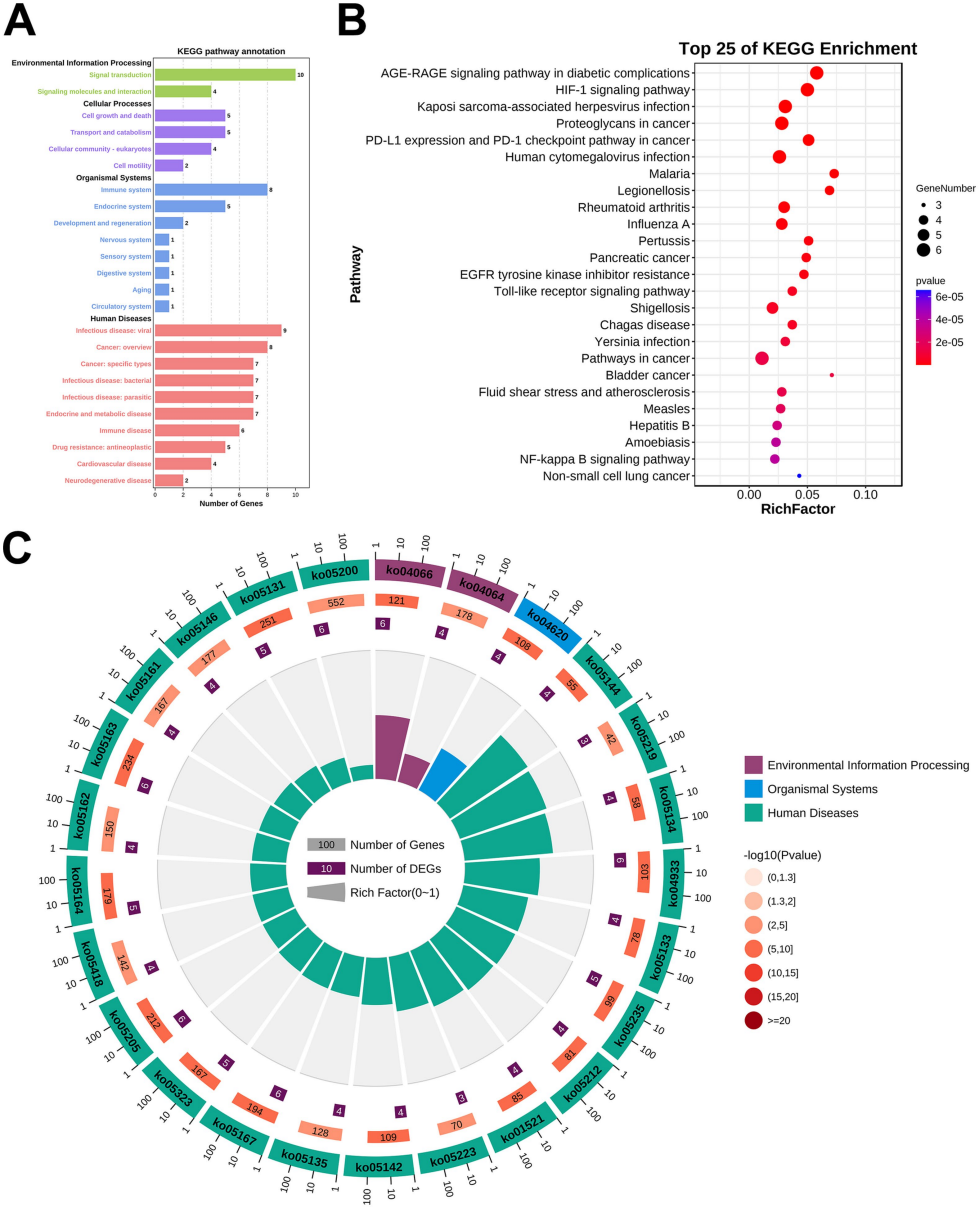
KEGG pathway enrichment analysis. **(A)** Enriched KEGG terms. **(B)** The top 25 KEGG signal pathways. **(C)** KEGG ID number.

KEGG pathway enrichment analysis pinpointed 140 signaling cascades (Supplementary Table 5), with the top 25 pathways ranked by −log10(p) value were used to construct a bubble map, notably including the HIF-1 signaling pathway, Toll-like receptor signaling pathway, and NF-kappa B signaling pathway (Figures 5A–C).

**Figure 6:**
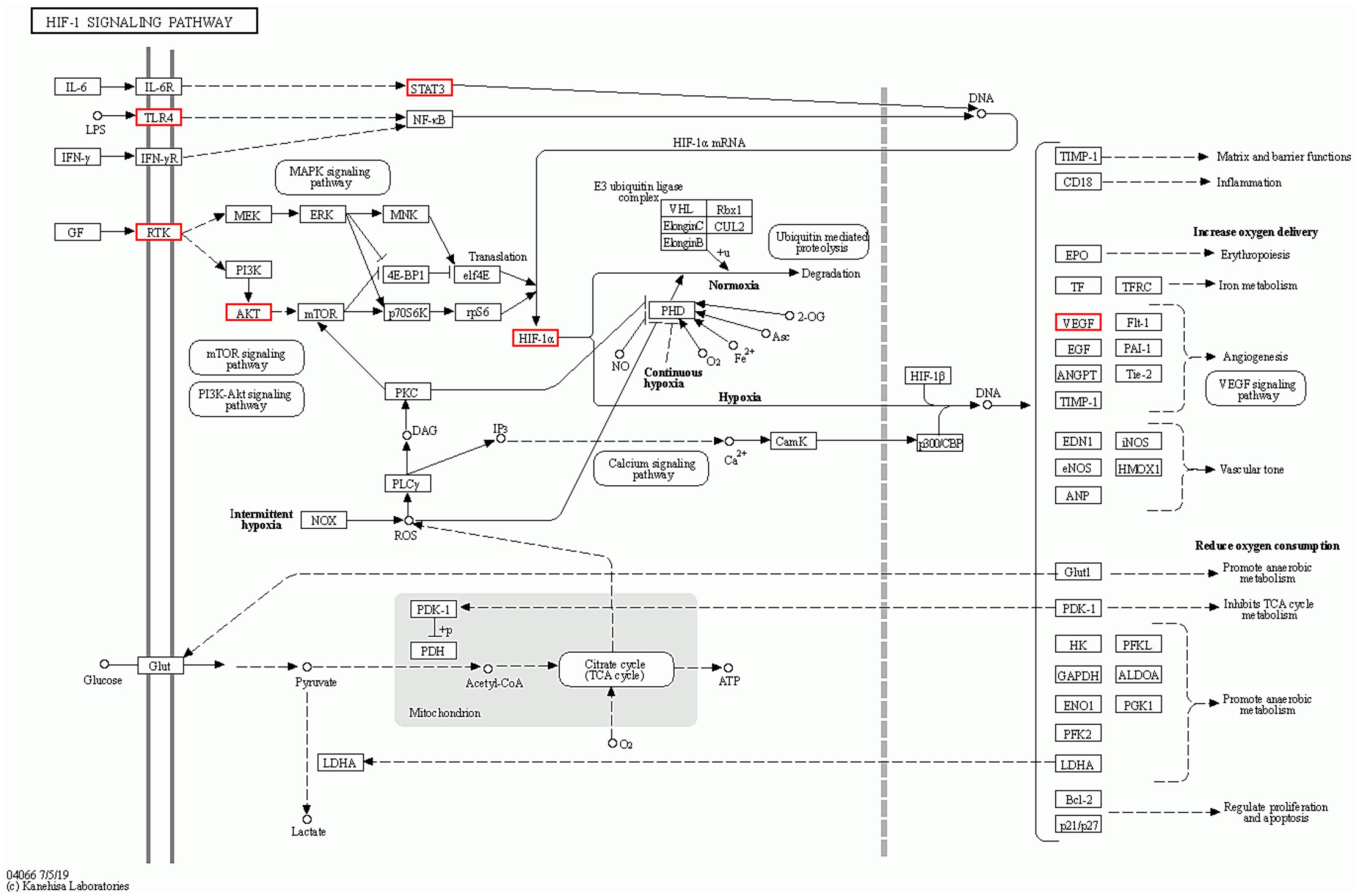
Diagrams of the HIF-1 pathway. The red mark represents the enrichment of the target of CCPI in the HIF-1 pathway.

Given that the HIF-1 signaling pathway exhibited the highest enrichment of genes, it will be designated as the primary research pathway for subsequent investigations. As depicted in Figure 6, the HIF-1 signaling pathway was primarily associated with targets such as IL-6, STAT3, and VEGF.

### 3.5 Molecular docking

**Figure 7:**
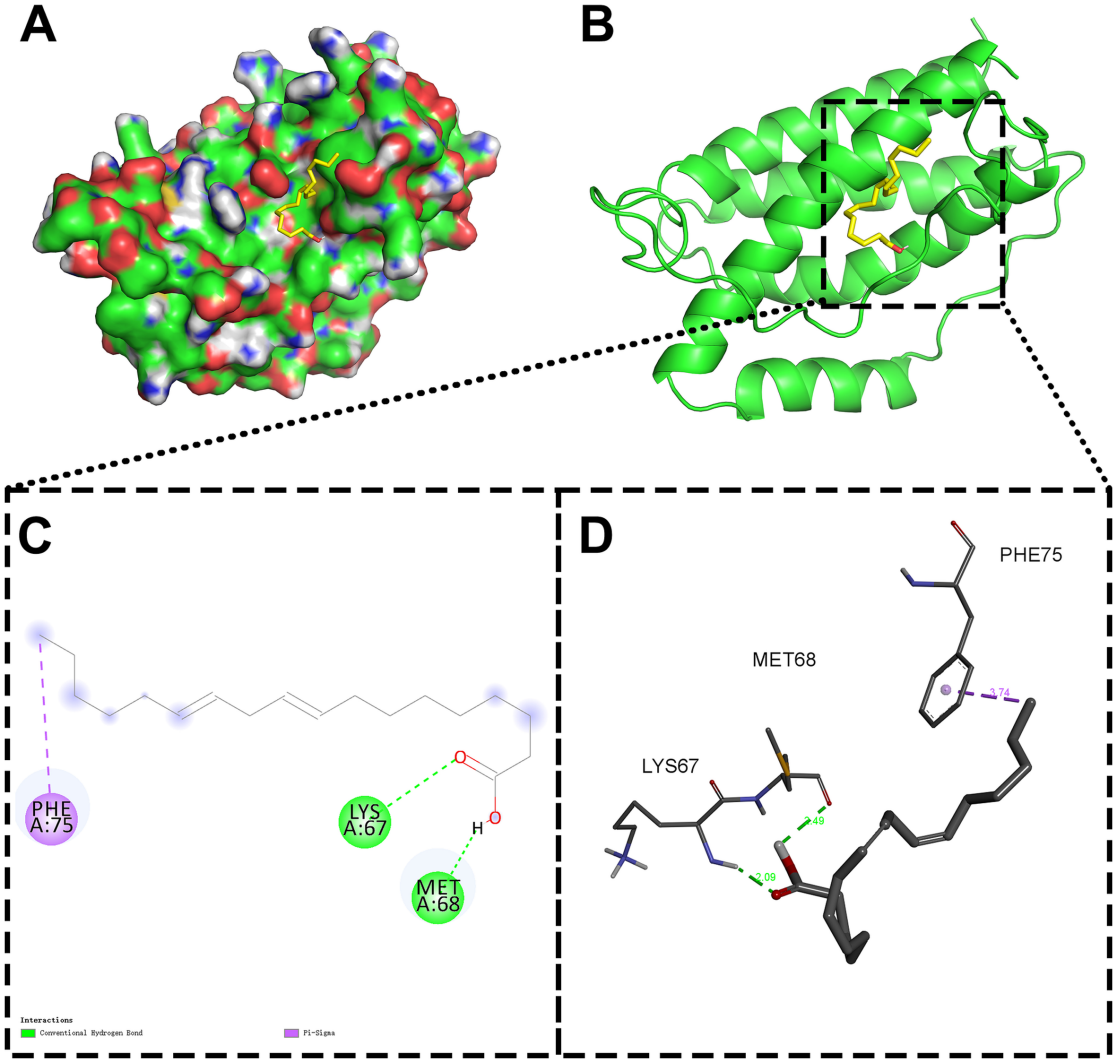
Molecular docking analysis of LA with IL-6 in both 3D and 2D frameworks. **(A)** Surface-form molecular docking. **(B)** Ribbon-form molecular docking. **(C)** 2D intermolecular interactions. **(D)** 3D intermolecular interactions.

To biophysically validate network pharmacology predictions, molecular docking was performed between the potential active component (LA) in the CCPI network and the target (IL-6) with the highest degree values. It has been established that IL-6 possesses significant binding capacity with LA when the docking energy is below −5.0 kcal/mol. Structural analysis revealed that LA forms two critical hydrogen bonds with IL-6: one with LYS67 (A) and another with MET68 (A) (Figure 7).

### 3.6 MD simulation

Following molecular docking, MD simulation was conducted on the ligand-protein complex system to assess its dynamic stability. The simulation evaluated parameters for the best-fit molecules, including RMSD, RMSF, Rg, SASA, hydrogen bond count analysis and FEL.

**Figure 8:**
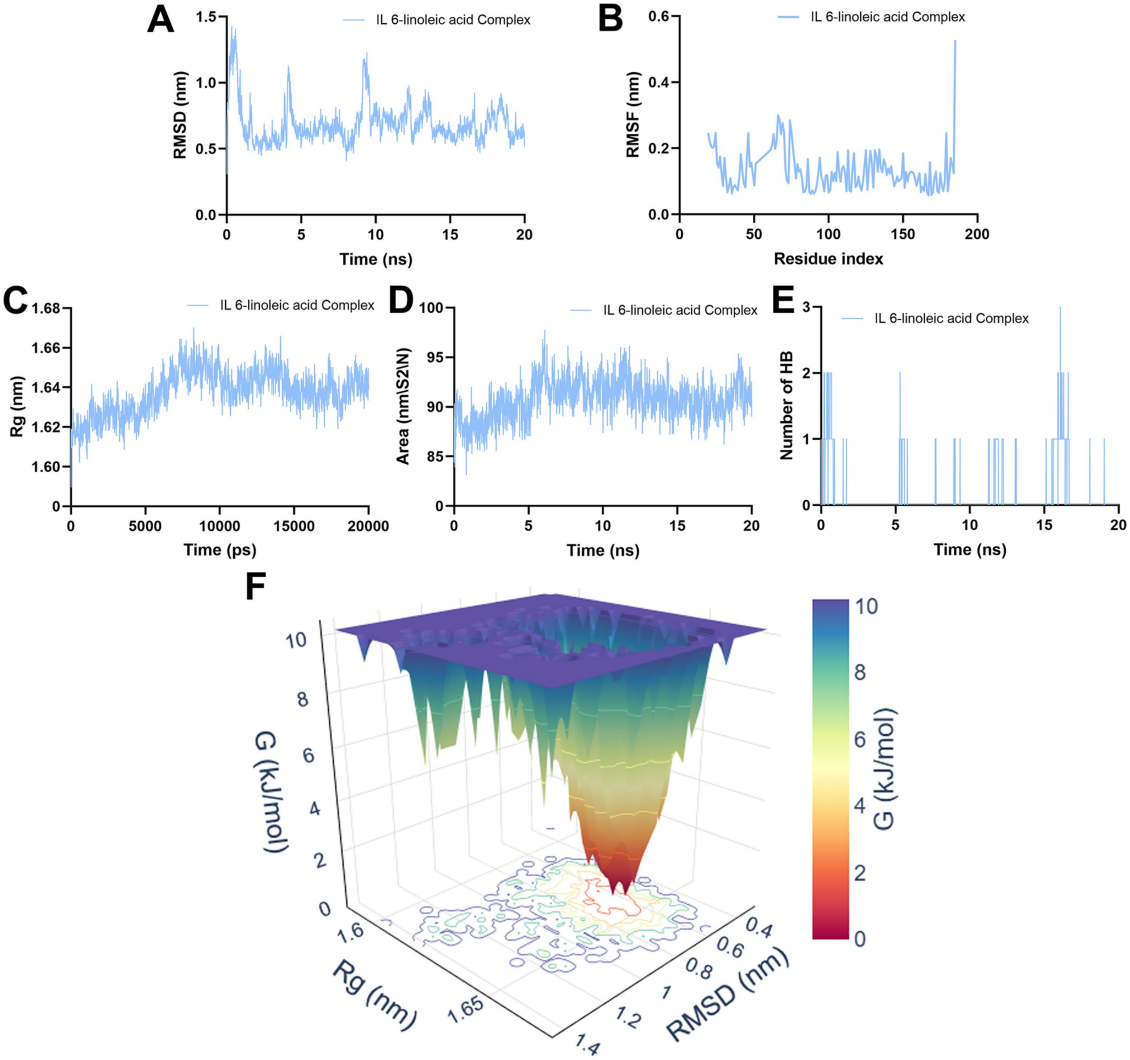
The statistical data of LA and IL-6 simulated by MD simulation. **(A)** The dynamic stability of the complexes assessment by employing the RMSD analysis. **(B)** RMSF analysis evaluated the mobility of amino acid residues during the simulation. **(C)** The stability of the IL-6-LA complex biological system was analyzed along MD trajectories by calculating the structural compactness of biomolecules using the Rg. **(D)** The surface area of the protein exposed to the solvent was analyzed during the simulation using the SASA plot. **(E)** Number of hydrogen bonds of the IL-6-LA complex formed during the computational simulations. **(F)** The conformational behavior of the IL-6-LA complex was employed by Gibbs FEL.

RMSD analysis evaluated changes in the protein system (i.e., IL-6-LA complex) following ligand interaction. After 100 ns of MD simulation, the crystal stability of the complex was assessed based on RMSD analysis. Generally, fluctuations within 2 Å are considered indicative of system equilibrium (0.1 nm = 1 Å). The average RMSD value for the IL-6-LA complex throughout the simulation was 0.6902 Å. During the initial 20 ns of the simulation, fluctuations were observed due to mechanical impact, after which a dynamically stable state was attained (Figure 8A).

RMSF analysis evaluated the mobility of amino acid residues during the simulation, concentrating on the fluctuations at the residue level within the protein-ligand complex to assess the conformational stability after the binding of the target protein and the ligand. Significant changes in RMSF indicated increased protein flexibility, whereas minor RMSF variations suggested reduced flexibility and enhanced stability. The results indicated that under constrained conditions, the IL-6-LA complex exhibited low RMSF values and stable conformations throughout the 20 ns simulation (Figure 8B).

The Rg is a parameter used to assess the stability of a complex biological system along MD trajectories by calculating the structural compactness of biomolecules. The Rg of the complex after the MD simulation was determined to assess whether the complex was stably folded or unfolded. If the Rg value remains relatively consistent throughout the MD simulation, the structure is considered to be stably folded; otherwise, it is deemed unfolded. The figure showed that the Rg of the IL-6-LA complex increased after the simulation started and then gradually stabilized, with an average Rg value of 1.6376 nm, indicating good stability (Figure 8C).

The SASA plot showed the surface area of the protein exposed to the solvent during the simulation. The SASA value represented the exposure of hydrophobic residues in the IL-6-LA complex to the solvent, which was correlated with its flexibility or structural changes. The average SASA value was 90.7385 nm^2^/N. When combined with the Rg analysis, this confirmed the relatively stable and flexible structure of the IL-6-LA complex. During the 50 ns simulation, we also evaluated the number of hydrogen bonds formed within the IL-6-LA complex. The results indicated that the complex forms up to three hydrogen bonds (Figures 8D,E).

As shown in Figure 8F, the FEL plot revealed a narrowly confined deep energy well-represented by a red/yellow funnel-shaped region-within the RMSD range of 0.4–0.8 nm and Rg range of 1.4–1.65 nm. The landscape was characterized by a single dominant basin rather than multiple minima, indicating that the IL-6-LA complex did not undergo major transitions between distinct conformational states during the simulation. Instead, the system exhibited limited fluctuations around a thermodynamically stable conformational cluster, reflecting high structural stability. The Gibbs free energy at the global minimum corresponding to the most intense red/yellow region was significantly lower (by approximately 10 kJ/mol) than that of adjacent areas. This deep energy well suggested that escaping this stable conformational state required considerable energy input, further supporting the complex’s conformational rigidity. Within this energy basin, a broad minimum was observed rather than a sharp peak, implying that the dominant conformation permitted small-amplitude fluctuations without substantial energy penalties. Overall, the FEL analysis indicated that the IL-6-LA complex adopted a thermodynamically stable, compact, and well-defined state during the simulation. This low-free-energy profile provided computational evidence for LA’s role in stabilizing IL-6 and forming a persistent complex, offering important insights into the mechanistic basis of LA’s bioactivity.

### 3.7 *In vitro* experimental verification results

#### 3.7.1 Effects of CCPI on cell viability

**Figure 9:**
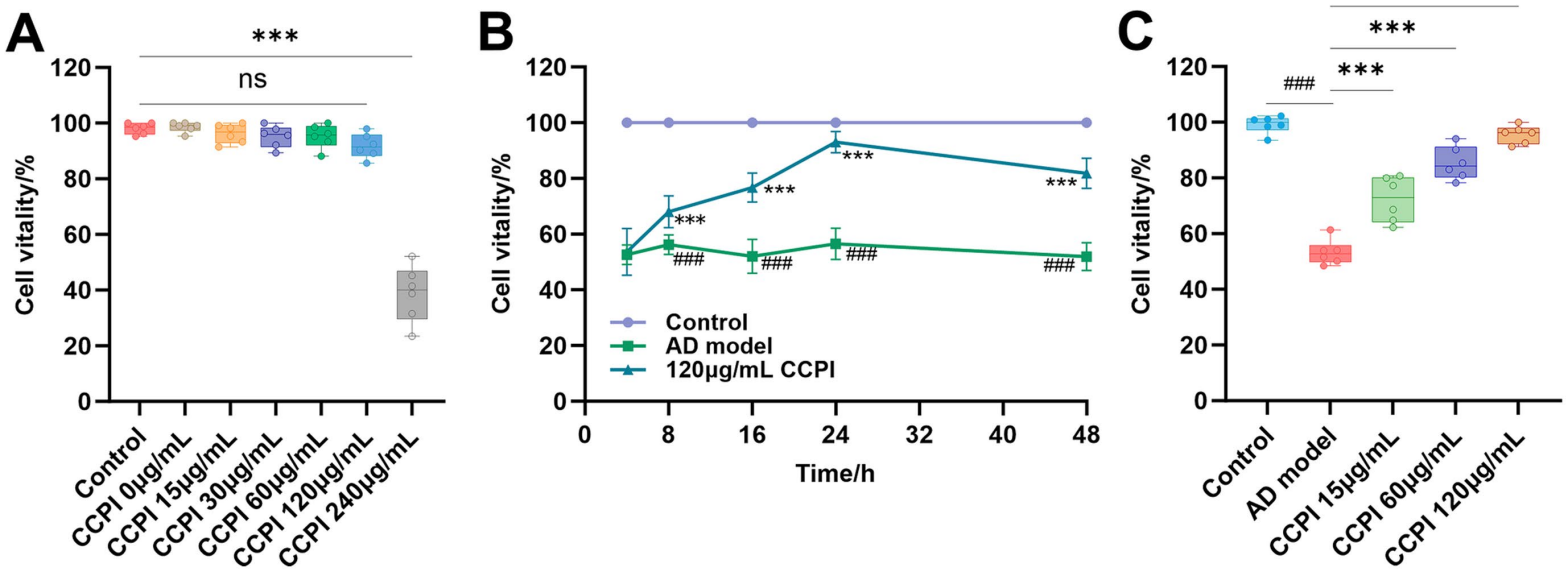
Effects of CCPI on cell viability in the AD model cells. **(A)** Dose–response graphics showing cell viability after treatment with 0, 15, 30, 60, 120, and 240 μg/mL CCPI for 24 h. **(B)** Time-course analysis of 120 μg/mL CCPI intervention at 4, 8, 12, 24, and 48 h. **(C)** Comparative effects of 15, 60, and 120 μg/mL CCPI on cell viability in the AD model after 24 h. Data are presented as mean ± SD (*n* = 6). ^###^*p* < 0.001 vs. the Control group; ^***^p < 0.001 vs. the AD model group; compared with the multiple-group, ^ns^*p* > 0.05.

To define the optimal concentration and exposure window of CCPI, we employed the CCK-8 assays to assess cell viability. CCK-8 assays demonstrated that CCPI did not suppress cell viability in BV2 microglial cells at concentrations below 120 μg/mL (Table 3; Supplementary Table 6; Figure 9A). Time-course analysis showed that cell viability in the AD group remained significantly lower than that in the Control group at each interval (all *p* < 0.001). Treatment with 120 μg/mL CCPI significantly increased cell viability after 8 h, and it continued to increase at 16 h and 24 h, peaking at 24 h (both *p* < 0.001 vs. AD group; Table 4; Supplementary Table 7; Figure 9B). Therefore, we designated 15, 60, 120 μg/mL as the administration concentration and 24 h as the optimal administration time of CCPI for the subsequent *in vitro* experiments.

**Table 3:** Effects of CCPI on cell viability at different concentrations ($\bar{x}$ ± *s*).

Group	*n*	Cell viability (%)
Control group	6	100.00 ± 0.00
0 μg/mL CCPI group	6	100.00 ± 0.00
15 μg/mL CCPI group	6	96.25 ± 3.45
30 μg/mL CCPI group	6	95.22 ± 3.88
60 μg/mL CCPI group	6	95.25 ± 4.22
120 μg/mL CCPI group	6	91.80 ± 4.41
240 μg/mL CCPI group	6	38.77 ± 10.14^###^

Compared with the Control group, ^###^*p* < 0.001.

**Table 4:** Effects of 120 μg/mL CCPI on cell viability at different time points in AD model cells ($\bar{x}$ ± *s*).

Group	*n*	4 h	8 h	16 h	24 h	48 h
Control group	6	100.00 ± 0.00%	100.00 ± 0.00%	100.00 ± 0.00%	100.00 ± 0.00%	100.00 ± 0.00%
AD model group	6	52.70 ± 3.58%^###^	56.30 ± 3.52%^###^	52.06 ± 6.09%^###^	56.58 ± 5.61%^###^	51.98 ± 5.04%^###^
120 μg/mL CCPI group	6	53.64 ± 8.42%^***^	68.08 ± 5.71%^***^	76.72 ± 5.25%^***^	93.03 ± 3.82%^***^	81.88 ± 5.39%^***^

Compared with the Control group, ^###^*p* < 0.001; compared with the AD model group, ^***^p < 0.001.

Additionally, CCPI restored the viability of Aβ-damaged BV2 microglial cells in a dose-dependent manner. Among them, 120 μg/mL CCPI was the most effective, restoring the survival rate to ≥93% (Table 5; Supplementary Table 8; Figure 9C).

**Table 5:** Effects of CCPI on cell viability at different concentrations in AD model cells ($\bar{x}$ ± *s*).

Group	*n*	Cell viability (%)
Control group	6	100.00 ± 0.00
AD model group	6	53.26 ± 4.53^###^
15 μg/mL CCPI group	6	72.29 ± 8.06^***^
60 μg/mL CCPI group	6	85.32 ± 5.91^***^
120 μg/mL CCPI group	6	95.59 ± 3.19^***^

Compared with the Control group, ^###^*p* < 0.001; compared with the AD model group, ^***^p < 0.001.

#### 3.7.2 Effects of CCPI on pro-inflammatory cytokines in the AD model cells

**Figure 10:**
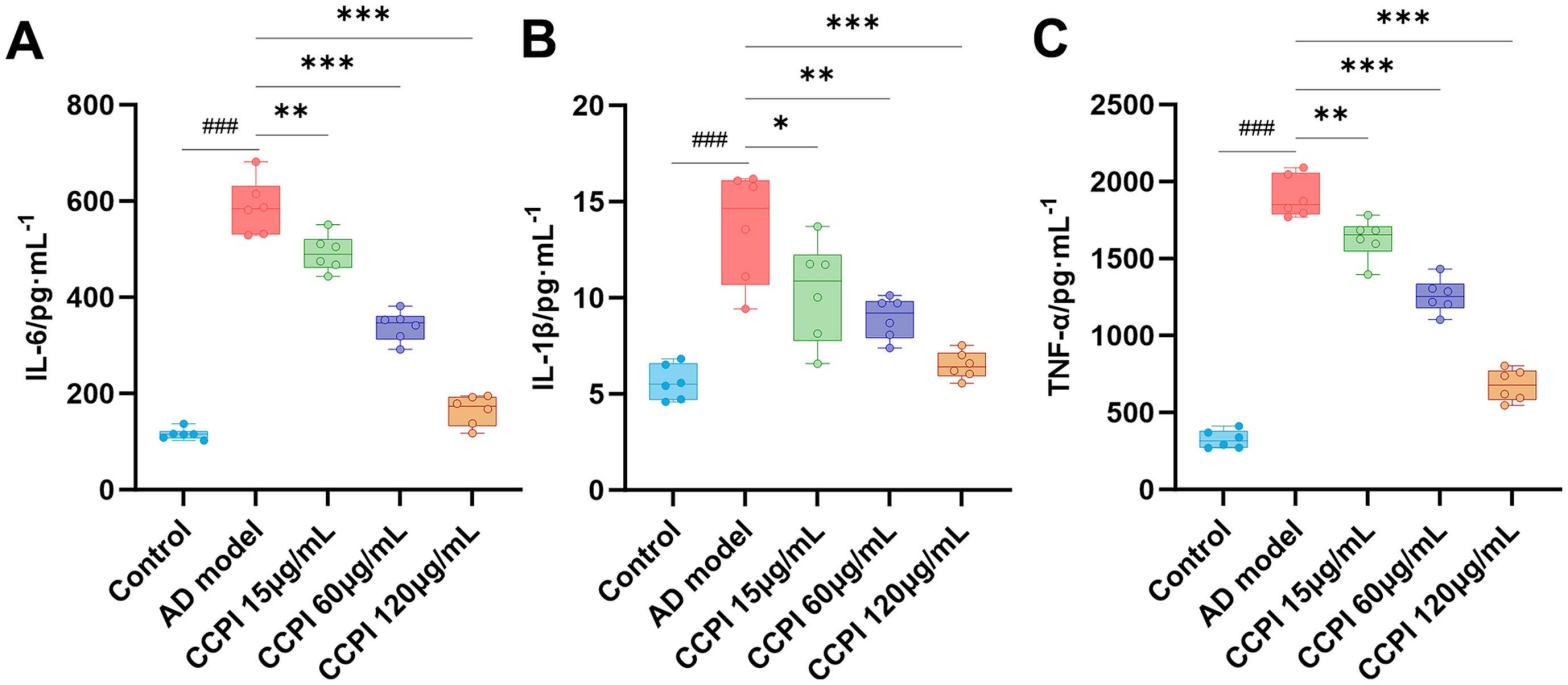
Effect of CCPI on pro-inflammatory cytokines in the AD model cells. **(A)** CCPI down-regulated the levels of IL-6. **(B)** CCPI down-regulated the levels of IL-1β. **(C)** CCPI down-regulated the levels of TNF-α. Data are presented as mean ± SD (*n* = 6). ^###^*p* < 0.001, ^##^*p* < 0.01, ^#^*p* < 0.05 compared with the Control group. ^***^p < 0.001, ^**^p < 0.01, ^*^p < 0.05 compared with the AD group.

To determine the effect of CCPI on pro-inflammatory cytokines in the AD model cells, an ELISA assay was employed. As shown in Table 6 (Supplementary Table 9) and Figures 10A–C, the levels of IL-6, IL-1β, and TNF-α in the AD model group were markedly elevated compared with those in the Control group (all *p* < 0.001), implicating a pronounced pro-inflammatory response triggered by Aβ_25-35_ in BV2 microglial cells.

**Table 6:** Effects of CCPI on pro-inflammatory cytokines levels in AD model cells ($\bar{x}$ ± *s*).

Group	*n*	IL-6 (pg·mL^−1^)	IL-1β (pg·mL^−1^)	TNF-α (pg·mL^−1^)
Control group	6	115.84 ± 11.63	5.61 ± 0.91	325.66 ± 57.87
AD model group	6	587.60 ± 57.00^###^	13.69 ± 2.87^###^	1,900.97 ± 135.59^###^
15 μg/mL CCPI group	6	492.04 ± 38.04^***^	10.32 ± 2.62^***^	1,628.44 ± 129.69^***^
60 μg/mL CCPI group	6	340.04 ± 31.27^***^	8.96 ± 1.08^***^	1,258.05 ± 111.54^***^
120 μg/mL CCPI group	6	164.95 ± 31.27^***^	6.49 ± 0.71^***^	677.08 ± 103.99^***^

Compared with the Control group, ^###^*p* < 0.001; compared with the AD model group, ^***^p < 0.001.

However, these effects were suppressed by CCPI. After treatment with CCPI, the levels of IL-6, IL-1β, and TNF-α decreased significantly in a dose-dependent manner compared with those in the AD model group (all *p* < 0.001).

#### 3.7.3 CCPI promotes the transformation of microglia from a pro-inflammatory state to an anti-inflammatory state

**Figure 11:**
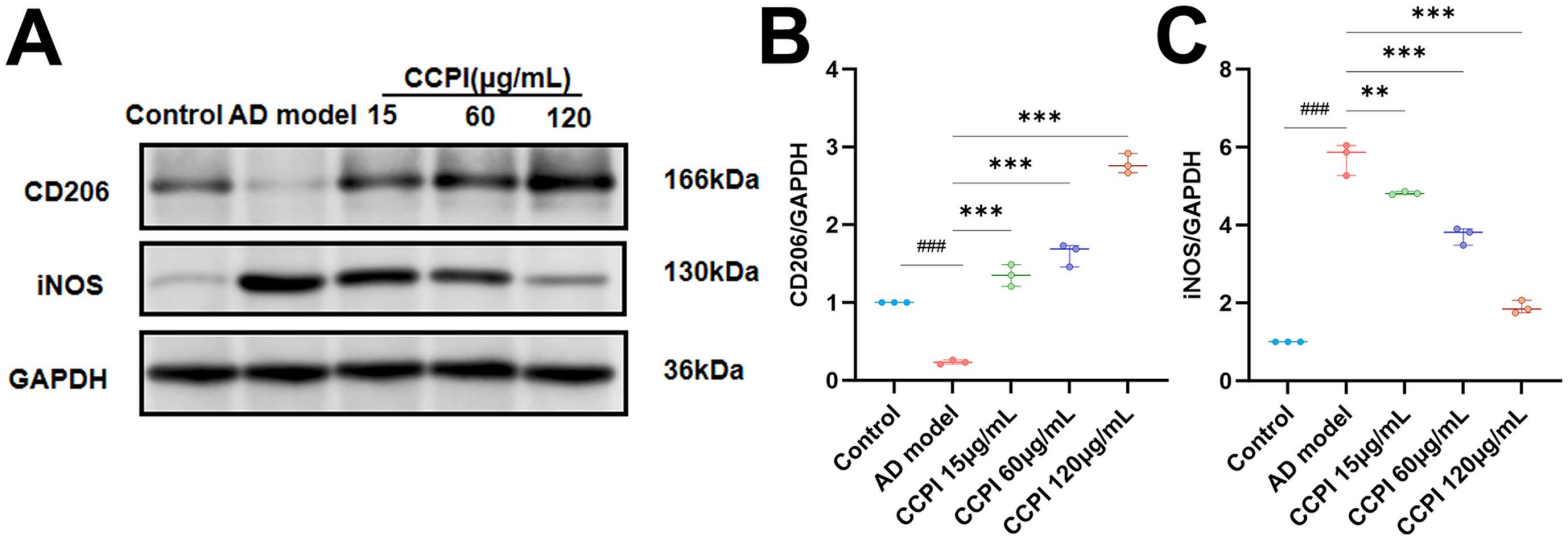
Effects of CCPI on the expression of iNOS and CD206 proteins. **(A)** Representative Western blot bands showed the expression of iNOS and CD206 after CCPI treatment. **(B,C)** Quantitative analysis of the iNOS/GAPDH and CD206/GAPDH ratios in the CCPI-treated groups, respectively. Data are presented as mean ± SD (*n* = 3). ^###^*p* < 0.001 vs. Control group; ^***^p < 0.001 and ^**^p < 0.01 vs. AD model group.

To assess the activation state of microglia induced by Aβ_25-35_, we examined the expression of markers associated with pro-inflammatory (iNOS) and anti-inflammatory/repair (CD206) responses by Western blot. Relative protein expression levels of iNOS and CD206 remained at baseline levels in the Control group. Compared with the Control group, iNOS protein levels were significantly elevated, and CD206 protein levels were significantly decreased in the AD model group (*p* < 0.001). However, compared with the AD model group, the protein levels of iNOS were reduced (15 μg/mL CCPI, *p* < 0.01; 60, 120 μg/mL CCPI, *p* < 0.001), while those of CD206 were increased (*p* < 0.001) by CCPI in a dose-dependent manner. These findings indicated that CCPI can promote the transformation of microglia from a pro-inflammatory state to an anti-inflammatory state (Figures 11A–C; Supplementary Figure 1; Table 7; Supplementary Table 10).

**Table 7:** Effect of CCPI on the expression of iNOS and CD206 proteins in AD model cells ($\bar{x}$ ± *s*).

Group	*n*	iNOS/GADPH	CD206/GADPH
Control group	3	1.00 ± 0.00	1.00 ± 0.00
AD model group	3	5.73 ± 0.40^###^	0.23 ± 0.03^###^
15 μg/mL CCPI group	3	4.82 ± 0.04^**^	1.35 ± 0.14^***^
60 μg/mL CCPI group	3	3.73 ± 0.22^***^	1.63 ± 0.15^***^
120 μg/mL CCPI group	3	1.89 ± 0.17^***^	2.78 ± 0.13^***^

Compared with the Control group, ^###^*p* < 0.001; compared with the AD model group, ^***^p < 0.001 and ^**^p < 0.01.

#### 3.7.4 Effects of CCPI on the IL-6/STAT3/VEGF signaling pathway in AD model cells

**Figure 12:**
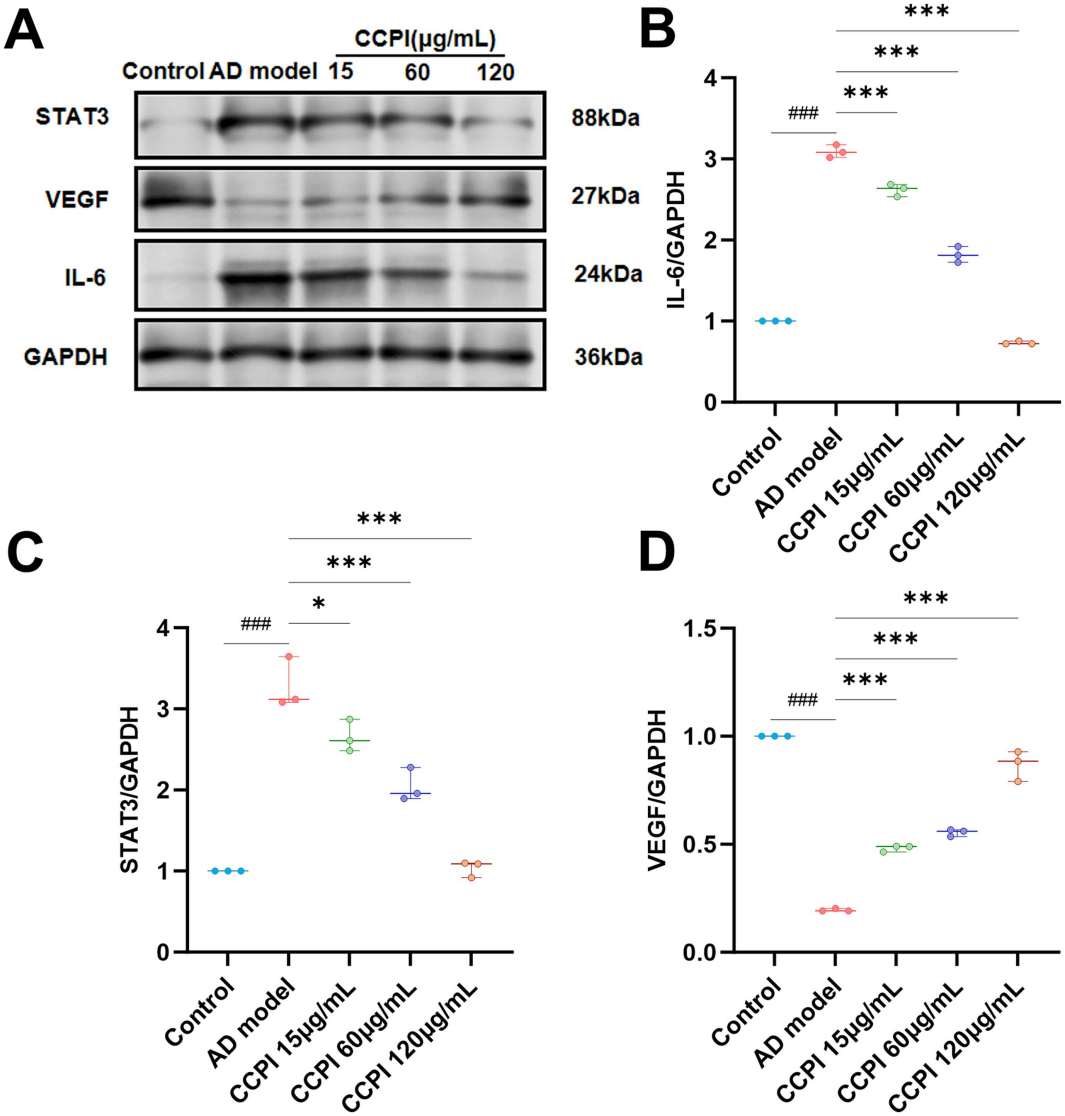
Effects of CCPI on the expression of IL-6/STAT3/VEGF signaling pathway. **(A)** Western blot showed the expression of the IL-6/STAT3/VEGF pathway after CCPI treatment. **(B–D)** Quantitative analysis of the IL-6/GAPDH, STAT3/GAPDH, and VEGF/GAPDH ratios in the CCPI-treated groups, respectively. Data are presented as mean ± SD (*n* = 3). ^###^*p* < 0.001 vs. ontrol group; ^***^p < 0.001, ^**^p < 0.01 and ^*^p < 0.05 vs. AD model group.

Network pharmacology analyses suggested that CCPI may exert therapeutic effects on AD by modulating the HIF-1 signaling pathway. To validate the mechanism in AD model cells, we hypothesized, based on our network pharmacology results identifying IL-6 as an upstream target of the HIF-1 pathway, that CCPI treats AD by regulating the IL-6/STAT3/VEGF signaling pathway. This hypothesis was tested using Western blot analysis. The protein expression levels of IL-6 and STAT3 were significantly upregulated, whereas those of VEGF were markedly downregulated in the AD model group. As predicted, treatment with CCPI substantially decreased the protein expression levels of IL-6 (*p* < 0.001) and STAT3 (15 μg/mL CCPI, *p* < 0.05; 60 and 120 μg/mL CCPI, *p* < 0.001), and increased VEGF levels compared to the AD group (*p* < 0.001). These effects were dose-dependent (Figures 12A–D; Supplementary Figure 2; Table 8; Supplementary Table 11).

**Table 8:** Effect of CCPI on the expression of IL-6/STAT3/VEGF pathway proteins in AD model cells ($\bar{x}$ ± *s*).

Group	*n*	IL-6/GADPH	STAT3/GADPH	VEGF/GADPH
Control group	3	1.00 ± 0.00	1.00 ± 0.00	1.00 ± 0.00
AD model group	3	3.09 ± 0.08^###^	3.28 ± 0.32^###^	0.20 ± 0.01^###^
15 μg/mL CCPI group	3	2.62 ± 0.08^***^	2.66 ± 0.20^*^	0.48 ± 0.01^***^
60 μg/mL CCPI group	3	1.82 ± 0.10^***^	2.04 ± 0.20^***^	0.55 ± 0.02^***^
120 μg/mL CCPI group	3	0.73 ± 0.02^***^	1.04 ± 0.10^***^	0.87 ± 0.07^***^

Compared with the Control group, ^###^*p* < 0.001; compared with the AD model group, ^***^p < 0.001, ^**^p < 0.01, and ^*^p < 0.05.

#### 3.7.5 The potential active component LA suppresses the expression of IL-6 in AD model cells

**Figure 13:**
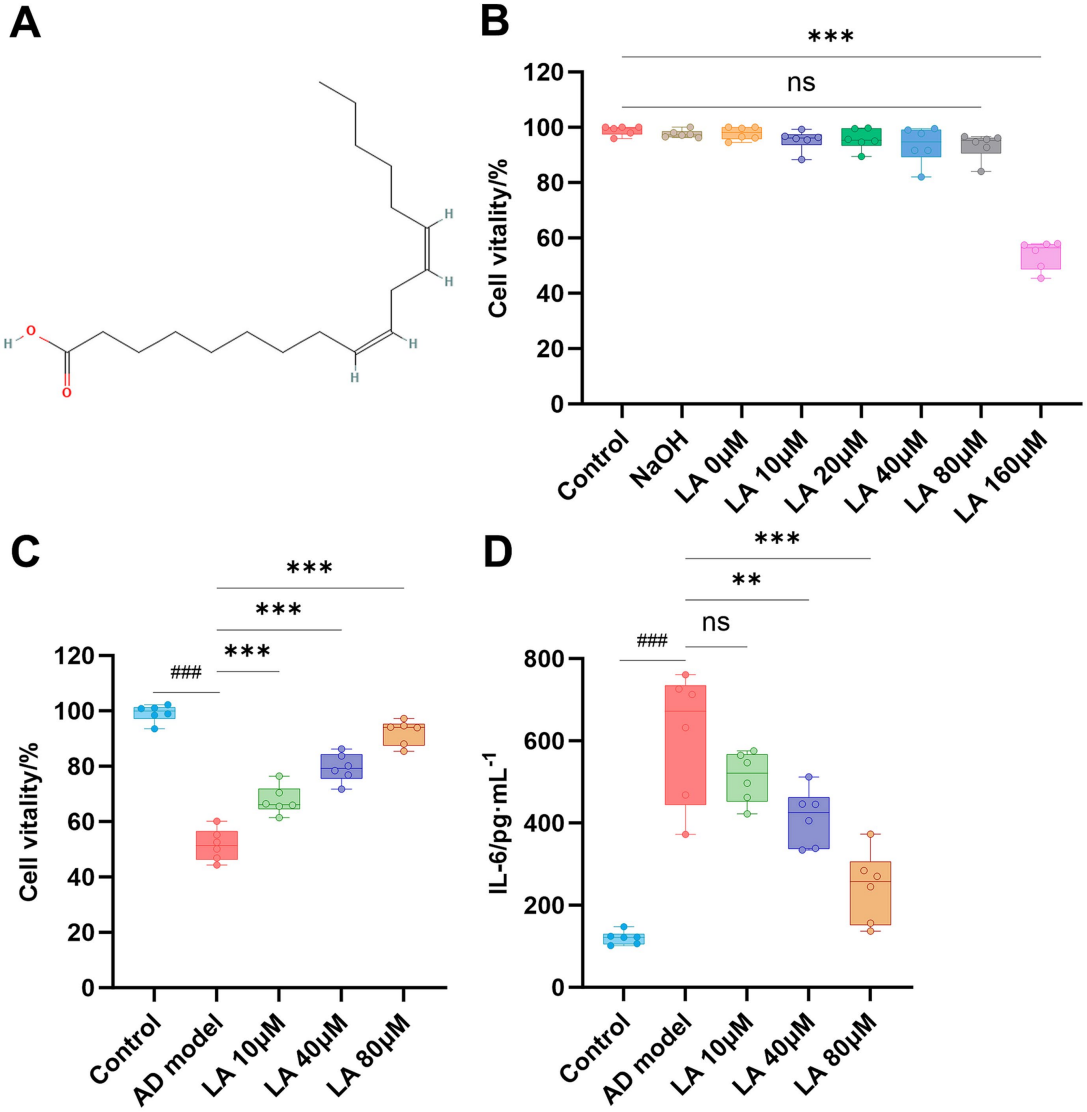
Effect of LA on IL-6 levels in the AD model cells as determined by ELISA. **(A)** Chemical structure of LA. **(B)** Dose–response graphs showing cell viability after treatment with 0, 10, 20, 40, 80, and 160 μM LA for 24 h. **(C)** Effects of 10, 40, and 80 μM LA on cell viability in the AD model cells. **(D)** LA suppresses IL-6 levels in the AD model cells. Data are presented as mean ± SD (*n* = 6). ^###^*p* < 0.001 vs. the Control group; ^***^p < 0.001, ^**^p < 0.01, and ^ns^*p* > 0.05 compared with the AD group; compared with the multiple-group, ^ns^*p* > 0.05.

CCK-8 assays showed that concentrations of LA below 80 μM did not obviously reduce cell viability in BV2 microglial cells. However, when the LA concentration reached 160 μM, the cell viability decreased significantly (Table 9; Supplementary Table 12; Figure 13B). Thus, we selected 10, 40, and 80 μM of LA as the concentrations for the subsequent *in vitro* experiments. Meanwhile, the results in Table 10 (Supplementary Table 13) and Figure 13C showed that all LA-treated groups exhibited a concentration-dependent, significantly increased cell viability compared with the AD model group (*p* < 0.001).

**Table 9:** Effects of LA on cell viability in AD model cells at different concentrations ($\bar{x}$ ± *s*).

Group	*n*	Cell viability (%)
Control group	6	100.00 ± 0.00
NaOH group	6	97.57 ± 1.36
0 μM LA group	6	100.00 ± 0.00
10 μM LA group	6	95.37 ± 3.69
20 μM LA group	6	95.69 ± 3.79
40 μM LA group	6	93.62 ± 6.67
80 μM LA group	6	93.33 ± 4.75
160 μM LA group	6	53.96 ± 5.19^###△△△^

Compared with the Control group, ^###^*p* < 0.001; compared with the NaOH group (NaOH is the solvent of LA), ^△△△^*p* < 0.001.

**Table 10:** Effects of LA on cell viability in AD model cells at different concentrations ($\bar{x}$ ± *s*).

Group	*n*	Cell viability (%)
Control group	6	100.00 ± 0.00
AD model group	6	51.56 ± 5.74^###^
10 μM LA group	6	67.72 ± 5.15^***^
40 μM LA group	6	79.51 ± 5.16^***^
80 μM LA group	6	92.27 ± 4.49^***^

Compared with the Control group, ^###^*p* < 0.001; compared with the AD model group, ^***^p < 0.001.

As shown in Figure 13D and Table 11 (Supplementary Table 14), the levels of IL-6 in the group treated with LA were significantly decreased in a dose-dependent manner compared with those in the AD model group (10 μM LA, *p* > 0.05; 40 μM LA, *p* < 0.01; 80 μM LA, *p* < 0.001). The results verified the prediction of network pharmacology, confirming that LA may be the active ingredient targeting IL-6 in CCPI.

**Table 11:** Effects of LA on IL-6 levels in AD model cells measured by ELISA ($\bar{x}$ ± *s*).

Group	*n*	IL-6 (pg·mL^−1^)
Control group	6	120.54 ± 16.31
AD model group	6	612.17 ± 157.34^###^
10 μM LA group	6	511.51 ± 61.28
40 μM LA group	6	413.83 ± 69.10^**^
80 μM LA group	6	244.37 ± 87.33^***^

Compared with the Control group, ^###^*p* < 0.001; compared with the AD model group, ^***^p < 0.001 and ^**^p < 0.01.

#### 3.7.6 Comparison of the effects of CCPI and LA on IL-6 secretion, STAT3 phosphorylation, and microglial functional biomarkers expression in AD model cells

**Figure 14:**
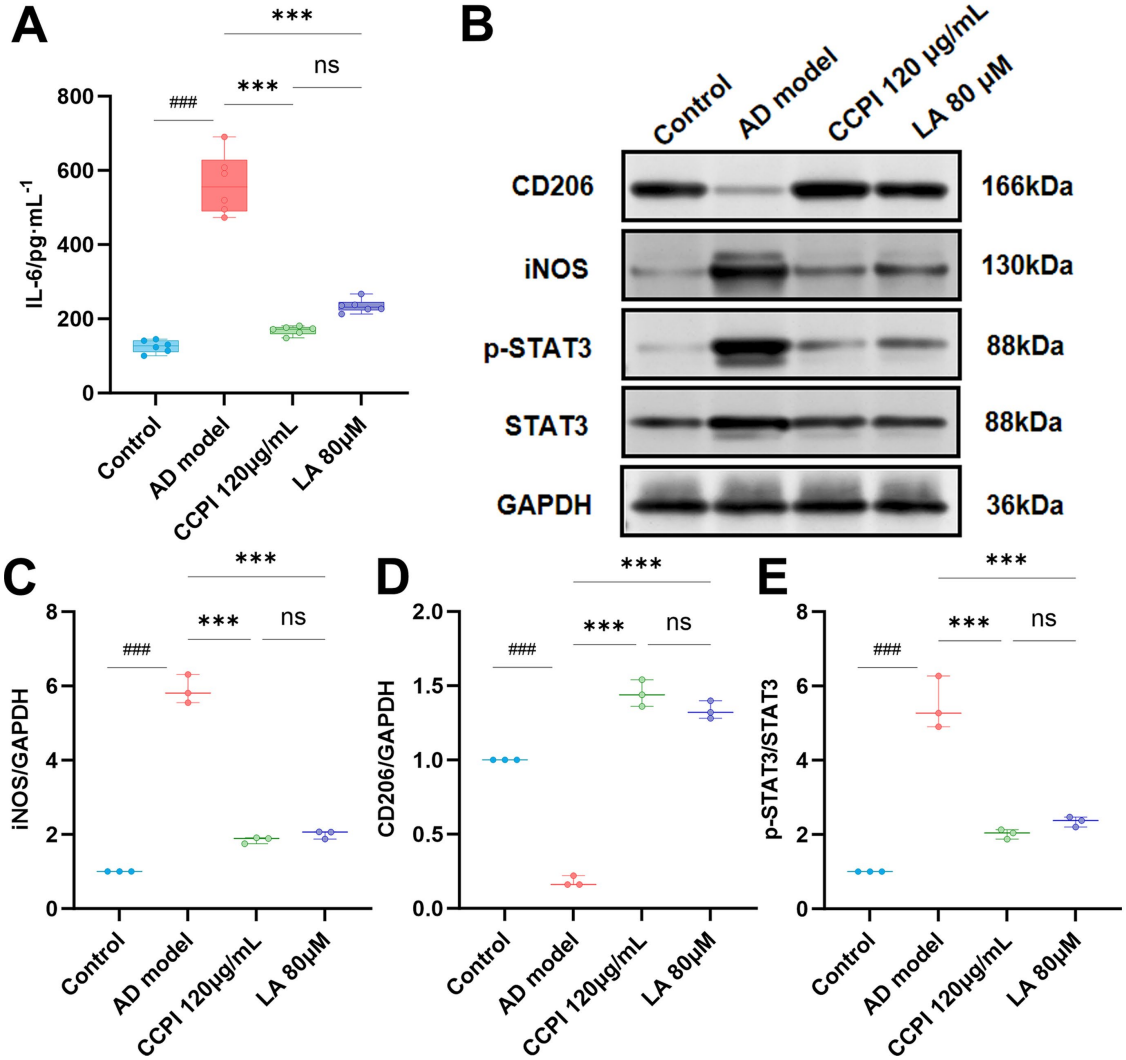
Comparison of the effects of CCPI and LA on IL-6 secretion, STAT3 phosphorylation, and the expression of markers associated with pro-inflammation (iNOS) and anti-inflammation/repair (CD206) in AD model cells. **(A)** IL-6 levels in the AD model cells were measured by ELISA. **(B)** Western blot showed the expression of iNOS, CD206, STAT3 phosphorylation, and STAT3 after CCPI and LA treatment. **(C–E)** Quantitative analysis of the iNOS/GAPDH, CD206/GAPDH, and STAT3 phosphorylation/STAT3 ratios in the CCPI and LA-treated groups, respectively. Data are presented as mean ± SD (*n* = 3). ^###^*p* < 0.001 vs. control group; ^***^p < 0.001, **p < 0.01 and ^*^p < 0.05 vs. AD model group; ^ns^*p* > 0.05 compared with the CCPI group; compared with the LA group, ^ns^*p* > 0.05.

As shown in Figure 14 (Supplementary Figure 3) and Table 12 (Supplementary Table 15), Table 13 (Supplementary Table 16), both CCPI (120 μg/mL) and its constituent LA (80 μM) significantly alleviated AD-related pathology by reducing IL-6, STAT3 phosphorylation, and iNOS, and by increasing CD206 levels (all *p* < 0.001 vs. AD model). Furthermore, there was no statistically significant difference in the efficacy between CCPI and LA alone for any of these parameters (*p* > 0.05). The fact that LA achieved effects comparable to CCPI supports its role as a potential key active ingredient in neuroprotection.

**Table 12:** Effects of CCPI and LA on IL-6 levels in AD model cells measured by ELISA ($\bar{x}$ ± *s*).

Group	*n*	IL-6 (pg·mL^−1^)
Control group	6	125.61 ± 16.77
AD model group	6	562.88 ± 82.01^###^
120 μg/mL CCPI group	6	168.89 ± 11.69 ^***, ns^
80 μM LA group	6	234.58 ± 18.19^***, ns^

Compared with the Control group, ^###^*p* < 0.001; compared with the AD model group, ^***^p < 0.001 and ^**^p < 0.01; compared with the 80 μM LA group, ^ns^*p* > 0.05; compared with the 120 μg/mL CCPI group, ^ns^*p* > 0.05.

**Table 13:** Effects of CCPI and LA on the expression of iNOS, CD206, and STAT3 phosphorylation proteins in AD model cells ($\bar{x}$ ± *s*).

Group	*n*	iNOS/GADPH	CD206/GADPH	p-STAT3/STAT3
Control group	3	1.00 ± 0.00	1.00 ± 0.00	1.00 ± 0.00
AD model group	3	5.89 ± 0.39^###^	0.18 ± 0.03^###^	5.48 ± 0.71^###^
120 μg/mL CCPI group	3	1.85 ± 0.09^***, ns^	1.45 ± 0.09^***, ns^	2.02 ± 0.13^***, ns^
80 μM LA group	3	2.00 ± 0.11^***, ns^	1.33 ± 0.06^***, ns^	2.35 ± 0.14^***, ns^

Compared with the Control group, ^###^*p* < 0.001; compared with the AD model group, ^***^p < 0.001, **p < 0.01, and ^*^p < 0.05; compared with the 80 μM LA group, ^ns^*p* > 0.05; compared with the 120 μg/mL CCPI group, ^ns^*p* > 0.05.

## 4 Discussion

The current treatment of AD faces the dual challenges of complex pathology and limited efficacy of single-target drugs. CCPI, a compound TCM with demonstrated clinical anti-inflammatory effects, has shown favorable efficacy and safety in orthopedic and cerebrovascular diseases (Qi et al., 2020; Wei et al., 2016; Xu et al., 2013). It can reduce inflammatory factors such as TNF-α and IL-6 and promote neural repair (Zhang et al., 2020; Chen, 2025; Liu, 2012). These features align closely with neuroinflammation, a core pathological process in AD, suggesting considerable therapeutic potential of CCPI for AD. However, its direct anti-AD effects and underlying molecular mechanisms remain unclear. Therefore, we systematically explored CCPI’s anti-AD effects by employing network pharmacology, molecular docking, MD simulation, and vitro experiments.

Among the results of network pharmacology, 50 disease targets for AD were ultimately identified via Venn diagrams. The common 50 targets of AD can be classified into six major pathological core modules: The γ-secretase complex [PSEN1, PSEN2 (Lanoiselée et al., 2017), APH1A, APH1B (Marlow et al., 2003), PSENEN (Albani et al., 2007), NCSTN (Yan et al., 2024)] directly drives abnormal Aβ production. The neuroinflammatory network [IL6 (Lin et al., 2022), PTGS2 (Rangaraju et al., 2018), NOS2 (Kalinin et al., 2006), ICAM1 (Wiciński et al., 2019), ITGB2 (Hok et al., 2026), CTSG (Dhanavade et al., 2013), ALOX5 (Li et al., 2017)] forms a positive feedback loop with Aβ formation, accelerating neural damage. Synaptic and neurotransmitter dysregulation [GRIA1 (Tong et al., 2025), DRD2 (Liu et al., 2023), SLC6A4 (Tajeddinn et al., 2016), CNR1 (Toledano and Akirav, 2025), ADORA2A (Orr et al., 2018), BCHE (Fidan et al., 2022)] leads to cognitive and behavioral symptoms. Lipid metabolism disorders [HMGCR (Azizi Dariuni et al., 2023), PPARA (Luo R. et al., 2020), PPARG (Ge et al., 2025), NR1H3 (Natunen et al., 2013)] affect Aβ clearance and membrane homeostasis. Sex hormone and stress signaling imbalance [ESR1, ESR2 (Pietrzak-Wawrzyńska et al., 2025), AR (Kikuchi et al., 2020), CYP19A1, CYP17A1 (Armstrong et al., 2020), NR3C1, NR3C2 (Porter et al., 2025)] explains gender differences and lack of neuroprotection. The signaling and oxidative stress regulatory network [MAPK1/3/14 (Lee et al., 2019, Ji et al., 2019), FYN (Um and Strittmatter, 2013), PTPN1/11 (Li et al., 2023), PLA2G4A (Sanchez-Mejia et al., 2008), G6PD (Correas et al., 2024), GLUL (Ibrahim et al., 2020), TERT (Bignoux et al., 2019), PREP (Falkevall et al., 2006)] serves as a fundamental amplifier, sustaining the progressive deterioration of pathological processes. Among the 11 potential targets in AD, IL6 may be the key target (Table 2; Figure 2B), indicating that this target may be primary target for CCPI treatment of AD and play a key role in its therapeutic effects. It is important to note that while high-degree nodes in a PPI network may indicate potential functional centrality, they are not automatically definitive therapeutic targets. The selection of IL-6 as the subject of further investigation was based on its top ranking in this preliminary topological screening (degree value = 35), combined with its well-documented role in the neuroinflammatory pathology of AD. Therefore, targeting IL-6 specifically could be beneficial for treating AD. IL-6 is a pro-inflammatory cytokine predominantly secreted by activated microglia (Dhapola et al., 2021). The activity of β-site amyloid precursor protein (APP) cleaving enzyme 1 (BACE1) is enhanced by IL-6, leading to an increasing production of Aβ in the AD brain (Calsolaro and Edison, 2016). Following the interaction of Aβ with microglia, other inflammatory molecules are also translocated to the site of inflammation by chemotaxis, exacerbating neuroinflammation (Heneka et al., 2025). In AD, neuroinflammation represents a central pathological process. Aberrantly activated microglia secrete substantial amounts of pro-inflammatory cytokines and may contribute to the accumulation of Aβ, forming a vicious cycle that exacerbates disease progression (Hou et al., 2024; Cai et al., 2014). Traditionally, activated microglia have often been oversimplified into two main states: the pro-inflammatory state and the anti-inflammatory/reparative state (Zhao et al., 2019). During AD pathogenesis, sustained pro-inflammatory activation is considered a primary driver of detrimental neuroinflammation (Shemer et al., 2015), characterized by high expression of iNOS and the release of factors such as IL-1β, IL-6, and TNF-α, which cause direct neuronal injury (Lee et al., 2015; Zhang et al., 2019). In contrast, the anti-inflammatory/ reparative state is associated with the upregulation of anti-inflammatory mediators (e.g., CD206) and neurotrophic factors, which help mitigate inflammation, promote tissue repair, and provide neuroprotection (Jiang et al., 2020; Dong et al., 2019; Subhramanyam et al., 2019). Therefore, modulating microglial activation from a detrimental pro-inflammatory state toward a protective phenotype has emerged as a significant strategic direction for AD therapy (Varol et al., 2017).

In the “compound-target” network analysis of CCPI, 5 components may be the potential active components of CCPI, and LA may be the potential active ingredient. Furthermore, LA is associated with the largest number of key targets, suggesting its primary role in the prevention and treatment of AD. LA, as an unsaturated free fatty acid in the human dietary fatty acid spectrum, exerts protective effects against multiple cellular stresses, including inflammation (Mamounis et al., 2017; Katsoulieris et al., 2009; Zhang et al., 2011). Dietary fatty acids (FAs) can cross the BBB and are mostly esterified into the membrane phospholipids of neurons and glial cells, directly or through their active metabolites influencing brain functions (Bazinet and Layé, 2014). Recent findings suggest that FAs can either promote or suppress microglial inflammatory responses (Leyrolle et al., 2019). Oral administration of LA demonstrated significantly suppresses glia-mediated neuroinflammation in mouse brains (Ali et al., 2020). Similarly, conjugated linoleic acid (CLA), a group of positional and geometric isomers of LA, is a natural calpain inhibitor (Lee et al., 2013) that can alleviate age-dependent neurodegeneration (Monaco et al., 2018) and downregulate the hallmarks of AD mice (Binyamin et al., 2019; Cuciniello et al., 2022). Furthermore, dietary CLA demonstrated neuroprotective effects in an AD mouse model, including the suppression of neuroinflammation (Fujita et al., 2021). Therefore, LA is the key active ingredient of CCPI in the treatment of AD, and its mechanism of action may be achieved by inhibiting neuroinflammation mediated by microglia in the brain.

Molecular docking revealed that LA has a strong binding affinity for IL-6 (docking energies below −5.0 kcal/mol, Figure 7). To further validate these interactions, MD simulation was conducted to compensate for the shortage of experimental verification by using traditional methods such as SPR/ITC. These simulations demonstrated that the protein-ligand complexes remained structurally stable under physiological conditions. Throughout the simulation, the complexes maintained stable RMSD and RMSF values. Our results support the hypothesis that CCPI’s potential active compounds (LA) interact dynamically and stably with key target proteins (IL-6), reinforcing their potential for multi-target therapeutic effects (Figure 8). Therefore, we speculate that LA was identified as a potential active component and stable binding potential with the hub target IL-6.

In the enrichment analysis, the HIF-1 signaling pathway was the critical pathway (Figure 6). HIF-1 plays a potential neuroprotective role in AD (Correia and Moreira, 2010). Interestingly, IL-6, which serves as an upstream target of the HIF-1 pathway, is also a pivotal CCPI-AD target. As a pro-inflammatory cytokine, IL-6 is a key activator of the signal transducer and activator of STAT3 pathway, which makes it a critical contributor to neuroinflammation in AD (Chiba et al., 2009). IL-6 deficiency reduced hippocampal neuroinflammation and contributed to cognitive improvements in AD mice, an effect manifested as a significant reduction in the number of IBA1-positive microglia without affecting the population of GFAP-positive astrocytes. Peter Vandenabeele and Walter Fiers propose that amyloidogenesis is a consequence of an IL-6-mediated acute phase reaction in the brain (Vandenabeele and Fiers, 1991). IL-6 plays a role in the early formation of amyloid plaques in the brains of individuals with AD (Huell et al., 1995) and has been associated with tau phosphorylation (Quintanilla et al., 2004). A meta-analysis indicates that the IL-6-174 G/C and IL-6-572 G/C polymorphisms may influence the overall population’s susceptibility to AD. Furthermore, these polymorphisms may also affect the predisposition to AD among Asian populations (Yang et al., 2021). Serum levels of IL-6 correlate with various features of AD, including the extent of neuropathology and cognitive function (Brosseron et al., 2023). Neutralizing IL-6 and inhibiting the signaling of the signal transducer and activator of transcription 3 (STAT3) in AD mouse models have been shown to mitigate memory impairment (Lyra et al., 2021). Collectively, these findings suggest that IL-6 serves as a link between cognitive decline and peripheral metabolic changes in AD. Thus, targeting pro-inflammatory IL-6 signaling may represent a viable strategy to alleviate memory deficits associated with the AD. Furthermore, various drugs have been shown to target the STAT3 pathway in microglia, reducing the production of inflammatory mediators (Ni et al., 2023; Gao et al., 2022; Alawdi et al., 2017; Phan Van et al., 2024; Dos Santos et al., 2024). Angiogenesis is the response of endothelial cells to hypoxia and inflammation, mediated by cytokine growth factors (Zhang et al., 2023). Morphological and biochemical evidence of this process includes increased expression of angiogenic factors such as VEGF (Viallard and Larrivée, 2017). VEGF is a factor that mainly regulates angiogenesis. *In vivo*, it is produced by cells adjacent to vascular endothelial cells (Jin et al., 2002). VEGF over-expression obtained by intracerebral administration, gene transfer, or that in conditional transgenic mice improves associative memory performances, and short exposure to over-expressed VEGF is sufficient to affect cognitive function (Cao et al., 2004; Licht et al., 2011). Furthermore, in the presence of enhanced AD biomarkers in CSF, elevated VEGF levels are associated with less cognitive decline (Hohman et al., 2015), highlighting a potential neuroprotective role for VEGF in AD. These findings indicate that IL-6/STAT3/VEGF may be the key pathway to suppress neuroinflammation and alleviate AD.

*In vitro* experimental results have confirmed the potential mechanism of CCPI and its potential network-predicted components (LA) in AD model cells. These results showed that CCPI and LA could specifically ameliorate the pathological damage in Aβ-induced BV2 microglial cells. At the level of cell viability, LA increased cell viability in a concentration-dependent manner, whereas CCPI demonstrated both dose-dependent and time-dependent effects (Figures 9A,B). The optimal viability recovery was observed when AD model cells were treated with CCPI at a concentration of 120 μg/mL for 24 h (Figures 9B,C). In addition, ELISA results showed a significant increase on IL-1β, IL-6, and TNF-α levels in AD model cells. CCPI significantly suppressed the secretion of IL-1β, IL-6, and TNF-α in Aβ-induced BV2 microglia cells, further confirming its anti-neuroinflammation effects (Figure 10). Moreover, Western blot analysis revealed that CCPI significantly suppressed the levels of the pro-inflammatory marker (iNOS) while upregulating the protein expression of the anti-inflammatory/repair-associated marker (CD206) in AD model cells (Figure 11). These findings indicate that the therapeutic effects of CCPI are associated with shifting microglia from a pro-inflammatory state toward a protective/repair state. Since the elucidation of the mechanisms involved in HIF-1-mediated neuroprotection could be important for the development of effective therapies to mitigate or prevent neurodegenerative diseases, we explored the key factors of the HIF-1 signaling pathway, including IL-6, STAT3, and VEGF (Figure 12). Our results prove that CCPI reduced the protein expression levels of IL-6 and STAT3 and upregulated VEGF in AD model cells. Our research further demonstrated that CCPI is closely associated with the IL-6/STAT3/VEGF signaling pathway in the improvement of AD. In conclusion, the molecular mechanism by which CCPI exerts its anti-AD effects is by reducing neuroinflammation. This involves modulating microglial function by shifting their state from pro-inflammatory to protective, reducing levels of inflammatory cytokines, and regulating the IL-6/STAT3/VEGF signaling pathway. To further elucidate the effects through which the active ingredient LA of CCPI regulates anti-AD, we assessed the levels of IL-6, STAT3 phosphorylation, and microglial function marker expression in Aβ-induced BV2 microglial cells between CCPI and LA. The results showed a notable decrease in IL-6, phosphorylated STAT3, and iNOS protein levels in AD model cells following LA treatment, while CD206 protein levels rose (Figure 14). No disparities were observed in IL-6, STAT3 phosphorylation, and microglial function marker expression levels between CCPI and LA, indicating that LA may be a potential active component in CCPI that exerts neuroprotective effects.

While this study provides the first evidence supporting CCPI and its component LA as modulators of neuroinflammation via the IL-6/STAT3 axis, it also outlines several important directions for future research. First, in the study of microglia, the traditional M1/M2 classification model is being supplemented with more detailed and disease-specific activation state descriptions, such as DAM defined in neurodegenerative diseases. Although this study did not detect specific markers of DAM (e.g., Apoe, Trem2, Clec7a), the observed reduction in iNOS and elevation in CD206 together indicate that CCPI promotes the transition of microglia into an activated state characterized by anti-inflammatory and tissue repair properties. This may be associated with the functions of inhibiting neuroinflammation and promoting tissue repair, offering insights into the molecular mechanisms through which CCPI regulates microglial function. Second, to precisely define the therapeutic contribution of LA within the CCPI mixture, subsequent work should compare the neuroprotective efficacy of intact CCPI, an LA-enriched fraction, and purified LA. This should be coupled with absolute quantification of LA in commercial CCPI preparations and experimental validation (e.g., SPR/ITC) of the LA-IL-6 interaction predicted by our simulations. Third, the pathological complexity of AD necessitates studies beyond a single cell type. Building on evidence that CCPI can affect other neural cells, its effects should be investigated in neuron–glia co-culture systems and, crucially, in AD animal models. These models will also allow assessment of its impact on cognitive function and other AD-related pathways, such as oxidative stress and neurotransmitter systems, to fully elucidate its “multi-component, multi-target” profile. Finally, the translational potential of CCPI may be influenced by disease-state alterations in the BBB. Future studies should evaluate how AD-related neuroinflammation affects the central delivery and efficacy of CCPI’s various components, which could reveal additional synergistic mechanisms *in vivo*.

## 5 Conclusion

By integrating network pharmacology predictions with preliminary experimental validation, this study elucidates the potential mechanism of CCPI against AD. The findings indicate that CCPI likely exerts its effects via a multi-target, multi-pathway mode. LA, its potential key active component, may act by targeting the hub protein IL-6, thereby modulating the IL-6/STAT3/VEGF signaling axis. This regulation reshapes the functional state of microglia (inhibiting their pro-inflammatory responses and enhancing their reparative functions) and modulates the neuroinflammatory microenvironment, thereby exerting neuroprotective effects.

## Data Availability

The original contributions presented in the study are included in the article/Supplementary material, further inquiries can be directed to the corresponding author.

## Footnotes

1
https://tcmspw.com/tcmsp.php

2
https://www.uniprot.org/

3
https://www.drugbank.ca

4
https://www.omim.org/

5
https://www.genecards.org/

6
https://bioinfogp.cnb.csic.es/tools/venny/index.html

7
http://string-db.org

8
https://davidbioinformatics.nih.gov/

9
http://www.bioinformatics.com.cn

10
https://www.rcsb.org/

## Glossary

AD: Alzheimer’s Disease
ANOVA: Analysis of Variance
APP: Amyloid Precursor Protein
Aβ: Amyloid-β
BACE1: β-site Amyloid precursor protein Cleaving Enzyme 1
BBB: Blood Brain Barrier
BP: Biological Process
CC: Cellular Component
CCK-8: Cell Counting Kit-8
CCPI: Cervus Cucumis Polypeptide Injection
CLA: Conjugated Linoleic Acid
CNS: Central Nervous System
DAM: Disease-Associated Microglia
DAMPs: Damage Associated Molecular Patterns
DL: Drug-Likeness
DMEM: Dulbecco’s Modified Eagle Medium
ELISA: Enzyme-linked Immunosorbent Assay
FAs: Fatty Acids
FBS: Fetal Bovine Serum
FEL: Free-Energy Landscape
GO: Gene Ontology
IL-1β: Interleukin-1β
iNOS: Inducible Nitric Oxide Synthase
ITC: Isothermal Titration Calorimetry‌
KEGG: Kyoto Encyclopedia of Genes and Genomes
LA: Linoleic Acid
MAPK3: Mitogen-activated Protein Kinase 3
MD: Molecular Dynamics
MF: Molecular Function
OB: Oral Bioavailability
OD: Optical Density
PBS: Phosphate Buffer Saline
PPARG: Peroxisome Proliferator-Activated Receptor Gamma
PPI: Protein–Protein Interaction
PS: Penicillin–Streptomycin
Rg: Radius of Gyration
RIPA: Radio-Immunoprecipitation Assay
RMSD: Root Mean Square Deviation
RMSF: Root Mean Square Fluctuation
SASA: Solution Accessible Surface Area
SD: Standard Deviation
SPC: Simple Point Charge
SPR: Surface Plasmon Resonance‌
TCM: Traditional Chinese Medicine
TCMSP: Traditional Chinese Medicine Systems Pharmacology
TGF-β: Transform into Growth Factor β
TNF-α: Tumor Necrosis Factor α
