# Supplementary material for: Unraveling the anti-neuroinflammatory mechanisms of Cervus cucumis polypeptide injection in Alzheimer’s disease: insights from network pharmacology, molecular docking, molecular dynamics simulation, and experimental validation
Source: Front Aging Neurosci. 2026 Apr 22;18:1797302. doi: 10.3389/fnagi.2026.1797302 (PMC13153433; doi:10.3389/fnagi.2026.1797302)
Supplement: Supplementary file 1 [file Data_Sheet_1.docx]

Supplementary Material

Supplementary Figures:

**1. Supplementary Figure 1. Uncropped full-length images of the gel and blot for iNOS, CD206, and GAPDH from the same gel.**

**
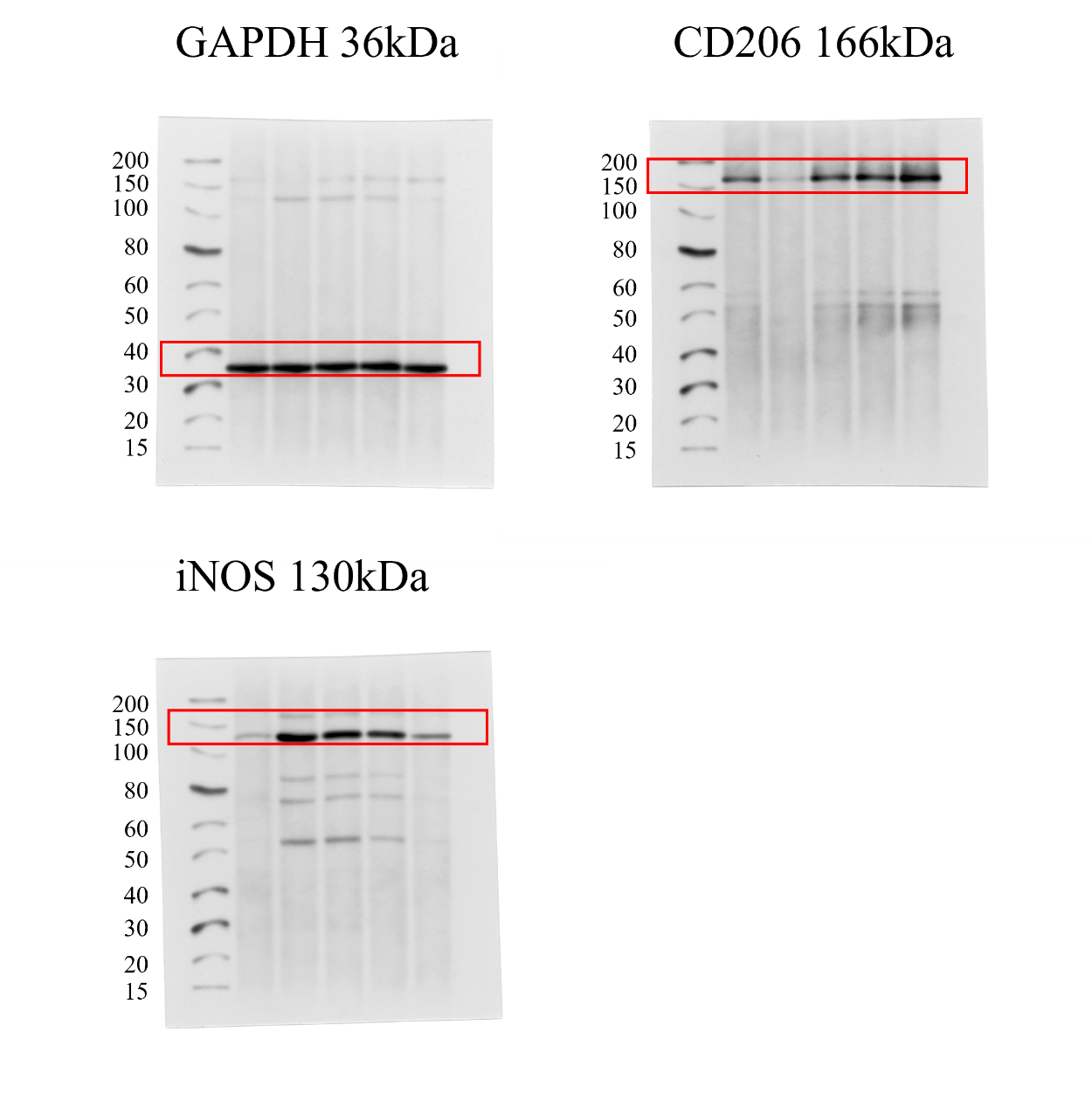
**

**2. Supplementary Figure 2. Uncropped full-length images of the gel and blot for IL-6, STAT3, VEGF, and GAPDH from the same gel.**


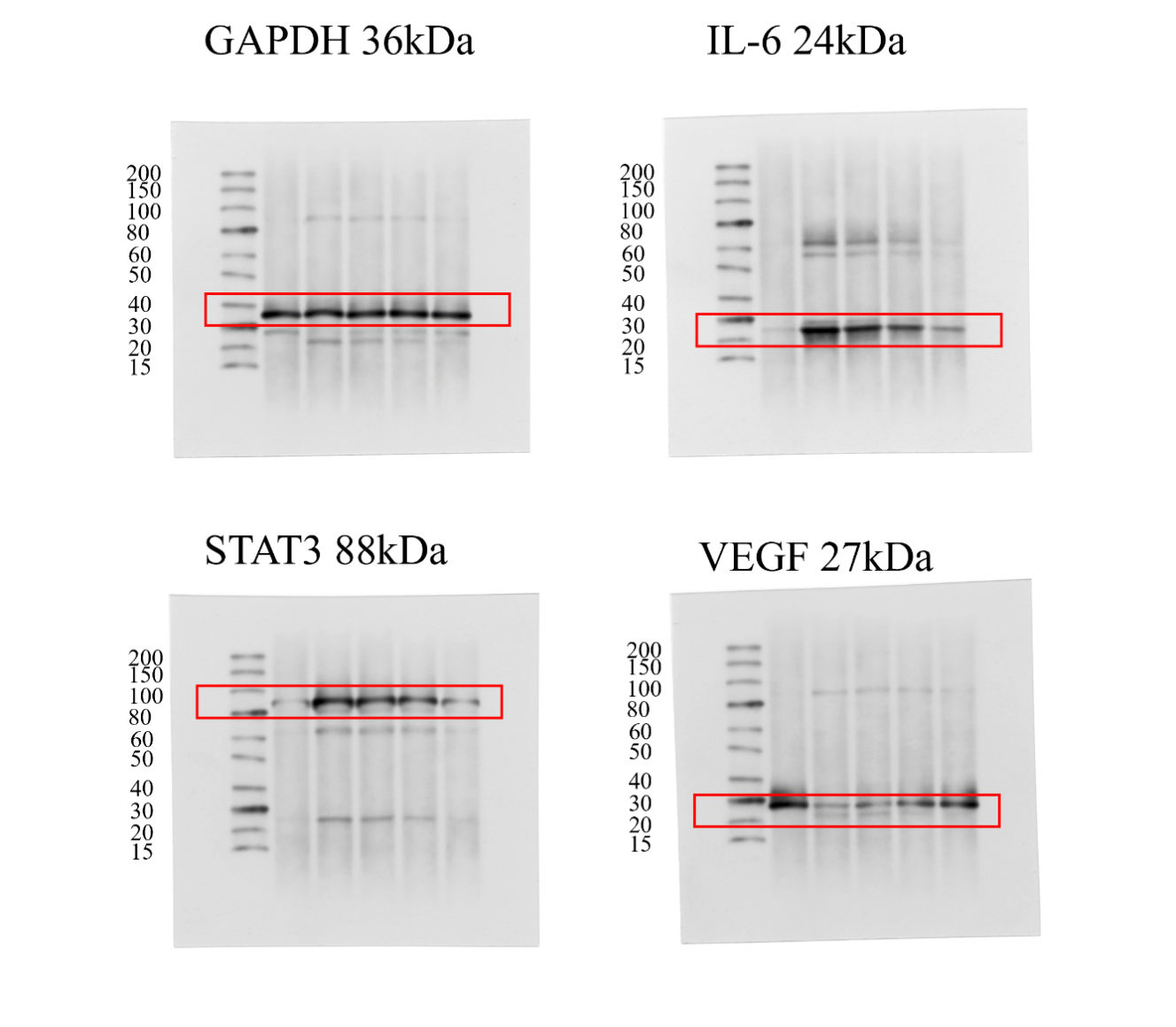


**3. Supplementary Figure 3. Uncropped full-length images of the gel and blot for CD206, iNOS, p-STAT3, STAT3, and GAPDH from the same gel.**

**
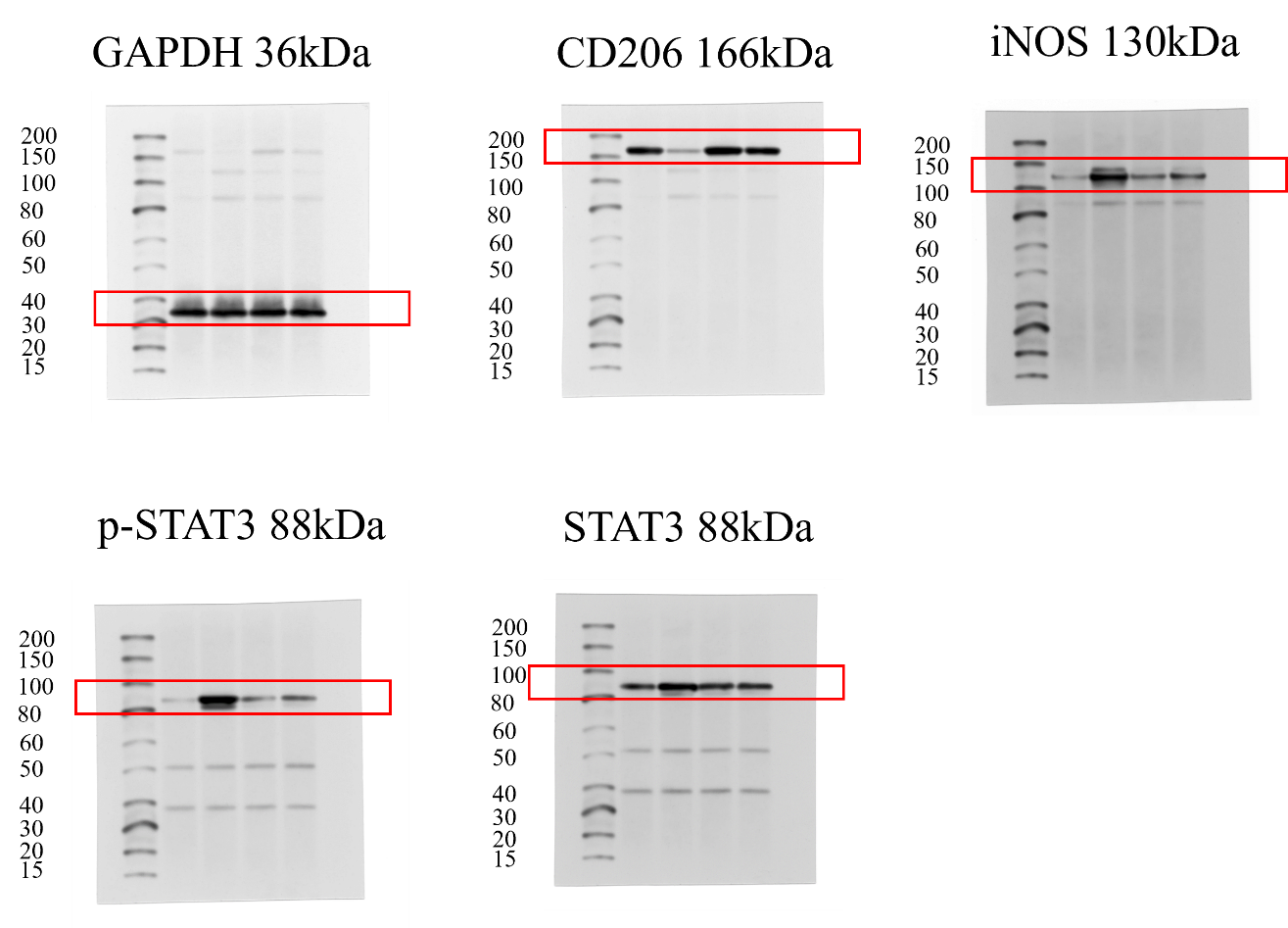
**

Supplementary Tables:

1. **Supplementary Table 1. 134 targets of AD from CCPI.**

| NO. | ID | Target |
| --- | --- | --- |
| 1 | P15090 | FABP4 |
| 2 | P37231 | PPARG |
| 3 | Q07869 | PPARA |
| 4 | P05413 | FABP3 |
| 5 | P23219 | PTGS1 |
| 6 | O14842 | FFAR1 |
| 7 | Q03181 | PPARD |
| 8 | Q01469 | FABP5 |
| 9 | O00519 | FAAH |
| 10 | O14746 | TERT |
| 11 | P07148 | FABP1 |
| 12 | P21554 | CNR1 |
| 13 | O00767 | SCD |
| 14 | P18031 | PTPN1 |
| 15 | P17706 | PTPN2 |
| 16 | P09917 | ALOX5 |
| 17 | P06746 | POLB |
| 18 | O14684 | PTGES |
| 19 | O00748 | CES2 |
| 20 | P48147 | PREP |
| 21 | P51449 | RORC |
| 22 | Q15722 | LTB4R |
| 23 | P29350 | PTPN6 |
| 24 | P18054 | ALOX12 |
| 25 | Q92731 | ESR2 |
| 26 | P28845 | HSD11B1 |
| 27 | Q08499 | PDE4D |
| 28 | O60218 | AKR1B10 |
| 29 | P30304 | CDC25A |
| 30 | P11387 | TOP1 |
| 31 | P43116 | PTGER2 |
| 32 | P43119 | PTGIR |
| 33 | P35228 | NOS2 |
| 34 | P11511 | CYP19A1 |
| 35 | Q13133 | NR1H3 |
| 36 | P10586 | PTPRF |
| 37 | Q8NER1 | TRPV1 |
| 38 | P04150 | NR3C1 |
| 39 | P60033 | CD81 |
| 40 | P24723 | PRKCH |
| 41 | P0DMS8 | ADORA3 |
| 42 | P08185 | SERPINA6 |
| 43 | P04278 | SHBG |
| 44 | P11413 | G6PD |
| 45 | Q16850 | CYP51A1 |
| 46 | P31213 | SRD5A2 |
| 47 | Q06124 | PTPN11 |
| 48 | P24666 | ACP1 |
| 49 | Q9NR63 | CYP26B1 |
| 50 | P35354 | PTGS2 |
| 51 | P04035 | HMGCR |
| 52 | Q5NUL3 | FFAR4 |
| 53 | Q9UHC9 | NPC1L1 |
| 54 | Q99720 | SIGMAR1 |
| 55 | P05093 | CYP17A1 |
| 56 | P04054 | PLA2G1B |
| 57 | P05231 | IL6 |
| 58 | P15104 | GLUL |
| 59 | O43174 | CYP26A1 |
| 60 | P80365 | HSD11B2 |
| 61 | P35408 | PTGER4 |
| 62 | Q13822 | ENPP2 |
| 63 | Q9Y5Y4 | PTGDR2 |
| 64 | P28702 | RXRB |
| 65 | P34995 | PTGER1 |
| 66 | P41595 | HTR2B |
| 67 | Q92753 | RORB |
| 68 | P28482 | MAPK1 |
| 69 | P53985 | SLC16A1 |
| 70 | P30305 | CDC25B |
| 71 | P10275 | AR |
| 72 | P27361 | MAPK3 |
| 73 | Q8TDU6 | GPBAR1 |
| 74 | P03372 | ESR1 |
| 75 | P08235 | NR3C2 |
| 76 | P02753 | RBP4 |
| 77 | P06276 | BCHE |
| 78 | P23946 | CMA1 |
| 79 | P08311 | CTSG |
| 80 | P47712 | PLA2G4A |
| 81 | P06401 | PGR |
| 82 | P11388 | TOP2A |
| 83 | P35398 | RORA |
| 84 | P16050 | ALOX15 |
| 85 | P14416 | DRD2 |
| 86 | P18089 | ADRA2B |
| 87 | Q16539 | MAPK14 |
| 88 | P20292 | ALOX5AP |
| 89 | P12104 | FABP2 |
| 90 | P27815 | PDE4A |
| 91 | Q07343 | PDE4B |
| 92 | P49354 | FNTA |
| 93 | P49356 | FNTB |
| 94 | P49810 | PSEN2 |
| 95 | P49768 | PSEN1 |
| 96 | Q9NZ42 | PSENEN |
| 97 | Q8WW43 | APH1B |
| 98 | Q92542 | NCSTN |
| 99 | Q96BI3 | APH1A |
| 100 | P20701 | ITGAL |
| 101 | P05362 | ICAM1 |
| 102 | P05107 | ITGB2 |
| 103 | Q4U2R8 | SLC22A6 |
| 104 | P00918 | CA2 |
| 105 | P54289 | CACNA2D1 |
| 106 | Q01650 | SLC7A5 |
| 107 | Q9UBS5 | GABBR1 |
| 108 | B2RXH2 | KDM4E |
| 109 | P06241 | FYN |
| 110 | P00533 | EGFR |
| 111 | P25103 | TACR1 |
| 112 | P07101 | TH |
| 113 | Q9H3R0 | KDM4C |
| 114 | P06239 | LCK |
| 115 | P24046 | GABRR1 |
| 116 | P08913 | ADRA2A |
| 117 | P35348 | ADRA1A |
| 118 | Q96IY4 | CPB2 |
| 119 | P15144 | ANPEP |
| 120 | Q07075 | ENPEP |
| 121 | O60341 | KDM1A |
| 122 | P46059 | SLC15A1 |
| 123 | Q04609 | FOLH1 |
| 124 | P15088 | CPA3 |
| 125 | P23975 | SLC6A2 |
| 126 | P31645 | SLC6A4 |
| 127 | Q96RJ0 | TAAR1 |
| 128 | P15086 | CPB1 |
| 129 | P42261 | GRIA1 |
| 130 | O75899 | GABBR2 |
| 131 | P29274 | ADORA2A |
| 132 | P09960 | LTA4H |
| 133 | P41594 | GRM5 |
| 134 | Q9GZT9 | EGLN1 |

1. **Supplementary Table 2. 50 disease targets of AD.**

| NO. | ID | Target |
| --- | --- | --- |
| 1 | Q9NZ42 | PSENEN |
| 2 | Q99720 | SIGMAR1 |
| 3 | Q96BI3 | APH1A |
| 4 | Q92731 | ESR2 |
| 5 | Q92542 | NCSTN |
| 6 | Q8WW43 | APH1B |
| 7 | Q16539 | MAPK14 |
| 8 | Q13133 | NR1H3 |
| 9 | Q08499 | PDE4D |
| 10 | Q07869 | PPARA |
| 11 | Q06124 | PTPN11 |
| 12 | P49810 | PSEN2 |
| 13 | P49768 | PSEN1 |
| 14 | P48147 | PREP |
| 15 | P47712 | PLA2G4A |
| 16 | P42261 | GRIA1 |
| 17 | P37231 | PPARG |
| 18 | P35354 | PTGS2 |
| 19 | P35228 | NOS2 |
| 20 | P31645 | SLC6A4 |
| 21 | P29274 | ADORA2A |
| 22 | P28845 | HSD11B1 |
| 23 | P02753 | RBP4 |
| 24 | P00533 | EGFR |
| 25 | O14746 | TERT |
| 26 | P28482 | MAPK1 |
| 27 | P27815 | PDE4A |
| 28 | P27361 | MAPK3 |
| 29 | P23975 | SLC6A2 |
| 30 | P23219 | PTGS1 |
| 31 | P21554 | CNR1 |
| 32 | P18031 | PTPN1 |
| 33 | P15104 | GLUL |
| 34 | P14416 | DRD2 |
| 35 | P11511 | CYP19A1 |
| 36 | P11413 | G6PD |
| 37 | P10275 | AR |
| 38 | P09917 | ALOX5 |
| 39 | P08311 | CTSG |
| 40 | P08235 | NR3C2 |
| 41 | P07101 | TH |
| 42 | P06276 | BCHE |
| 43 | P06241 | FYN |
| 44 | P05362 | ICAM1 |
| 45 | P05231 | IL6 |
| 46 | P05107 | ITGB2 |
| 47 | P05093 | CYP17A1 |
| 48 | P04150 | NR3C1 |
| 49 | P04035 | HMGCR |
| 50 | P03372 | ESR1 |

1. **Supplementary Table 3.** **Degree information for PPI.**

| NO. | Target | Degree |
| --- | --- | --- |
| 1 | IL6 | 35 |
| 2 | MAPK3 | 23 |
| 3 | PPARG | 22 |
| 4 | EGFR | 20 |
| 5 | MAPK1 | 19 |
| 6 | PPARA | 19 |
| 7 | PTGS2 | 19 |
| 8 | NR3C1 | 18 |
| 9 | MAPK14 | 17 |
| 10 | ESR1 | 17 |
| 11 | TH | 15 |
| 12 | CYP19A1 | 14 |
| 13 | FYN | 14 |
| 14 | CNR1 | 13 |
| 15 | AR | 13 |
| 16 | PSEN1 | 13 |
| 17 | ICAM1 | 12 |
| 18 | PLA2G4A | 12 |
| 19 | PTPN1 | 11 |
| 20 | PTPN11 | 11 |
| 21 | ESR2 | 10 |
| 22 | ALOX5 | 10 |
| 23 | DRD2 | 9 |
| 24 | HSD11B1 | 9 |
| 25 | NOS2 | 9 |
| 26 | ADORA2A | 9 |
| 27 | SLC6A4 | 8 |
| 28 | GRIA1 | 8 |
| 29 | TERT | 8 |
| 30 | CYP17A1 | 8 |
| 31 | NCSTN | 7 |
| 32 | HMGCR | 7 |
| 33 | ITGB2 | 7 |
| 34 | PTGS1 | 7 |
| 35 | NR3C2 | 6 |
| 36 | PSEN2 | 6 |
| 37 | APH1A | 6 |
| 38 | APH1B | 6 |
| 39 | PSENEN | 6 |
| 40 | BCHE | 5 |
| 41 | G6PD | 5 |
| 42 | NR1H3 | 5 |
| 43 | PDE4A | 4 |
| 44 | SIGMAR1 | 4 |
| 45 | SLC6A2 | 3 |
| 46 | RBP4 | 2 |
| 47 | CTSG | 1 |
| 48 | PDE4D | 1 |
| 49 | GLUL | 1 |
| 50 | PREP | 1 |

1. **Supplementary Table 4. 2631 biological processes, cellular components, and molecular functions were enriched by GO.**

| NO. | Category | GO | Description |
| --- | --- | --- | --- |
| 1 | GO Molecular Functions | GO:0033993 | response to lipid |
| 2 | GO Molecular Functions | GO:0014070 | response to organic cyclic compound |
| 3 | GO Molecular Functions | GO:0071396 | cellular response to lipid |
| 4 | GO Molecular Functions | GO:1901700 | response to oxygen-containing compound |
| 5 | GO Molecular Functions | GO:0042493 | response to drug |
| 6 | GO Molecular Functions | GO:1901701 | cellular response to oxygen-containing compound |
| 7 | GO Molecular Functions | GO:0071407 | cellular response to organic cyclic compound |
| 8 | GO Molecular Functions | GO:0010243 | response to organonitrogen compound |
| 9 | GO Molecular Functions | GO:0032101 | regulation of response to external stimulus |
| 10 | GO Molecular Functions | GO:0035690 | cellular response to drug |
| 11 | GO Molecular Functions | GO:0072359 | circulatory system development |
| 12 | GO Molecular Functions | GO:0009719 | response to endogenous stimulus |
| 13 | GO Molecular Functions | GO:0048545 | response to steroid hormone |
| 14 | GO Molecular Functions | GO:1901698 | response to nitrogen compound |
| 15 | GO Molecular Functions | GO:0071363 | cellular response to growth factor stimulus |
| 16 | GO Molecular Functions | GO:0033002 | muscle cell proliferation |
| 17 | GO Molecular Functions | GO:0001503 | ossification |
| 18 | GO Molecular Functions | GO:0071310 | cellular response to organic substance |
| 19 | GO Molecular Functions | GO:0019902 | phosphatase binding |
| 20 | GO Molecular Functions | GO:0070848 | response to growth factor |
| 21 | GO Molecular Functions | GO:0006915 | apoptotic process |
| 22 | GO Molecular Functions | GO:0031328 | positive regulation of cellular biosynthetic process |
| 23 | GO Molecular Functions | GO:0009891 | positive regulation of biosynthetic process |
| 24 | GO Molecular Functions | GO:0012501 | programmed cell death |
| 25 | GO Molecular Functions | GO:0071495 | cellular response to endogenous stimulus |
| 26 | GO Molecular Functions | GO:0019899 | enzyme binding |
| 27 | GO Molecular Functions | GO:0006352 | DNA-templated transcription, initiation |
| 28 | GO Molecular Functions | GO:0031667 | response to nutrient levels |
| 29 | GO Molecular Functions | GO:0070887 | cellular response to chemical stimulus |
| 30 | GO Molecular Functions | GO:0010033 | response to organic substance |
| 31 | GO Molecular Functions | GO:0008219 | cell death |
| 32 | GO Molecular Functions | GO:0007507 | heart development |
| 33 | GO Molecular Functions | GO:0009991 | response to extracellular stimulus |
| 34 | GO Molecular Functions | GO:0018105 | peptidyl-serine phosphorylation |
| 35 | GO Molecular Functions | GO:0050727 | regulation of inflammatory response |
| 36 | GO Molecular Functions | GO:0001890 | placenta development |
| 37 | GO Molecular Functions | GO:0031347 | regulation of defense response |
| 38 | GO Molecular Functions | GO:0004879 | nuclear receptor activity |
| 39 | GO Molecular Functions | GO:0098531 | transcription factor activity, direct ligand regulated sequence-specific DNA binding |
| 40 | GO Molecular Functions | GO:0032496 | response to lipopolysaccharide |
| 41 | GO Molecular Functions | GO:0051384 | response to glucocorticoid |
| 42 | GO Molecular Functions | GO:0009725 | response to hormone |
| 43 | GO Molecular Functions | GO:0018209 | peptidyl-serine modification |
| 44 | GO Molecular Functions | GO:0035295 | tube development |
| 45 | GO Molecular Functions | GO:0045893 | positive regulation of transcription, DNA-templated |
| 46 | GO Molecular Functions | GO:1903508 | positive regulation of nucleic acid-templated transcription |
| 47 | GO Molecular Functions | GO:1902680 | positive regulation of RNA biosynthetic process |
| 48 | GO Molecular Functions | GO:0002237 | response to molecule of bacterial origin |
| 49 | GO Molecular Functions | GO:0010608 | posttranscriptional regulation of gene expression |
| 50 | GO Molecular Functions | GO:0031960 | response to corticosteroid |
| 51 | GO Molecular Functions | GO:0051254 | positive regulation of RNA metabolic process |
| 52 | GO Molecular Functions | GO:0071496 | cellular response to external stimulus |
| 53 | GO Molecular Functions | GO:0080134 | regulation of response to stress |
| 54 | GO Molecular Functions | GO:0060135 | maternal process involved in female pregnancy |
| 55 | GO Molecular Functions | GO:0004707 | MAP kinase activity |
| 56 | GO Molecular Functions | GO:0006631 | fatty acid metabolic process |
| 57 | GO Molecular Functions | GO:0045944 | positive regulation of transcription by RNA polymerase II |
| 58 | GO Molecular Functions | GO:0009653 | anatomical structure morphogenesis |
| 59 | GO Molecular Functions | GO:0009605 | response to external stimulus |
| 60 | GO Molecular Functions | GO:0010557 | positive regulation of macromolecule biosynthetic process |
| 61 | GO Molecular Functions | GO:0071248 | cellular response to metal ion |
| 62 | GO Molecular Functions | GO:0030278 | regulation of ossification |
| 63 | GO Molecular Functions | GO:0009628 | response to abiotic stimulus |
| 64 | GO Molecular Functions | GO:0048608 | reproductive structure development |
| 65 | GO Molecular Functions | GO:0051090 | regulation of DNA-binding transcription factor activity |
| 66 | GO Molecular Functions | GO:0061458 | reproductive system development |
| 67 | GO Molecular Functions | GO:0042592 | homeostatic process |
| 68 | GO Molecular Functions | GO:0045935 | positive regulation of nucleobase-containing compound metabolic process |
| 69 | GO Molecular Functions | GO:0009612 | response to mechanical stimulus |
| 70 | GO Molecular Functions | GO:0048708 | astrocyte differentiation |
| 71 | GO Molecular Functions | GO:0004708 | MAP kinase kinase activity |
| 72 | GO Molecular Functions | GO:0006367 | transcription initiation from RNA polymerase II promoter |
| 73 | GO Molecular Functions | GO:0071241 | cellular response to inorganic substance |
| 74 | GO Molecular Functions | GO:0010001 | glial cell differentiation |
| 75 | GO Molecular Functions | GO:0060965 | negative regulation of gene silencing by miRNA |
| 76 | GO Molecular Functions | GO:0060149 | negative regulation of posttranscriptional gene silencing |
| 77 | GO Molecular Functions | GO:0060967 | negative regulation of gene silencing by RNA |
| 78 | GO Molecular Functions | GO:0009888 | tissue development |
| 79 | GO Molecular Functions | GO:0010628 | positive regulation of gene expression |
| 80 | GO Molecular Functions | GO:0019438 | aromatic compound biosynthetic process |
| 81 | GO Molecular Functions | GO:0010888 | negative regulation of lipid storage |
| 82 | GO Molecular Functions | GO:0006629 | lipid metabolic process |
| 83 | GO Molecular Functions | GO:0009636 | response to toxic substance |
| 84 | GO Molecular Functions | GO:0022414 | reproductive process |
| 85 | GO Molecular Functions | GO:0000003 | reproduction |
| 86 | GO Molecular Functions | GO:0008284 | positive regulation of cell proliferation |
| 87 | GO Molecular Functions | GO:1901362 | organic cyclic compound biosynthetic process |
| 88 | GO Molecular Functions | GO:0006954 | inflammatory response |
| 89 | GO Molecular Functions | GO:0070920 | regulation of production of small RNA involved in gene silencing by RNA |
| 90 | GO Molecular Functions | GO:1903798 | regulation of production of miRNAs involved in gene silencing by miRNA |
| 91 | GO Molecular Functions | GO:0051173 | positive regulation of nitrogen compound metabolic process |
| 92 | GO Molecular Functions | GO:0031669 | cellular response to nutrient levels |
| 93 | GO Molecular Functions | GO:0051239 | regulation of multicellular organismal process |
| 94 | GO Molecular Functions | GO:0042981 | regulation of apoptotic process |
| 95 | GO Molecular Functions | GO:0010035 | response to inorganic substance |
| 96 | GO Molecular Functions | GO:0046697 | decidualization |
| 97 | GO Molecular Functions | GO:0043067 | regulation of programmed cell death |
| 98 | GO Molecular Functions | GO:0003707 | steroid hormone receptor activity |
| 99 | GO Molecular Functions | GO:0042221 | response to chemical |
| 100 | GO Molecular Functions | GO:0042060 | wound healing |
| 101 | GO Molecular Functions | GO:0010604 | positive regulation of macromolecule metabolic process |
| 102 | GO Molecular Functions | GO:0060964 | regulation of gene silencing by miRNA |
| 103 | GO Molecular Functions | GO:0031325 | positive regulation of cellular metabolic process |
| 104 | GO Molecular Functions | GO:0031668 | cellular response to extracellular stimulus |
| 105 | GO Molecular Functions | GO:0042063 | gliogenesis |
| 106 | GO Molecular Functions | GO:0060147 | regulation of posttranscriptional gene silencing |
| 107 | GO Molecular Functions | GO:0060966 | regulation of gene silencing by RNA |
| 108 | GO Molecular Functions | GO:0048511 | rhythmic process |
| 109 | GO Molecular Functions | GO:0042127 | regulation of cell proliferation |
| 110 | GO Molecular Functions | GO:0009887 | animal organ morphogenesis |
| 111 | GO Molecular Functions | GO:0045598 | regulation of fat cell differentiation |
| 112 | GO Molecular Functions | GO:0007568 | aging |
| 113 | GO Molecular Functions | GO:0010941 | regulation of cell death |
| 114 | GO Molecular Functions | GO:0032103 | positive regulation of response to external stimulus |
| 115 | GO Molecular Functions | GO:0044255 | cellular lipid metabolic process |
| 116 | GO Cellular Components | GO:0048646 | anatomical structure formation involved in morphogenesis |
| 117 | GO Cellular Components | GO:0032787 | monocarboxylic acid metabolic process |
| 118 | GO Cellular Components | GO:0035051 | cardiocyte differentiation |
| 119 | GO Cellular Components | GO:0001893 | maternal placenta development |
| 120 | GO Cellular Components | GO:0048513 | animal organ development |
| 121 | GO Cellular Components | GO:0048660 | regulation of smooth muscle cell proliferation |
| 122 | GO Cellular Components | GO:0045923 | positive regulation of fatty acid metabolic process |
| 123 | GO Cellular Components | GO:0032355 | response to estradiol |
| 124 | GO Cellular Components | GO:0051049 | regulation of transport |
| 125 | GO Cellular Components | GO:0010562 | positive regulation of phosphorus metabolic process |
| 126 | GO Cellular Components | GO:0045937 | positive regulation of phosphate metabolic process |
| 127 | GO Cellular Components | GO:0048659 | smooth muscle cell proliferation |
| 128 | GO Cellular Components | GO:0060969 | negative regulation of gene silencing |
| 129 | GO Cellular Components | GO:0003006 | developmental process involved in reproduction |
| 130 | GO Cellular Components | GO:0009893 | positive regulation of metabolic process |
| 131 | GO Cellular Components | GO:0009617 | response to bacterium |
| 132 | GO Cellular Components | GO:0035195 | gene silencing by miRNA |
| 133 | GO Cellular Components | GO:0009611 | response to wounding |
| 134 | GO Cellular Components | GO:0048878 | chemical homeostasis |
| 135 | GO Cellular Components | GO:0060968 | regulation of gene silencing |
| 136 | GO Cellular Components | GO:0071276 | cellular response to cadmium ion |
| 137 | GO Cellular Components | GO:0010038 | response to metal ion |
| 138 | GO Cellular Components | GO:0035194 | posttranscriptional gene silencing by RNA |
| 139 | GO Cellular Components | GO:0042307 | positive regulation of protein import into nucleus |
| 140 | GO Cellular Components | GO:0035265 | organ growth |
| 141 | GO Cellular Components | GO:0090170 | regulation of Golgi inheritance |
| 142 | GO Cellular Components | GO:0034614 | cellular response to reactive oxygen species |
| 143 | GO Cellular Components | GO:0016441 | posttranscriptional gene silencing |
| 144 | GO Cellular Components | GO:0030168 | platelet activation |
| 145 | GO Cellular Components | GO:1904591 | positive regulation of protein import |
| 146 | GO Cellular Components | GO:1902531 | regulation of intracellular signal transduction |
| 147 | GO Cellular Components | GO:0050673 | epithelial cell proliferation |
| 148 | GO Cellular Components | GO:0008283 | cell proliferation |
| 149 | GO Cellular Components | GO:0006952 | defense response |
| 150 | GO Cellular Components | GO:0035094 | response to nicotine |
| 151 | GO Cellular Components | GO:0051704 | multi-organism process |
| 152 | GO Cellular Components | GO:1905953 | negative regulation of lipid localization |
| 153 | GO Cellular Components | GO:0009968 | negative regulation of signal transduction |
| 154 | GO Cellular Components | GO:0002534 | cytokine production involved in inflammatory response |
| 155 | GO Cellular Components | GO:1900015 | regulation of cytokine production involved in inflammatory response |
| 156 | GO Cellular Components | GO:0004712 | protein serine/threonine/tyrosine kinase activity |
| 157 | GO Cellular Components | GO:0070849 | response to epidermal growth factor |
| 158 | GO Cellular Components | GO:1902893 | regulation of pri-miRNA transcription by RNA polymerase II |
| 159 | GO Cellular Components | GO:0010891 | negative regulation of sequestering of triglyceride |
| 160 | GO Cellular Components | GO:0120041 | positive regulation of macrophage proliferation |
| 161 | GO Cellular Components | GO:0035196 | production of miRNAs involved in gene silencing by miRNA |
| 162 | GO Cellular Components | GO:0051972 | regulation of telomerase activity |
| 163 | GO Cellular Components | GO:0061614 | pri-miRNA transcription by RNA polymerase II |
| 164 | GO Cellular Components | GO:0010883 | regulation of lipid storage |
| 165 | GO Biological Processes | GO:0048732 | gland development |
| 166 | GO Biological Processes | GO:0031047 | gene silencing by RNA |
| 167 | GO Biological Processes | GO:2000377 | regulation of reactive oxygen species metabolic process |
| 168 | GO Biological Processes | GO:0007565 | female pregnancy |
| 169 | GO Biological Processes | GO:0071222 | cellular response to lipopolysaccharide |
| 170 | GO Biological Processes | GO:0065008 | regulation of biological quality |
| 171 | GO Biological Processes | GO:0031050 | dsRNA processing |
| 172 | GO Biological Processes | GO:0070918 | production of small RNA involved in gene silencing by RNA |
| 173 | GO Biological Processes | GO:0006979 | response to oxidative stress |
| 174 | GO Biological Processes | GO:0048609 | multicellular organismal reproductive process |
| 175 | GO Biological Processes | GO:2000026 | regulation of multicellular organismal development |
| 176 | GO Biological Processes | GO:0010648 | negative regulation of cell communication |
| 177 | GO Biological Processes | GO:0033554 | cellular response to stress |
| 178 | GO Biological Processes | GO:0023057 | negative regulation of signaling |
| 179 | GO Biological Processes | GO:0051052 | regulation of DNA metabolic process |
| 180 | GO Biological Processes | GO:0000186 | activation of MAPKK activity |
| 181 | GO Biological Processes | GO:0031663 | lipopolysaccharide-mediated signaling pathway |
| 182 | GO Biological Processes | GO:0071219 | cellular response to molecule of bacterial origin |
| 183 | GO Biological Processes | GO:1901654 | response to ketone |
| 184 | GO Biological Processes | GO:0042473 | outer ear morphogenesis |
| 185 | GO Biological Processes | GO:0060440 | trachea formation |
| 186 | GO Biological Processes | GO:0120040 | regulation of macrophage proliferation |
| 187 | GO Biological Processes | GO:0055021 | regulation of cardiac muscle tissue growth |
| 188 | GO Biological Processes | GO:0032504 | multicellular organism reproduction |
| 189 | GO Biological Processes | GO:0031326 | regulation of cellular biosynthetic process |
| 190 | GO Biological Processes | GO:0051525 | NFAT protein binding |
| 191 | GO Biological Processes | GO:0030334 | regulation of cell migration |
| 192 | GO Biological Processes | GO:0007166 | cell surface receptor signaling pathway |
| 193 | GO Biological Processes | GO:0006950 | response to stress |
| 194 | GO Biological Processes | GO:0030154 | cell differentiation |
| 195 | GO Biological Processes | GO:0065009 | regulation of molecular function |
| 196 | GO Biological Processes | GO:0048468 | cell development |
| 197 | GO Biological Processes | GO:0009889 | regulation of biosynthetic process |
| 198 | GO Biological Processes | GO:0034654 | nucleobase-containing compound biosynthetic process |
| 199 | GO Biological Processes | GO:0043069 | negative regulation of programmed cell death |
| 200 | GO Biological Processes | GO:0042306 | regulation of protein import into nucleus |
| 201 | GO Biological Processes | GO:0007623 | circadian rhythm |
| 202 | GO Biological Processes | GO:0045444 | fat cell differentiation |
| 203 | GO Biological Processes | GO:0044706 | multi-multicellular organism process |
| 204 | GO Biological Processes | GO:0018130 | heterocycle biosynthetic process |
| 205 | GO Biological Processes | GO:0045600 | positive regulation of fat cell differentiation |
| 206 | GO Biological Processes | GO:0046824 | positive regulation of nucleocytoplasmic transport |
| 207 | GO Biological Processes | GO:0060420 | regulation of heart growth |
| 208 | GO Biological Processes | GO:1904589 | regulation of protein import |
| 209 | GO Biological Processes | GO:0009966 | regulation of signal transduction |
| 210 | GO Biological Processes | GO:0150076 | neuroinflammatory response |
| 211 | GO Biological Processes | GO:0071216 | cellular response to biotic stimulus |
| 212 | GO Biological Processes | GO:0000302 | response to reactive oxygen species |
| 213 | GO Biological Processes | GO:0009266 | response to temperature stimulus |
| 214 | GO Biological Processes | GO:0050878 | regulation of body fluid levels |
| 215 | GO Biological Processes | GO:0040007 | growth |
| 216 | GO Biological Processes | GO:0031349 | positive regulation of defense response |
| 217 | GO Biological Processes | GO:0008015 | blood circulation |
| 218 | GO Biological Processes | GO:0046686 | response to cadmium ion |
| 219 | GO Biological Processes | GO:2000145 | regulation of cell motility |
| 220 | GO Biological Processes | GO:0044249 | cellular biosynthetic process |
| 221 | GO Biological Processes | GO:0030335 | positive regulation of cell migration |
| 222 | GO Biological Processes | GO:0042594 | response to starvation |
| 223 | GO Biological Processes | GO:0046483 | heterocycle metabolic process |
| 224 | GO Biological Processes | GO:0048869 | cellular developmental process |
| 225 | GO Biological Processes | GO:0002532 | production of molecular mediator involved in inflammatory response |
| 226 | GO Biological Processes | GO:0003013 | circulatory system process |
| 227 | GO Biological Processes | GO:0006725 | cellular aromatic compound metabolic process |
| 228 | GO Biological Processes | GO:1901652 | response to peptide |
| 229 | GO Biological Processes | GO:0009755 | hormone-mediated signaling pathway |
| 230 | GO Biological Processes | GO:1901576 | organic substance biosynthetic process |
| 231 | GO Biological Processes | GO:0051223 | regulation of protein transport |
| 232 | GO Biological Processes | GO:0043410 | positive regulation of MAPK cascade |
| 233 | GO Biological Processes | GO:0051054 | positive regulation of DNA metabolic process |
| 234 | GO Biological Processes | GO:2000147 | positive regulation of cell motility |
| 235 | GO Biological Processes | GO:0060548 | negative regulation of cell death |
| 236 | GO Biological Processes | GO:0071383 | cellular response to steroid hormone stimulus |
| 237 | GO Biological Processes | GO:0014015 | positive regulation of gliogenesis |
| 238 | GO Biological Processes | GO:0009058 | biosynthetic process |
| 239 | GO Biological Processes | GO:0055024 | regulation of cardiac muscle tissue development |
| 240 | GO Biological Processes | GO:0001934 | positive regulation of protein phosphorylation |
| 241 | GO Biological Processes | GO:0010887 | negative regulation of cholesterol storage |
| 242 | GO Biological Processes | GO:0060020 | Bergmann glial cell differentiation |
| 243 | GO Biological Processes | GO:0042325 | regulation of phosphorylation |
| 244 | GO Biological Processes | GO:0032270 | positive regulation of cellular protein metabolic process |
| 245 | GO Biological Processes | GO:0051272 | positive regulation of cellular component movement |
| 246 | GO Biological Processes | GO:0007611 | learning or memory |
| 247 | GO Biological Processes | GO:0043406 | positive regulation of MAP kinase activity |
| 248 | GO Biological Processes | GO:0040012 | regulation of locomotion |
| 249 | GO Biological Processes | GO:0090087 | regulation of peptide transport |
| 250 | GO Biological Processes | GO:0031100 | animal organ regeneration |
| 251 | GO Biological Processes | GO:0043627 | response to estrogen |
| 252 | GO Biological Processes | GO:0055017 | cardiac muscle tissue growth |
| 253 | GO Biological Processes | GO:0071260 | cellular response to mechanical stimulus |
| 254 | GO Biological Processes | GO:0051270 | regulation of cellular component movement |
| 255 | GO Biological Processes | GO:0040017 | positive regulation of locomotion |
| 256 | GO Biological Processes | GO:0007417 | central nervous system development |
| 257 | GO Biological Processes | GO:1901360 | organic cyclic compound metabolic process |
| 258 | GO Biological Processes | GO:0070201 | regulation of establishment of protein localization |
| 259 | GO Biological Processes | GO:0019915 | lipid storage |
| 260 | GO Biological Processes | GO:0051707 | response to other organism |
| 261 | GO Biological Processes | GO:0043207 | response to external biotic stimulus |
| 262 | GO Biological Processes | GO:0007610 | behavior |
| 263 | GO Biological Processes | GO:0033668 | negative regulation by symbiont of host apoptotic process |
| 264 | GO Biological Processes | GO:0007267 | cell-cell signaling |
| 265 | GO Biological Processes | GO:0007167 | enzyme linked receptor protein signaling pathway |
| 266 | GO Biological Processes | GO:0006970 | response to osmotic stress |
| 267 | GO Biological Processes | GO:0071417 | cellular response to organonitrogen compound |
| 268 | GO Biological Processes | GO:0051247 | positive regulation of protein metabolic process |
| 269 | GO Biological Processes | GO:0051172 | negative regulation of nitrogen compound metabolic process |
| 270 | GO Biological Processes | GO:0060419 | heart growth |
| 271 | GO Biological Processes | GO:0072593 | reactive oxygen species metabolic process |
| 272 | GO Biological Processes | GO:0042327 | positive regulation of phosphorylation |
| 273 | GO Biological Processes | GO:0051403 | stress-activated MAPK cascade |
| 274 | GO Biological Processes | GO:0009607 | response to biotic stimulus |
| 275 | GO Biological Processes | GO:0044532 | modulation of apoptotic process in other organism |
| 276 | GO Biological Processes | GO:0052041 | negative regulation by symbiont of host programmed cell death |
| 277 | GO Biological Processes | GO:0052150 | modulation by symbiont of host apoptotic process |
| 278 | GO Biological Processes | GO:0052433 | modulation by organism of apoptotic process in other organism involved in symbiotic interaction |
| 279 | GO Biological Processes | GO:0052490 | negative regulation by organism of programmed cell death in other organism involved in symbiotic interaction |
| 280 | GO Biological Processes | GO:0060439 | trachea morphogenesis |
| 281 | GO Biological Processes | GO:0061517 | macrophage proliferation |
| 282 | GO Biological Processes | GO:1903800 | positive regulation of production of miRNAs involved in gene silencing by miRNA |
| 283 | GO Biological Processes | GO:0048661 | positive regulation of smooth muscle cell proliferation |
| 284 | GO Biological Processes | GO:0006355 | regulation of transcription, DNA-templated |
| 285 | GO Biological Processes | GO:0010646 | regulation of cell communication |
| 286 | GO Biological Processes | GO:1903506 | regulation of nucleic acid-templated transcription |
| 287 | GO Biological Processes | GO:2001141 | regulation of RNA biosynthetic process |
| 288 | GO Biological Processes | GO:0019752 | carboxylic acid metabolic process |
| 289 | GO Biological Processes | GO:1900182 | positive regulation of protein localization to nucleus |
| 290 | GO Biological Processes | GO:0071345 | cellular response to cytokine stimulus |
| 291 | GO Biological Processes | GO:0044093 | positive regulation of molecular function |
| 292 | GO Biological Processes | GO:1902533 | positive regulation of intracellular signal transduction |
| 293 | GO Biological Processes | GO:0023052 | signaling |
| 294 | GO Biological Processes | GO:0023051 | regulation of signaling |
| 295 | GO Biological Processes | GO:0046620 | regulation of organ growth |
| 296 | GO Biological Processes | GO:0009410 | response to xenobiotic stimulus |
| 297 | GO Biological Processes | GO:0048523 | negative regulation of cellular process |
| 298 | GO Biological Processes | GO:0016458 | gene silencing |
| 299 | GO Biological Processes | GO:0032966 | negative regulation of collagen biosynthetic process |
| 300 | GO Biological Processes | GO:0044531 | modulation of programmed cell death in other organism |
| 301 | GO Biological Processes | GO:0052040 | modulation by symbiont of host programmed cell death |
| 302 | GO Biological Processes | GO:0052248 | modulation of programmed cell death in other organism involved in symbiotic interaction |
| 303 | GO Biological Processes | GO:0061307 | cardiac neural crest cell differentiation involved in heart development |
| 304 | GO Biological Processes | GO:0061308 | cardiac neural crest cell development involved in heart development |
| 305 | GO Biological Processes | GO:0072584 | caveolin-mediated endocytosis |
| 306 | GO Biological Processes | GO:0034641 | cellular nitrogen compound metabolic process |
| 307 | GO Biological Processes | GO:0033273 | response to vitamin |
| 308 | GO Biological Processes | GO:0007154 | cell communication |
| 309 | GO Biological Processes | GO:0031098 | stress-activated protein kinase signaling cascade |
| 310 | GO Biological Processes | GO:0050890 | cognition |
| 311 | GO Biological Processes | GO:0071702 | organic substance transport |
| 312 | GO Biological Processes | GO:0048731 | system development |
| 313 | GO Biological Processes | GO:0002690 | positive regulation of leukocyte chemotaxis |
| 314 | GO Biological Processes | GO:0071674 | mononuclear cell migration |
| 315 | GO Biological Processes | GO:0019217 | regulation of fatty acid metabolic process |
| 316 | GO Biological Processes | GO:0034599 | cellular response to oxidative stress |
| 317 | GO Biological Processes | GO:1901655 | cellular response to ketone |
| 318 | GO Biological Processes | GO:0006357 | regulation of transcription by RNA polymerase II |
| 319 | GO Biological Processes | GO:0010713 | negative regulation of collagen metabolic process |
| 320 | GO Biological Processes | GO:0010745 | negative regulation of macrophage derived foam cell differentiation |
| 321 | GO Biological Processes | GO:0040008 | regulation of growth |
| 322 | GO Biological Processes | GO:0051128 | regulation of cellular component organization |
| 323 | GO Biological Processes | GO:0051222 | positive regulation of protein transport |
| 324 | GO Biological Processes | GO:0019220 | regulation of phosphate metabolic process |
| 325 | GO Biological Processes | GO:0051174 | regulation of phosphorus metabolic process |
| 326 | GO Biological Processes | GO:0050793 | regulation of developmental process |
| 327 | GO Biological Processes | GO:1901699 | cellular response to nitrogen compound |
| 328 | GO Biological Processes | GO:0044271 | cellular nitrogen compound biosynthetic process |
| 329 | GO Biological Processes | GO:0010647 | positive regulation of cell communication |
| 330 | GO Biological Processes | GO:0006351 | transcription, DNA-templated |
| 331 | GO Biological Processes | GO:0097659 | nucleic acid-templated transcription |
| 332 | GO Biological Processes | GO:0023056 | positive regulation of signaling |
| 333 | GO Biological Processes | GO:0048585 | negative regulation of response to stimulus |
| 334 | GO Biological Processes | GO:1901566 | organonitrogen compound biosynthetic process |
| 335 | GO Biological Processes | GO:0034612 | response to tumor necrosis factor |
| 336 | GO Biological Processes | GO:0019233 | sensory perception of pain |
| 337 | GO Biological Processes | GO:0010889 | regulation of sequestering of triglyceride |
| 338 | GO Biological Processes | GO:1903799 | negative regulation of production of miRNAs involved in gene silencing by miRNA |
| 339 | GO Biological Processes | GO:0032774 | RNA biosynthetic process |
| 340 | GO Biological Processes | GO:2000379 | positive regulation of reactive oxygen species metabolic process |
| 341 | GO Biological Processes | GO:0001568 | blood vessel development |
| 342 | GO Biological Processes | GO:0034097 | response to cytokine |
| 343 | GO Biological Processes | GO:0043436 | oxoacid metabolic process |
| 344 | GO Biological Processes | GO:0030235 | nitric-oxide synthase regulator activity |
| 345 | GO Biological Processes | GO:0019395 | fatty acid oxidation |
| 346 | GO Biological Processes | GO:0050728 | negative regulation of inflammatory response |
| 347 | GO Biological Processes | GO:0045595 | regulation of cell differentiation |
| 348 | GO Biological Processes | GO:0032147 | activation of protein kinase activity |
| 349 | GO Biological Processes | GO:0033138 | positive regulation of peptidyl-serine phosphorylation |
| 350 | GO Biological Processes | GO:0006082 | organic acid metabolic process |
| 351 | GO Biological Processes | GO:0046321 | positive regulation of fatty acid oxidation |
| 352 | GO Biological Processes | GO:0048308 | organelle inheritance |
| 353 | GO Biological Processes | GO:0048313 | Golgi inheritance |
| 354 | GO Biological Processes | GO:1903351 | cellular response to dopamine |
| 355 | GO Biological Processes | GO:0032879 | regulation of localization |
| 356 | GO Biological Processes | GO:0043405 | regulation of MAP kinase activity |
| 357 | GO Biological Processes | GO:0046822 | regulation of nucleocytoplasmic transport |
| 358 | GO Biological Processes | GO:0051252 | regulation of RNA metabolic process |
| 359 | GO Biological Processes | GO:0071902 | positive regulation of protein serine/threonine kinase activity |
| 360 | GO Biological Processes | GO:0034440 | lipid oxidation |
| 361 | GO Biological Processes | GO:2000278 | regulation of DNA biosynthetic process |
| 362 | GO Biological Processes | GO:0071214 | cellular response to abiotic stimulus |
| 363 | GO Biological Processes | GO:0104004 | cellular response to environmental stimulus |
| 364 | GO Biological Processes | GO:0050776 | regulation of immune response |
| 365 | GO Biological Processes | GO:0001944 | vasculature development |
| 366 | GO Biological Processes | GO:0072358 | cardiovascular system development |
| 367 | GO Biological Processes | GO:0031401 | positive regulation of protein modification process |
| 368 | GO Biological Processes | GO:0006366 | transcription by RNA polymerase II |
| 369 | GO Biological Processes | GO:0032870 | cellular response to hormone stimulus |
| 370 | GO Biological Processes | GO:0010885 | regulation of cholesterol storage |
| 371 | GO Biological Processes | GO:0090336 | positive regulation of brown fat cell differentiation |
| 372 | GO Biological Processes | GO:1903350 | response to dopamine |
| 373 | GO Biological Processes | GO:0043408 | regulation of MAPK cascade |
| 374 | GO Biological Processes | GO:0040011 | locomotion |
| 375 | GO Biological Processes | GO:1904951 | positive regulation of establishment of protein localization |
| 376 | GO Biological Processes | GO:0040029 | regulation of gene expression, epigenetic |
| 377 | GO Biological Processes | GO:0007596 | blood coagulation |
| 378 | GO Biological Processes | GO:1903829 | positive regulation of cellular protein localization |
| 379 | GO Biological Processes | GO:0045596 | negative regulation of cell differentiation |
| 380 | GO Biological Processes | GO:0007599 | hemostasis |
| 381 | GO Biological Processes | GO:0034605 | cellular response to heat |
| 382 | GO Biological Processes | GO:0050817 | coagulation |
| 383 | GO Biological Processes | GO:0030730 | sequestering of triglyceride |
| 384 | GO Biological Processes | GO:1903358 | regulation of Golgi organization |
| 385 | GO Biological Processes | GO:1904355 | positive regulation of telomere capping |
| 386 | GO Biological Processes | GO:0007178 | transmembrane receptor protein serine/threonine kinase signaling pathway |
| 387 | GO Biological Processes | GO:0030522 | intracellular receptor signaling pathway |
| 388 | GO Biological Processes | GO:0002688 | regulation of leukocyte chemotaxis |
| 389 | GO Biological Processes | GO:0018193 | peptidyl-amino acid modification |
| 390 | GO Biological Processes | GO:0010605 | negative regulation of macromolecule metabolic process |
| 391 | GO Biological Processes | GO:0010878 | cholesterol storage |
| 392 | GO Biological Processes | GO:0060252 | positive regulation of glial cell proliferation |
| 393 | GO Biological Processes | GO:2000641 | regulation of early endosome to late endosome transport |
| 394 | GO Biological Processes | GO:0014013 | regulation of gliogenesis |
| 395 | GO Biological Processes | GO:0035556 | intracellular signal transduction |
| 396 | GO Biological Processes | GO:2000112 | regulation of cellular macromolecule biosynthetic process |
| 397 | GO Biological Processes | GO:0010629 | negative regulation of gene expression |
| 398 | GO Biological Processes | GO:0014066 | regulation of phosphatidylinositol 3-kinase signaling |
| 399 | GO Biological Processes | GO:0010556 | regulation of macromolecule biosynthetic process |
| 400 | GO Biological Processes | GO:0016202 | regulation of striated muscle tissue development |
| 401 | GO Biological Processes | GO:0010759 | positive regulation of macrophage chemotaxis |
| 402 | GO Biological Processes | GO:0060438 | trachea development |
| 403 | GO Biological Processes | GO:0051130 | positive regulation of cellular component organization |
| 404 | GO Biological Processes | GO:1901861 | regulation of muscle tissue development |
| 405 | GO Biological Processes | GO:0035270 | endocrine system development |
| 406 | GO Biological Processes | GO:0048634 | regulation of muscle organ development |
| 407 | GO Biological Processes | GO:0045727 | positive regulation of translation |
| 408 | GO Biological Processes | GO:0007275 | multicellular organism development |
| 409 | GO Biological Processes | GO:0070482 | response to oxygen levels |
| 410 | GO Biological Processes | GO:0008134 | transcription factor binding |
| 411 | GO Biological Processes | GO:0019901 | protein kinase binding |
| 412 | GO Biological Processes | GO:0048522 | positive regulation of cellular process |
| 413 | GO Biological Processes | GO:0010042 | response to manganese ion |
| 414 | GO Biological Processes | GO:0031281 | positive regulation of cyclase activity |
| 415 | GO Biological Processes | GO:1905208 | negative regulation of cardiocyte differentiation |
| 416 | GO Biological Processes | GO:0019219 | regulation of nucleobase-containing compound metabolic process |
| 417 | GO Biological Processes | GO:1900180 | regulation of protein localization to nucleus |
| 418 | GO Biological Processes | GO:0051094 | positive regulation of developmental process |
| 419 | GO Biological Processes | GO:0031348 | negative regulation of defense response |
| 420 | GO Biological Processes | GO:0048143 | astrocyte activation |
| 421 | GO Biological Processes | GO:0002687 | positive regulation of leukocyte migration |
| 422 | GO Biological Processes | GO:0043085 | positive regulation of catalytic activity |
| 423 | GO Biological Processes | GO:0033135 | regulation of peptidyl-serine phosphorylation |
| 424 | GO Biological Processes | GO:0050921 | positive regulation of chemotaxis |
| 425 | GO Biological Processes | GO:0033036 | macromolecule localization |
| 426 | GO Biological Processes | GO:0048519 | negative regulation of biological process |
| 427 | GO Biological Processes | GO:0050729 | positive regulation of inflammatory response |
| 428 | GO Biological Processes | GO:0044281 | small molecule metabolic process |
| 429 | GO Biological Processes | GO:0038083 | peptidyl-tyrosine autophosphorylation |
| 430 | GO Biological Processes | GO:0090335 | regulation of brown fat cell differentiation |
| 431 | GO Biological Processes | GO:0035239 | tube morphogenesis |
| 432 | GO Biological Processes | GO:0071705 | nitrogen compound transport |
| 433 | GO Biological Processes | GO:0070665 | positive regulation of leukocyte proliferation |
| 434 | GO Biological Processes | GO:0003008 | system process |
| 435 | GO Biological Processes | GO:0051253 | negative regulation of RNA metabolic process |
| 436 | GO Biological Processes | GO:0045834 | positive regulation of lipid metabolic process |
| 437 | GO Biological Processes | GO:0009892 | negative regulation of metabolic process |
| 438 | GO Biological Processes | GO:0060045 | positive regulation of cardiac muscle cell proliferation |
| 439 | GO Biological Processes | GO:0001932 | regulation of protein phosphorylation |
| 440 | GO Biological Processes | GO:0055114 | oxidation-reduction process |
| 441 | GO Biological Processes | GO:0051716 | cellular response to stimulus |
| 442 | GO Biological Processes | GO:0062013 | positive regulation of small molecule metabolic process |
| 443 | GO Biological Processes | GO:0006606 | protein import into nucleus |
| 444 | GO Biological Processes | GO:0000187 | activation of MAPK activity |
| 445 | GO Biological Processes | GO:0002673 | regulation of acute inflammatory response |
| 446 | GO Biological Processes | GO:0014065 | phosphatidylinositol 3-kinase signaling |
| 447 | GO Biological Processes | GO:0032386 | regulation of intracellular transport |
| 448 | GO Biological Processes | GO:0009408 | response to heat |
| 449 | GO Biological Processes | GO:0001963 | synaptic transmission, dopaminergic |
| 450 | GO Biological Processes | GO:0030878 | thyroid gland development |
| 451 | GO Biological Processes | GO:1904353 | regulation of telomere capping |
| 452 | GO Biological Processes | GO:0090316 | positive regulation of intracellular protein transport |
| 453 | GO Biological Processes | GO:0032880 | regulation of protein localization |
| 454 | GO Biological Processes | GO:1905952 | regulation of lipid localization |
| 455 | GO Biological Processes | GO:0010575 | positive regulation of vascular endothelial growth factor production |
| 456 | GO Biological Processes | GO:1905523 | positive regulation of macrophage migration |
| 457 | GO Biological Processes | GO:0043434 | response to peptide hormone |
| 458 | GO Biological Processes | GO:0060249 | anatomical structure homeostasis |
| 459 | GO Biological Processes | GO:0034250 | positive regulation of cellular amide metabolic process |
| 460 | GO Biological Processes | GO:0048583 | regulation of response to stimulus |
| 461 | GO Biological Processes | GO:0009314 | response to radiation |
| 462 | GO Biological Processes | GO:0010758 | regulation of macrophage chemotaxis |
| 463 | GO Biological Processes | GO:0033598 | mammary gland epithelial cell proliferation |
| 464 | GO Biological Processes | GO:0035902 | response to immobilization stress |
| 465 | GO Biological Processes | GO:1903649 | regulation of cytoplasmic transport |
| 466 | GO Biological Processes | GO:2000637 | positive regulation of gene silencing by miRNA |
| 467 | GO Biological Processes | GO:0019900 | kinase binding |
| 468 | GO Biological Processes | GO:0042802 | identical protein binding |
| 469 | GO Biological Processes | GO:0016477 | cell migration |
| 470 | GO Biological Processes | GO:0050806 | positive regulation of synaptic transmission |
| 471 | GO Biological Processes | GO:0002675 | positive regulation of acute inflammatory response |
| 472 | GO Biological Processes | GO:0060148 | positive regulation of posttranscriptional gene silencing |
| 473 | GO Biological Processes | GO:0051170 | import into nucleus |
| 474 | GO Biological Processes | GO:0006139 | nucleobase-containing compound metabolic process |
| 475 | GO Biological Processes | GO:0048699 | generation of neurons |
| 476 | GO Biological Processes | GO:0051050 | positive regulation of transport |
| 477 | GO Biological Processes | GO:0071549 | cellular response to dexamethasone stimulus |
| 478 | GO Biological Processes | GO:0000165 | MAPK cascade |
| 479 | GO Biological Processes | GO:0023014 | signal transduction by protein phosphorylation |
| 480 | GO Biological Processes | GO:0048856 | anatomical structure development |
| 481 | GO Biological Processes | GO:0043401 | steroid hormone mediated signaling pathway |
| 482 | GO Biological Processes | GO:0060341 | regulation of cellular localization |
| 483 | GO Biological Processes | GO:0030324 | lung development |
| 484 | GO Biological Processes | GO:0045934 | negative regulation of nucleobase-containing compound metabolic process |
| 485 | GO Biological Processes | GO:0010574 | regulation of vascular endothelial growth factor production |
| 486 | GO Biological Processes | GO:0010743 | regulation of macrophage derived foam cell differentiation |
| 487 | GO Biological Processes | GO:0051171 | regulation of nitrogen compound metabolic process |
| 488 | GO Biological Processes | GO:0019725 | cellular homeostasis |
| 489 | GO Biological Processes | GO:0030323 | respiratory tube development |
| 490 | GO Biological Processes | GO:0051968 | positive regulation of synaptic transmission, glutamatergic |
| 491 | GO Biological Processes | GO:0055023 | positive regulation of cardiac muscle tissue growth |
| 492 | GO Biological Processes | GO:0048534 | hematopoietic or lymphoid organ development |
| 493 | GO Biological Processes | GO:0010565 | regulation of cellular ketone metabolic process |
| 494 | GO Biological Processes | GO:0008217 | regulation of blood pressure |
| 495 | GO Biological Processes | GO:0051093 | negative regulation of developmental process |
| 496 | GO Biological Processes | GO:0046320 | regulation of fatty acid oxidation |
| 497 | GO Biological Processes | GO:0060251 | regulation of glial cell proliferation |
| 498 | GO Biological Processes | GO:0150077 | regulation of neuroinflammatory response |
| 499 | GO Biological Processes | GO:0071900 | regulation of protein serine/threonine kinase activity |
| 500 | GO Biological Processes | GO:0009267 | cellular response to starvation |
| 501 | GO Biological Processes | GO:0050790 | regulation of catalytic activity |
| 502 | GO Biological Processes | GO:0048015 | phosphatidylinositol-mediated signaling |
| 503 | GO Biological Processes | GO:0032212 | positive regulation of telomere maintenance via telomerase |
| 504 | GO Biological Processes | GO:0045907 | positive regulation of vasoconstriction |
| 505 | GO Biological Processes | GO:0016310 | phosphorylation |
| 506 | GO Biological Processes | GO:0001525 | angiogenesis |
| 507 | GO Biological Processes | GO:0045597 | positive regulation of cell differentiation |
| 508 | GO Biological Processes | GO:0048017 | inositol lipid-mediated signaling |
| 509 | GO Biological Processes | GO:0010573 | vascular endothelial growth factor production |
| 510 | GO Biological Processes | GO:0035633 | maintenance of permeability of blood-brain barrier |
| 511 | GO Biological Processes | GO:0007165 | signal transduction |
| 512 | GO Biological Processes | GO:0032102 | negative regulation of response to external stimulus |
| 513 | GO Biological Processes | GO:0051338 | regulation of transferase activity |
| 514 | GO Biological Processes | GO:0009967 | positive regulation of signal transduction |
| 515 | GO Biological Processes | GO:0022008 | neurogenesis |
| 516 | GO Biological Processes | GO:0048584 | positive regulation of response to stimulus |
| 517 | GO Biological Processes | GO:0010468 | regulation of gene expression |
| 518 | GO Biological Processes | GO:0010742 | macrophage derived foam cell differentiation |
| 519 | GO Biological Processes | GO:0051973 | positive regulation of telomerase activity |
| 520 | GO Biological Processes | GO:0060421 | positive regulation of heart growth |
| 521 | GO Biological Processes | GO:0071868 | cellular response to monoamine stimulus |
| 522 | GO Biological Processes | GO:0071870 | cellular response to catecholamine stimulus |
| 523 | GO Biological Processes | GO:0090077 | foam cell differentiation |
| 524 | GO Biological Processes | GO:0031099 | regeneration |
| 525 | GO Biological Processes | GO:0080090 | regulation of primary metabolic process |
| 526 | GO Biological Processes | GO:0001085 | RNA polymerase II transcription factor binding |
| 527 | GO Biological Processes | GO:0071897 | DNA biosynthetic process |
| 528 | GO Biological Processes | GO:0045860 | positive regulation of protein kinase activity |
| 529 | GO Biological Processes | GO:0002520 | immune system development |
| 530 | GO Biological Processes | GO:1902532 | negative regulation of intracellular signal transduction |
| 531 | GO Biological Processes | GO:0016070 | RNA metabolic process |
| 532 | GO Biological Processes | GO:0005901 | caveola |
| 533 | GO Biological Processes | GO:0005788 | endoplasmic reticulum lumen |
| 534 | GO Biological Processes | GO:0045121 | membrane raft |
| 535 | GO Biological Processes | GO:0098857 | membrane microdomain |
| 536 | GO Biological Processes | GO:0044853 | plasma membrane raft |
| 537 | GO Biological Processes | GO:0098589 | membrane region |
| 538 | GO Biological Processes | GO:0031143 | pseudopodium |
| 539 | GO Biological Processes | GO:0031974 | membrane-enclosed lumen |
| 540 | GO Biological Processes | GO:0043233 | organelle lumen |
| 541 | GO Biological Processes | GO:0070013 | intracellular organelle lumen |
| 542 | GO Biological Processes | GO:0097421 | liver regeneration |
| 543 | GO Biological Processes | GO:1904358 | positive regulation of telomere maintenance via telomere lengthening |
| 544 | GO Biological Processes | GO:0019903 | protein phosphatase binding |
| 545 | GO Biological Processes | GO:0004674 | protein serine/threonine kinase activity |
| 546 | GO Biological Processes | GO:1901363 | heterocyclic compound binding |
| 547 | GO Biological Processes | GO:0060541 | respiratory system development |
| 548 | GO Biological Processes | GO:0032965 | regulation of collagen biosynthetic process |
| 549 | GO Biological Processes | GO:0044068 | modulation by symbiont of host cellular process |
| 550 | GO Biological Processes | GO:0071392 | cellular response to estradiol stimulus |
| 551 | GO Biological Processes | GO:0002685 | regulation of leukocyte migration |
| 552 | GO Biological Processes | GO:0001223 | transcription coactivator binding |
| 553 | GO Biological Processes | GO:0097159 | organic cyclic compound binding |
| 554 | GO Biological Processes | GO:0048870 | cell motility |
| 555 | GO Biological Processes | GO:0051674 | localization of cell |
| 556 | GO Biological Processes | GO:0044703 | multi-organism reproductive process |
| 557 | GO Biological Processes | GO:0045022 | early endosome to late endosome transport |
| 558 | GO Biological Processes | GO:0009894 | regulation of catabolic process |
| 559 | GO Biological Processes | GO:0031323 | regulation of cellular metabolic process |
| 560 | GO Biological Processes | GO:0048518 | positive regulation of biological process |
| 561 | GO Biological Processes | GO:0060255 | regulation of macromolecule metabolic process |
| 562 | GO Biological Processes | GO:0014002 | astrocyte development |
| 563 | GO Biological Processes | GO:0048246 | macrophage chemotaxis |
| 564 | GO Biological Processes | GO:0055025 | positive regulation of cardiac muscle tissue development |
| 565 | GO Biological Processes | GO:0060043 | regulation of cardiac muscle cell proliferation |
| 566 | GO Biological Processes | GO:0071548 | response to dexamethasone |
| 567 | GO Biological Processes | GO:0071867 | response to monoamine |
| 568 | GO Biological Processes | GO:0071869 | response to catecholamine |
| 569 | GO Biological Processes | GO:1902895 | positive regulation of pri-miRNA transcription by RNA polymerase II |
| 570 | GO Biological Processes | GO:0048738 | cardiac muscle tissue development |
| 571 | GO Biological Processes | GO:0097529 | myeloid leukocyte migration |
| 572 | GO Biological Processes | GO:0001649 | osteoblast differentiation |
| 573 | GO Biological Processes | GO:0007423 | sensory organ development |
| 574 | GO Biological Processes | GO:1905521 | regulation of macrophage migration |
| 575 | GO Biological Processes | GO:0002526 | acute inflammatory response |
| 576 | GO Biological Processes | GO:1903827 | regulation of cellular protein localization |
| 577 | GO Biological Processes | GO:0032502 | developmental process |
| 578 | GO Biological Processes | GO:0050920 | regulation of chemotaxis |
| 579 | GO Biological Processes | GO:0022603 | regulation of anatomical structure morphogenesis |
| 580 | GO Biological Processes | GO:0098927 | vesicle-mediated transport between endosomal compartments |
| 581 | GO Biological Processes | GO:0006259 | DNA metabolic process |
| 582 | GO Biological Processes | GO:0007584 | response to nutrient |
| 583 | GO Biological Processes | GO:0010712 | regulation of collagen metabolic process |
| 584 | GO Biological Processes | GO:0030595 | leukocyte chemotaxis |
| 585 | GO Biological Processes | GO:0032388 | positive regulation of intracellular transport |
| 586 | GO Biological Processes | GO:0048762 | mesenchymal cell differentiation |
| 587 | GO Biological Processes | GO:0002682 | regulation of immune system process |
| 588 | GO Biological Processes | GO:0015031 | protein transport |
| 589 | GO Biological Processes | GO:0017038 | protein import |
| 590 | GO Biological Processes | GO:0032268 | regulation of cellular protein metabolic process |
| 591 | GO Biological Processes | GO:0032872 | regulation of stress-activated MAPK cascade |
| 592 | GO Biological Processes | GO:0051240 | positive regulation of multicellular organismal process |
| 593 | GO Biological Processes | GO:0031324 | negative regulation of cellular metabolic process |
| 594 | GO Biological Processes | GO:0044057 | regulation of system process |
| 595 | GO Biological Processes | GO:0070302 | regulation of stress-activated protein kinase signaling cascade |
| 596 | GO Biological Processes | GO:0034645 | cellular macromolecule biosynthetic process |
| 597 | GO Biological Processes | GO:0051896 | regulation of protein kinase B signaling |
| 598 | GO Biological Processes | GO:0010863 | positive regulation of phospholipase C activity |
| 599 | GO Biological Processes | GO:0045124 | regulation of bone resorption |
| 600 | GO Biological Processes | GO:0033674 | positive regulation of kinase activity |
| 601 | GO Biological Processes | GO:0048514 | blood vessel morphogenesis |
| 602 | GO Biological Processes | GO:0015833 | peptide transport |
| 603 | GO Biological Processes | GO:0031279 | regulation of cyclase activity |
| 604 | GO Biological Processes | GO:0032964 | collagen biosynthetic process |
| 605 | GO Biological Processes | GO:0046622 | positive regulation of organ growth |
| 606 | GO Biological Processes | GO:0060324 | face development |
| 607 | GO Biological Processes | GO:0033157 | regulation of intracellular protein transport |
| 608 | GO Biological Processes | GO:0008104 | protein localization |
| 609 | GO Biological Processes | GO:0048666 | neuron development |
| 610 | GO Biological Processes | GO:0044092 | negative regulation of molecular function |
| 611 | GO Biological Processes | GO:0009059 | macromolecule biosynthetic process |
| 612 | GO Biological Processes | GO:0003690 | double-stranded DNA binding |
| 613 | GO Biological Processes | GO:0048538 | thymus development |
| 614 | GO Biological Processes | GO:1900274 | regulation of phospholipase C activity |
| 615 | GO Biological Processes | GO:1905207 | regulation of cardiocyte differentiation |
| 616 | GO Biological Processes | GO:0042593 | glucose homeostasis |
| 617 | GO Biological Processes | GO:0033500 | carbohydrate homeostasis |
| 618 | GO Biological Processes | GO:0051701 | interaction with host |
| 619 | GO Biological Processes | GO:0031399 | regulation of protein modification process |
| 620 | GO Biological Processes | GO:0070663 | regulation of leukocyte proliferation |
| 621 | GO Biological Processes | GO:0097237 | cellular response to toxic substance |
| 622 | GO Biological Processes | GO:0060038 | cardiac muscle cell proliferation |
| 623 | GO Biological Processes | GO:0043167 | ion binding |
| 624 | GO Biological Processes | GO:0006810 | transport |
| 625 | GO Biological Processes | GO:0042886 | amide transport |
| 626 | GO Biological Processes | GO:0006953 | acute-phase response |
| 627 | GO Biological Processes | GO:0043330 | response to exogenous dsRNA |
| 628 | GO Biological Processes | GO:0060425 | lung morphogenesis |
| 629 | GO Biological Processes | GO:0071622 | regulation of granulocyte chemotaxis |
| 630 | GO Biological Processes | GO:0001103 | RNA polymerase II repressing transcription factor binding |
| 631 | GO Biological Processes | GO:0048589 | developmental growth |
| 632 | GO Biological Processes | GO:0008144 | drug binding |
| 633 | GO Biological Processes | GO:0097305 | response to alcohol |
| 634 | GO Biological Processes | GO:0014009 | glial cell proliferation |
| 635 | GO Biological Processes | GO:0050873 | brown fat cell differentiation |
| 636 | GO Biological Processes | GO:0071675 | regulation of mononuclear cell migration |
| 637 | GO Biological Processes | GO:0007049 | cell cycle |
| 638 | GO Biological Processes | GO:0032206 | positive regulation of telomere maintenance |
| 639 | GO Biological Processes | GO:0061900 | glial cell activation |
| 640 | GO Biological Processes | GO:0045927 | positive regulation of growth |
| 641 | GO Biological Processes | GO:0043491 | protein kinase B signaling |
| 642 | GO Biological Processes | GO:0051179 | localization |
| 643 | GO Biological Processes | GO:0006935 | chemotaxis |
| 644 | GO Biological Processes | GO:0046850 | regulation of bone remodeling |
| 645 | GO Biological Processes | GO:0042330 | taxis |
| 646 | GO Biological Processes | GO:0045184 | establishment of protein localization |
| 647 | GO Biological Processes | GO:0042180 | cellular ketone metabolic process |
| 648 | GO Biological Processes | GO:0090596 | sensory organ morphogenesis |
| 649 | GO Biological Processes | GO:0032210 | regulation of telomere maintenance via telomerase |
| 650 | GO Biological Processes | GO:0048863 | stem cell differentiation |
| 651 | GO Biological Processes | GO:0002684 | positive regulation of immune system process |
| 652 | GO Biological Processes | GO:0016233 | telomere capping |
| 653 | GO Biological Processes | GO:1905517 | macrophage migration |
| 654 | GO Biological Processes | GO:0030258 | lipid modification |
| 655 | GO Biological Processes | GO:0051091 | positive regulation of DNA-binding transcription factor activity |
| 656 | GO Biological Processes | GO:0051234 | establishment of localization |
| 657 | GO Biological Processes | GO:0043331 | response to dsRNA |
| 658 | GO Biological Processes | GO:0051241 | negative regulation of multicellular organismal process |
| 659 | GO Biological Processes | GO:0006412 | translation |
| 660 | GO Biological Processes | GO:0071385 | cellular response to glucocorticoid stimulus |
| 661 | GO Biological Processes | GO:0051246 | regulation of protein metabolic process |
| 662 | GO Biological Processes | GO:0090304 | nucleic acid metabolic process |
| 663 | GO Biological Processes | GO:0046914 | transition metal ion binding |
| 664 | GO Biological Processes | GO:0004672 | protein kinase activity |
| 665 | GO Biological Processes | GO:0001784 | phosphotyrosine residue binding |
| 666 | GO Biological Processes | GO:0030331 | estrogen receptor binding |
| 667 | GO Biological Processes | GO:0051347 | positive regulation of transferase activity |
| 668 | GO Biological Processes | GO:0006468 | protein phosphorylation |
| 669 | GO Biological Processes | GO:1903522 | regulation of blood circulation |
| 670 | GO Biological Processes | GO:0019222 | regulation of metabolic process |
| 671 | GO Biological Processes | GO:0060485 | mesenchyme development |
| 672 | GO Biological Processes | GO:0006585 | dopamine biosynthetic process from tyrosine |
| 673 | GO Biological Processes | GO:0010335 | response to non-ionic osmotic stress |
| 674 | GO Biological Processes | GO:0043402 | glucocorticoid mediated signaling pathway |
| 675 | GO Biological Processes | GO:0071471 | cellular response to non-ionic osmotic stress |
| 676 | GO Biological Processes | GO:2000230 | negative regulation of pancreatic stellate cell proliferation |
| 677 | GO Biological Processes | GO:2000656 | regulation of apolipoprotein binding |
| 678 | GO Biological Processes | GO:2000657 | negative regulation of apolipoprotein binding |
| 679 | GO Biological Processes | GO:0019369 | arachidonic acid metabolic process |
| 680 | GO Biological Processes | GO:0043043 | peptide biosynthetic process |
| 681 | GO Biological Processes | GO:0010518 | positive regulation of phospholipase activity |
| 682 | GO Biological Processes | GO:0071384 | cellular response to corticosteroid stimulus |
| 683 | GO Biological Processes | GO:0034504 | protein localization to nucleus |
| 684 | GO Biological Processes | GO:0019229 | regulation of vasoconstriction |
| 685 | GO Biological Processes | GO:0044003 | modification by symbiont of host morphology or physiology |
| 686 | GO Biological Processes | GO:1904356 | regulation of telomere maintenance via telomere lengthening |
| 687 | GO Biological Processes | GO:2000272 | negative regulation of signaling receptor activity |
| 688 | GO Biological Processes | GO:0007268 | chemical synaptic transmission |
| 689 | GO Biological Processes | GO:0098916 | anterograde trans-synaptic signaling |
| 690 | GO Biological Processes | GO:0048562 | embryonic organ morphogenesis |
| 691 | GO Biological Processes | GO:0045844 | positive regulation of striated muscle tissue development |
| 692 | GO Biological Processes | GO:0048636 | positive regulation of muscle organ development |
| 693 | GO Biological Processes | GO:0071356 | cellular response to tumor necrosis factor |
| 694 | GO Biological Processes | GO:0099537 | trans-synaptic signaling |
| 695 | GO Biological Processes | GO:0001960 | negative regulation of cytokine-mediated signaling pathway |
| 696 | GO Biological Processes | GO:0014855 | striated muscle cell proliferation |
| 697 | GO Biological Processes | GO:0048645 | animal organ formation |
| 698 | GO Biological Processes | GO:1901863 | positive regulation of muscle tissue development |
| 699 | GO Biological Processes | GO:0051641 | cellular localization |
| 700 | GO Biological Processes | GO:0060326 | cell chemotaxis |
| 701 | GO Biological Processes | GO:0007169 | transmembrane receptor protein tyrosine kinase signaling pathway |
| 702 | GO Biological Processes | GO:0099536 | synaptic signaling |
| 703 | GO Biological Processes | GO:0045765 | regulation of angiogenesis |
| 704 | GO Biological Processes | GO:0055080 | cation homeostasis |
| 705 | GO Biological Processes | GO:0070371 | ERK1 and ERK2 cascade |
| 706 | GO Biological Processes | GO:0045453 | bone resorption |
| 707 | GO Biological Processes | GO:0060761 | negative regulation of response to cytokine stimulus |
| 708 | GO Biological Processes | GO:0098586 | cellular response to virus |
| 709 | GO Biological Processes | GO:2000573 | positive regulation of DNA biosynthetic process |
| 710 | GO Biological Processes | GO:1901564 | organonitrogen compound metabolic process |
| 711 | GO Biological Processes | GO:0050896 | response to stimulus |
| 712 | GO Biological Processes | GO:0042698 | ovulation cycle |
| 713 | GO Biological Processes | GO:0061180 | mammary gland epithelium development |
| 714 | GO Biological Processes | GO:0080135 | regulation of cellular response to stress |
| 715 | GO Biological Processes | GO:0098771 | inorganic ion homeostasis |
| 716 | GO Biological Processes | GO:0051966 | regulation of synaptic transmission, glutamatergic |
| 717 | GO Biological Processes | GO:0001817 | regulation of cytokine production |
| 718 | GO Biological Processes | GO:0070661 | leukocyte proliferation |
| 719 | GO Biological Processes | GO:0001816 | cytokine production |
| 720 | GO Biological Processes | GO:0048638 | regulation of developmental growth |
| 721 | GO Biological Processes | GO:0010517 | regulation of phospholipase activity |
| 722 | GO Biological Processes | GO:0031670 | cellular response to nutrient |
| 723 | GO Biological Processes | GO:0045685 | regulation of glial cell differentiation |
| 724 | GO Biological Processes | GO:0060193 | positive regulation of lipase activity |
| 725 | GO Biological Processes | GO:1903524 | positive regulation of blood circulation |
| 726 | GO Biological Processes | GO:0009416 | response to light stimulus |
| 727 | GO Biological Processes | GO:0009895 | negative regulation of catabolic process |
| 728 | GO Biological Processes | GO:0051235 | maintenance of location |
| 729 | GO Biological Processes | GO:0007004 | telomere maintenance via telomerase |
| 730 | GO Biological Processes | GO:0006928 | movement of cell or subcellular component |
| 731 | GO Biological Processes | GO:0030182 | neuron differentiation |
| 732 | GO Biological Processes | GO:0006278 | RNA-dependent DNA biosynthetic process |
| 733 | GO Biological Processes | GO:0046677 | response to antibiotic |
| 734 | GO Biological Processes | GO:0045309 | protein phosphorylated amino acid binding |
| 735 | GO Biological Processes | GO:0045859 | regulation of protein kinase activity |
| 736 | GO Biological Processes | GO:0010467 | gene expression |
| 737 | GO Biological Processes | GO:0050877 | nervous system process |
| 738 | GO Biological Processes | GO:0014823 | response to activity |
| 739 | GO Biological Processes | GO:0060260 | regulation of transcription initiation from RNA polymerase II promoter |
| 740 | GO Biological Processes | GO:0071242 | cellular response to ammonium ion |
| 741 | GO Biological Processes | GO:0044428 | nuclear part |
| 742 | GO Biological Processes | GO:0016773 | phosphotransferase activity, alcohol group as acceptor |
| 743 | GO Biological Processes | GO:1901342 | regulation of vasculature development |
| 744 | GO Biological Processes | GO:0034103 | regulation of tissue remodeling |
| 745 | GO Biological Processes | GO:1900034 | regulation of cellular response to heat |
| 746 | GO Biological Processes | GO:0019221 | cytokine-mediated signaling pathway |
| 747 | GO Biological Processes | GO:0034198 | cellular response to amino acid starvation |
| 748 | GO Biological Processes | GO:0014032 | neural crest cell development |
| 749 | GO Biological Processes | GO:0042310 | vasoconstriction |
| 750 | GO Biological Processes | GO:0048145 | regulation of fibroblast proliferation |
| 751 | GO Biological Processes | GO:0097756 | negative regulation of blood vessel diameter |
| 752 | GO Biological Processes | GO:0050777 | negative regulation of immune response |
| 753 | GO Biological Processes | GO:0032204 | regulation of telomere maintenance |
| 754 | GO Biological Processes | GO:0048144 | fibroblast proliferation |
| 755 | GO Biological Processes | GO:0004511 | tyrosine 3-monooxygenase activity |
| 756 | GO Biological Processes | GO:0004883 | glucocorticoid receptor activity |
| 757 | GO Biological Processes | GO:0006913 | nucleocytoplasmic transport |
| 758 | GO Biological Processes | GO:0001666 | response to hypoxia |
| 759 | GO Biological Processes | GO:1990928 | response to amino acid starvation |
| 760 | GO Biological Processes | GO:0001101 | response to acid chemical |
| 761 | GO Biological Processes | GO:0051169 | nuclear transport |
| 762 | GO Biological Processes | GO:0050801 | ion homeostasis |
| 763 | GO Biological Processes | GO:2000142 | regulation of DNA-templated transcription, initiation |
| 764 | GO Biological Processes | GO:0055082 | cellular chemical homeostasis |
| 765 | GO Biological Processes | GO:0001775 | cell activation |
| 766 | GO Biological Processes | GO:0002384 | hepatic immune response |
| 767 | GO Biological Processes | GO:0018963 | phthalate metabolic process |
| 768 | GO Biological Processes | GO:0019858 | cytosine metabolic process |
| 769 | GO Biological Processes | GO:0032625 | interleukin-21 production |
| 770 | GO Biological Processes | GO:0032665 | regulation of interleukin-21 production |
| 771 | GO Biological Processes | GO:0032745 | positive regulation of interleukin-21 production |
| 772 | GO Biological Processes | GO:0042418 | epinephrine biosynthetic process |
| 773 | GO Biological Processes | GO:0043006 | activation of phospholipase A2 activity by calcium-mediated signaling |
| 774 | GO Biological Processes | GO:0052314 | phytoalexin metabolic process |
| 775 | GO Biological Processes | GO:0060694 | regulation of cholesterol transporter activity |
| 776 | GO Biological Processes | GO:0071306 | cellular response to vitamin E |
| 777 | GO Biological Processes | GO:0072343 | pancreatic stellate cell proliferation |
| 778 | GO Biological Processes | GO:0090362 | positive regulation of platelet-derived growth factor production |
| 779 | GO Biological Processes | GO:1901558 | response to metformin |
| 780 | GO Biological Processes | GO:1903943 | regulation of hepatocyte apoptotic process |
| 781 | GO Biological Processes | GO:1903944 | negative regulation of hepatocyte apoptotic process |
| 782 | GO Biological Processes | GO:2000229 | regulation of pancreatic stellate cell proliferation |
| 783 | GO Biological Processes | GO:0014031 | mesenchymal cell development |
| 784 | GO Biological Processes | GO:0046889 | positive regulation of lipid biosynthetic process |
| 785 | GO Biological Processes | GO:0048864 | stem cell development |
| 786 | GO Biological Processes | GO:0010833 | telomere maintenance via telomere lengthening |
| 787 | GO Biological Processes | GO:0051098 | regulation of binding |
| 788 | GO Biological Processes | GO:0036293 | response to decreased oxygen levels |
| 789 | GO Biological Processes | GO:0014706 | striated muscle tissue development |
| 790 | GO Biological Processes | GO:0030111 | regulation of Wnt signaling pathway |
| 791 | GO Biological Processes | GO:0043604 | amide biosynthetic process |
| 792 | GO Biological Processes | GO:0001221 | transcription cofactor binding |
| 793 | GO Biological Processes | GO:0006417 | regulation of translation |
| 794 | GO Biological Processes | GO:0001227 | DNA-binding transcription repressor activity, RNA polymerase II-specific |
| 795 | GO Biological Processes | GO:0006897 | endocytosis |
| 796 | GO Biological Processes | GO:0043535 | regulation of blood vessel endothelial cell migration |
| 797 | GO Biological Processes | GO:0007517 | muscle organ development |
| 798 | GO Biological Processes | GO:0018108 | peptidyl-tyrosine phosphorylation |
| 799 | GO Biological Processes | GO:0014033 | neural crest cell differentiation |
| 800 | GO Biological Processes | GO:0018212 | peptidyl-tyrosine modification |
| 801 | GO Biological Processes | GO:0070542 | response to fatty acid |
| 802 | GO Biological Processes | GO:1904035 | regulation of epithelial cell apoptotic process |
| 803 | GO Biological Processes | GO:0006955 | immune response |
| 804 | GO Biological Processes | GO:0006518 | peptide metabolic process |
| 805 | GO Biological Processes | GO:0006909 | phagocytosis |
| 806 | GO Biological Processes | GO:0035249 | synaptic transmission, glutamatergic |
| 807 | GO Biological Processes | GO:0017144 | drug metabolic process |
| 808 | GO Biological Processes | GO:0009165 | nucleotide biosynthetic process |
| 809 | GO Biological Processes | GO:0046849 | bone remodeling |
| 810 | GO Biological Processes | GO:0060537 | muscle tissue development |
| 811 | GO Biological Processes | GO:0008289 | lipid binding |
| 812 | GO Biological Processes | GO:0070491 | repressing transcription factor binding |
| 813 | GO Biological Processes | GO:0043066 | negative regulation of apoptotic process |
| 814 | GO Biological Processes | GO:0030641 | regulation of cellular pH |
| 815 | GO Biological Processes | GO:0098609 | cell-cell adhesion |
| 816 | GO Biological Processes | GO:1901293 | nucleoside phosphate biosynthetic process |
| 817 | GO Biological Processes | GO:0006796 | phosphate-containing compound metabolic process |
| 818 | GO Biological Processes | GO:1903426 | regulation of reactive oxygen species biosynthetic process |
| 819 | GO Biological Processes | GO:0016301 | kinase activity |
| 820 | GO Biological Processes | GO:0006974 | cellular response to DNA damage stimulus |
| 821 | GO Biological Processes | GO:0019216 | regulation of lipid metabolic process |
| 822 | GO Biological Processes | GO:0006793 | phosphorus metabolic process |
| 823 | GO Biological Processes | GO:0043549 | regulation of kinase activity |
| 824 | GO Biological Processes | GO:0060191 | regulation of lipase activity |
| 825 | GO Biological Processes | GO:0097327 | response to antineoplastic agent |
| 826 | GO Biological Processes | GO:0019538 | protein metabolic process |
| 827 | GO Biological Processes | GO:0000122 | negative regulation of transcription by RNA polymerase II |
| 828 | GO Biological Processes | GO:0032091 | negative regulation of protein binding |
| 829 | GO Biological Processes | GO:0050778 | positive regulation of immune response |
| 830 | GO Biological Processes | GO:0032501 | multicellular organismal process |
| 831 | GO Biological Processes | GO:0070498 | interleukin-1-mediated signaling pathway |
| 832 | GO Biological Processes | GO:0007399 | nervous system development |
| 833 | GO Biological Processes | GO:0042136 | neurotransmitter biosynthetic process |
| 834 | GO Biological Processes | GO:0031331 | positive regulation of cellular catabolic process |
| 835 | GO Biological Processes | GO:0015908 | fatty acid transport |
| 836 | GO Biological Processes | GO:0051153 | regulation of striated muscle cell differentiation |
| 837 | GO Biological Processes | GO:0006885 | regulation of pH |
| 838 | GO Biological Processes | GO:0032963 | collagen metabolic process |
| 839 | GO Biological Processes | GO:0015960 | diadenosine polyphosphate biosynthetic process |
| 840 | GO Biological Processes | GO:0015965 | diadenosine tetraphosphate metabolic process |
| 841 | GO Biological Processes | GO:0015966 | diadenosine tetraphosphate biosynthetic process |
| 842 | GO Biological Processes | GO:0032227 | negative regulation of synaptic transmission, dopaminergic |
| 843 | GO Biological Processes | GO:0033076 | isoquinoline alkaloid metabolic process |
| 844 | GO Biological Processes | GO:0042214 | terpene metabolic process |
| 845 | GO Biological Processes | GO:0042414 | epinephrine metabolic process |
| 846 | GO Biological Processes | GO:0060523 | prostate epithelial cord elongation |
| 847 | GO Biological Processes | GO:0090360 | platelet-derived growth factor production |
| 848 | GO Biological Processes | GO:0090361 | regulation of platelet-derived growth factor production |
| 849 | GO Biological Processes | GO:1902722 | positive regulation of prolactin secretion |
| 850 | GO Biological Processes | GO:2000634 | regulation of primary miRNA processing |
| 851 | GO Biological Processes | GO:2000635 | negative regulation of primary miRNA processing |
| 852 | GO Biological Processes | GO:0035258 | steroid hormone receptor binding |
| 853 | GO Biological Processes | GO:0008270 | zinc ion binding |
| 854 | GO Biological Processes | GO:0038023 | signaling receptor activity |
| 855 | GO Biological Processes | GO:0004666 | prostaglandin-endoperoxide synthase activity |
| 856 | GO Biological Processes | GO:0005006 | epidermal growth factor-activated receptor activity |
| 857 | GO Biological Processes | GO:0051117 | ATPase binding |
| 858 | GO Biological Processes | GO:0051219 | phosphoprotein binding |
| 859 | GO Biological Processes | GO:0001558 | regulation of cell growth |
| 860 | GO Biological Processes | GO:0008543 | fibroblast growth factor receptor signaling pathway |
| 861 | GO Biological Processes | GO:0031329 | regulation of cellular catabolic process |
| 862 | GO Biological Processes | GO:0010876 | lipid localization |
| 863 | GO Biological Processes | GO:0009890 | negative regulation of biosynthetic process |
| 864 | GO Biological Processes | GO:0060089 | molecular transducer activity |
| 865 | GO Biological Processes | GO:0042303 | molting cycle |
| 866 | GO Biological Processes | GO:0042633 | hair cycle |
| 867 | GO Biological Processes | GO:0007600 | sensory perception |
| 868 | GO Biological Processes | GO:0022407 | regulation of cell-cell adhesion |
| 869 | GO Biological Processes | GO:0048568 | embryonic organ development |
| 870 | GO Biological Processes | GO:0060284 | regulation of cell development |
| 871 | GO Biological Processes | GO:0033365 | protein localization to organelle |
| 872 | GO Biological Processes | GO:0043534 | blood vessel endothelial cell migration |
| 873 | GO Biological Processes | GO:0034248 | regulation of cellular amide metabolic process |
| 874 | GO Biological Processes | GO:0001676 | long-chain fatty acid metabolic process |
| 875 | GO Biological Processes | GO:1904019 | epithelial cell apoptotic process |
| 876 | GO Biological Processes | GO:0062012 | regulation of small molecule metabolic process |
| 877 | GO Biological Processes | GO:0003677 | DNA binding |
| 878 | GO Biological Processes | GO:0005496 | steroid binding |
| 879 | GO Biological Processes | GO:0042562 | hormone binding |
| 880 | GO Biological Processes | GO:0050804 | modulation of chemical synaptic transmission |
| 881 | GO Biological Processes | GO:0021782 | glial cell development |
| 882 | GO Biological Processes | GO:0030004 | cellular monovalent inorganic cation homeostasis |
| 883 | GO Biological Processes | GO:0099177 | regulation of trans-synaptic signaling |
| 884 | GO Biological Processes | GO:0007613 | memory |
| 885 | GO Biological Processes | GO:0042471 | ear morphogenesis |
| 886 | GO Biological Processes | GO:0043279 | response to alkaloid |
| 887 | GO Biological Processes | GO:0033559 | unsaturated fatty acid metabolic process |
| 888 | GO Biological Processes | GO:0045667 | regulation of osteoblast differentiation |
| 889 | GO Biological Processes | GO:1903409 | reactive oxygen species biosynthetic process |
| 890 | GO Biological Processes | GO:0022612 | gland morphogenesis |
| 891 | GO Biological Processes | GO:0016032 | viral process |
| 892 | GO Biological Processes | GO:0015842 | aminergic neurotransmitter loading into synaptic vesicle |
| 893 | GO Biological Processes | GO:0032431 | activation of phospholipase A2 activity |
| 894 | GO Biological Processes | GO:0033590 | response to cobalamin |
| 895 | GO Biological Processes | GO:0033594 | response to hydroxyisoflavone |
| 896 | GO Biological Processes | GO:0046684 | response to pyrethroid |
| 897 | GO Biological Processes | GO:0060737 | prostate gland morphogenetic growth |
| 898 | GO Biological Processes | GO:0060750 | epithelial cell proliferation involved in mammary gland duct elongation |
| 899 | GO Biological Processes | GO:0061888 | regulation of astrocyte activation |
| 900 | GO Biological Processes | GO:0071284 | cellular response to lead ion |
| 901 | GO Biological Processes | GO:0090271 | positive regulation of fibroblast growth factor production |
| 902 | GO Biological Processes | GO:0099179 | regulation of synaptic membrane adhesion |
| 903 | GO Biological Processes | GO:0110112 | regulation of lipid transporter activity |
| 904 | GO Biological Processes | GO:2000676 | positive regulation of type B pancreatic cell apoptotic process |
| 905 | GO Biological Processes | GO:0016772 | transferase activity, transferring phosphorus-containing groups |
| 906 | GO Biological Processes | GO:0042752 | regulation of circadian rhythm |
| 907 | GO Biological Processes | GO:0031175 | neuron projection development |
| 908 | GO Biological Processes | GO:0071621 | granulocyte chemotaxis |
| 909 | GO Biological Processes | GO:0098657 | import into cell |
| 910 | GO Biological Processes | GO:0034056 | estrogen response element binding |
| 911 | GO Biological Processes | GO:0006690 | icosanoid metabolic process |
| 912 | GO Biological Processes | GO:0005783 | endoplasmic reticulum |
| 913 | GO Biological Processes | GO:0036094 | small molecule binding |
| 914 | GO Biological Processes | GO:0032368 | regulation of lipid transport |
| 915 | GO Biological Processes | GO:0009896 | positive regulation of catabolic process |
| 916 | GO Biological Processes | GO:0050769 | positive regulation of neurogenesis |
| 917 | GO Biological Processes | GO:0001228 | DNA-binding transcription activator activity, RNA polymerase II-specific |
| 918 | GO Biological Processes | GO:0000902 | cell morphogenesis |
| 919 | GO Biological Processes | GO:0035296 | regulation of tube diameter |
| 920 | GO Biological Processes | GO:0050880 | regulation of blood vessel size |
| 921 | GO Biological Processes | GO:0097746 | regulation of blood vessel diameter |
| 922 | GO Biological Processes | GO:0016049 | cell growth |
| 923 | GO Biological Processes | GO:0035150 | regulation of tube size |
| 924 | GO Biological Processes | GO:0006807 | nitrogen compound metabolic process |
| 925 | GO Biological Processes | GO:0002433 | immune response-regulating cell surface receptor signaling pathway involved in phagocytosis |
| 926 | GO Biological Processes | GO:0038096 | Fc-gamma receptor signaling pathway involved in phagocytosis |
| 927 | GO Biological Processes | GO:0010750 | positive regulation of nitric oxide mediated signal transduction |
| 928 | GO Biological Processes | GO:0060687 | regulation of branching involved in prostate gland morphogenesis |
| 929 | GO Biological Processes | GO:0060751 | branch elongation involved in mammary gland duct branching |
| 930 | GO Biological Processes | GO:0080184 | response to phenylpropanoid |
| 931 | GO Biological Processes | GO:1902219 | negative regulation of intrinsic apoptotic signaling pathway in response to osmotic stress |
| 932 | GO Biological Processes | GO:1902512 | positive regulation of apoptotic DNA fragmentation |
| 933 | GO Biological Processes | GO:1905563 | negative regulation of vascular endothelial cell proliferation |
| 934 | GO Biological Processes | GO:1905599 | positive regulation of low-density lipoprotein receptor activity |
| 935 | GO Biological Processes | GO:2000660 | negative regulation of interleukin-1-mediated signaling pathway |
| 936 | GO Biological Processes | GO:0002446 | neutrophil mediated immunity |
| 937 | GO Biological Processes | GO:0038094 | Fc-gamma receptor signaling pathway |
| 938 | GO Biological Processes | GO:0044344 | cellular response to fibroblast growth factor stimulus |
| 939 | GO Biological Processes | GO:0044403 | symbiont process |
| 940 | GO Biological Processes | GO:0005770 | late endosome |
| 941 | GO Biological Processes | GO:0001093 | TFIIB-class transcription factor binding |
| 942 | GO Biological Processes | GO:0034617 | tetrahydrobiopterin binding |
| 943 | GO Biological Processes | GO:0048408 | epidermal growth factor binding |
| 944 | GO Biological Processes | GO:0050692 | DBD domain binding |
| 945 | GO Biological Processes | GO:0097489 | multivesicular body, internal vesicle lumen |
| 946 | GO Biological Processes | GO:0001819 | positive regulation of cytokine production |
| 947 | GO Biological Processes | GO:0048871 | multicellular organismal homeostasis |
| 948 | GO Biological Processes | GO:0050900 | leukocyte migration |
| 949 | GO Biological Processes | GO:0090101 | negative regulation of transmembrane receptor protein serine/threonine kinase signaling pathway |
| 950 | GO Biological Processes | GO:0002431 | Fc receptor mediated stimulatory signaling pathway |
| 951 | GO Biological Processes | GO:0030879 | mammary gland development |
| 952 | GO Biological Processes | GO:0045471 | response to ethanol |
| 953 | GO Biological Processes | GO:0051817 | modification of morphology or physiology of other organism involved in symbiotic interaction |
| 954 | GO Biological Processes | GO:0038127 | ERBB signaling pathway |
| 955 | GO Biological Processes | GO:0060359 | response to ammonium ion |
| 956 | GO Biological Processes | GO:0071774 | response to fibroblast growth factor |
| 957 | GO Biological Processes | GO:0006720 | isoprenoid metabolic process |
| 958 | GO Biological Processes | GO:0072594 | establishment of protein localization to organelle |
| 959 | GO Biological Processes | GO:0016055 | Wnt signaling pathway |
| 960 | GO Biological Processes | GO:0007030 | Golgi organization |
| 961 | GO Biological Processes | GO:0198738 | cell-cell signaling by wnt |
| 962 | GO Biological Processes | GO:0044419 | interspecies interaction between organisms |
| 963 | GO Biological Processes | GO:0007612 | learning |
| 964 | GO Biological Processes | GO:0019359 | nicotinamide nucleotide biosynthetic process |
| 965 | GO Biological Processes | GO:0019363 | pyridine nucleotide biosynthetic process |
| 966 | GO Biological Processes | GO:0097530 | granulocyte migration |
| 967 | GO Biological Processes | GO:0001889 | liver development |
| 968 | GO Biological Processes | GO:0042421 | norepinephrine biosynthetic process |
| 969 | GO Biological Processes | GO:0060526 | prostate glandular acinus morphogenesis |
| 970 | GO Biological Processes | GO:0060527 | prostate epithelial cord arborization involved in prostate glandular acinus morphogenesis |
| 971 | GO Biological Processes | GO:0061470 | T follicular helper cell differentiation |
| 972 | GO Biological Processes | GO:0070257 | positive regulation of mucus secretion |
| 973 | GO Biological Processes | GO:0090269 | fibroblast growth factor production |
| 974 | GO Biological Processes | GO:0090270 | regulation of fibroblast growth factor production |
| 975 | GO Biological Processes | GO:1902218 | regulation of intrinsic apoptotic signaling pathway in response to osmotic stress |
| 976 | GO Biological Processes | GO:1903626 | positive regulation of DNA catabolic process |
| 977 | GO Biological Processes | GO:0009411 | response to UV |
| 978 | GO Biological Processes | GO:0072525 | pyridine-containing compound biosynthetic process |
| 979 | GO Biological Processes | GO:0030509 | BMP signaling pathway |
| 980 | GO Biological Processes | GO:0014074 | response to purine-containing compound |
| 981 | GO Biological Processes | GO:0061008 | hepaticobiliary system development |
| 982 | GO Biological Processes | GO:0042133 | neurotransmitter metabolic process |
| 983 | GO Biological Processes | GO:0032989 | cellular component morphogenesis |
| 984 | GO Biological Processes | GO:0060627 | regulation of vesicle-mediated transport |
| 985 | GO Biological Processes | GO:0016043 | cellular component organization |
| 986 | GO Biological Processes | GO:0051962 | positive regulation of nervous system development |
| 987 | GO Biological Processes | GO:0043603 | cellular amide metabolic process |
| 988 | GO Biological Processes | GO:0030284 | estrogen receptor activity |
| 989 | GO Biological Processes | GO:0048273 | mitogen-activated protein kinase p38 binding |
| 990 | GO Biological Processes | GO:0034613 | cellular protein localization |
| 991 | GO Biological Processes | GO:0044260 | cellular macromolecule metabolic process |
| 992 | GO Biological Processes | GO:0002262 | myeloid cell homeostasis |
| 993 | GO Biological Processes | GO:0030518 | intracellular steroid hormone receptor signaling pathway |
| 994 | GO Biological Processes | GO:0070727 | cellular macromolecule localization |
| 995 | GO Biological Processes | GO:0002444 | myeloid leukocyte mediated immunity |
| 996 | GO Biological Processes | GO:0010720 | positive regulation of cell development |
| 997 | GO Biological Processes | GO:0044432 | endoplasmic reticulum part |
| 998 | GO Biological Processes | GO:0031983 | vesicle lumen |
| 999 | GO Biological Processes | GO:0005634 | nucleus |
| 1000 | GO Biological Processes | GO:0002521 | leukocyte differentiation |
| 1001 | GO Biological Processes | GO:0051147 | regulation of muscle cell differentiation |
| 1002 | GO Biological Processes | GO:0009820 | alkaloid metabolic process |
| 1003 | GO Biological Processes | GO:0031394 | positive regulation of prostaglandin biosynthetic process |
| 1004 | GO Biological Processes | GO:0031622 | positive regulation of fever generation |
| 1005 | GO Biological Processes | GO:0032430 | positive regulation of phospholipase A2 activity |
| 1006 | GO Biological Processes | GO:0035095 | behavioral response to nicotine |
| 1007 | GO Biological Processes | GO:0060745 | mammary gland branching involved in pregnancy |
| 1008 | GO Biological Processes | GO:0070459 | prolactin secretion |
| 1009 | GO Biological Processes | GO:0071455 | cellular response to hyperoxia |
| 1010 | GO Biological Processes | GO:1900019 | regulation of protein kinase C activity |
| 1011 | GO Biological Processes | GO:1900020 | positive regulation of protein kinase C activity |
| 1012 | GO Biological Processes | GO:1904415 | regulation of xenophagy |
| 1013 | GO Biological Processes | GO:1904417 | positive regulation of xenophagy |
| 1014 | GO Biological Processes | GO:1905461 | positive regulation of vascular associated smooth muscle cell apoptotic process |
| 1015 | GO Biological Processes | GO:1990384 | hyaloid vascular plexus regression |
| 1016 | GO Biological Processes | GO:0010817 | regulation of hormone levels |
| 1017 | GO Biological Processes | GO:0003018 | vascular process in circulatory system |
| 1018 | GO Biological Processes | GO:0031214 | biomineral tissue development |
| 1019 | GO Biological Processes | GO:0055067 | monovalent inorganic cation homeostasis |
| 1020 | GO Biological Processes | GO:0051100 | negative regulation of binding |
| 1021 | GO Biological Processes | GO:0071772 | response to BMP |
| 1022 | GO Biological Processes | GO:0071773 | cellular response to BMP stimulus |
| 1023 | GO Biological Processes | GO:0043168 | anion binding |
| 1024 | GO Biological Processes | GO:0035257 | nuclear hormone receptor binding |
| 1025 | GO Biological Processes | GO:0044267 | cellular protein metabolic process |
| 1026 | GO Biological Processes | GO:0001959 | regulation of cytokine-mediated signaling pathway |
| 1027 | GO Biological Processes | GO:0038095 | Fc-epsilon receptor signaling pathway |
| 1028 | GO Biological Processes | GO:0010594 | regulation of endothelial cell migration |
| 1029 | GO Biological Processes | GO:0048639 | positive regulation of developmental growth |
| 1030 | GO Biological Processes | GO:0050544 | arachidonic acid binding |
| 1031 | GO Biological Processes | GO:1990239 | steroid hormone binding |
| 1032 | GO Biological Processes | GO:0000723 | telomere maintenance |
| 1033 | GO Biological Processes | GO:0016482 | cytosolic transport |
| 1034 | GO Biological Processes | GO:0044238 | primary metabolic process |
| 1035 | GO Biological Processes | GO:0000978 | RNA polymerase II proximal promoter sequence-specific DNA binding |
| 1036 | GO Biological Processes | GO:0000987 | proximal promoter sequence-specific DNA binding |
| 1037 | GO Biological Processes | GO:0003682 | chromatin binding |
| 1038 | GO Biological Processes | GO:0016705 | oxidoreductase activity, acting on paired donors, with incorporation or reduction of molecular oxygen |
| 1039 | GO Biological Processes | GO:0005138 | interleukin-6 receptor binding |
| 1040 | GO Biological Processes | GO:0016714 | oxidoreductase activity, acting on paired donors, with incorporation or reduction of molecular oxygen, reduced pteridine as one donor, and incorporation of one atom of oxygen |
| 1041 | GO Biological Processes | GO:0035240 | dopamine binding |
| 1042 | GO Biological Processes | GO:0050542 | icosanoid binding |
| 1043 | GO Biological Processes | GO:0050543 | icosatetraenoic acid binding |
| 1044 | GO Biological Processes | GO:0050693 | LBD domain binding |
| 1045 | GO Biological Processes | GO:0048771 | tissue remodeling |
| 1046 | GO Biological Processes | GO:0001547 | antral ovarian follicle growth |
| 1047 | GO Biological Processes | GO:0002314 | germinal center B cell differentiation |
| 1048 | GO Biological Processes | GO:0006975 | DNA damage induced protein phosphorylation |
| 1049 | GO Biological Processes | GO:0008627 | intrinsic apoptotic signaling pathway in response to osmotic stress |
| 1050 | GO Biological Processes | GO:0033129 | positive regulation of histone phosphorylation |
| 1051 | GO Biological Processes | GO:0035900 | response to isolation stress |
| 1052 | GO Biological Processes | GO:0060331 | negative regulation of response to interferon-gamma |
| 1053 | GO Biological Processes | GO:0060336 | negative regulation of interferon-gamma-mediated signaling pathway |
| 1054 | GO Biological Processes | GO:0090091 | positive regulation of extracellular matrix disassembly |
| 1055 | GO Biological Processes | GO:0090400 | stress-induced premature senescence |
| 1056 | GO Biological Processes | GO:0098700 | neurotransmitter loading into synaptic vesicle |
| 1057 | GO Biological Processes | GO:2000659 | regulation of interleukin-1-mediated signaling pathway |
| 1058 | GO Biological Processes | GO:0090288 | negative regulation of cellular response to growth factor stimulus |
| 1059 | GO Biological Processes | GO:0070435 | Shc-EGFR complex |
| 1060 | GO Biological Processes | GO:0044446 | intracellular organelle part |
| 1061 | GO Biological Processes | GO:0012505 | endomembrane system |
| 1062 | GO Biological Processes | GO:0048598 | embryonic morphogenesis |
| 1063 | GO Biological Processes | GO:0005769 | early endosome |
| 1064 | GO Biological Processes | GO:0005819 | spindle |
| 1065 | GO Biological Processes | GO:0071840 | cellular component organization or biogenesis |
| 1066 | GO Biological Processes | GO:0043235 | receptor complex |
| 1067 | GO Biological Processes | GO:0005925 | focal adhesion |
| 1068 | GO Biological Processes | GO:0044422 | organelle part |
| 1069 | GO Biological Processes | GO:0005924 | cell-substrate adherens junction |
| 1070 | GO Biological Processes | GO:0005739 | mitochondrion |
| 1071 | GO Biological Processes | GO:0030055 | cell-substrate junction |
| 1072 | GO Biological Processes | GO:0005896 | interleukin-6 receptor complex |
| 1073 | GO Biological Processes | GO:0046890 | regulation of lipid biosynthetic process |
| 1074 | GO Biological Processes | GO:0060759 | regulation of response to cytokine stimulus |
| 1075 | GO Biological Processes | GO:0044237 | cellular metabolic process |
| 1076 | GO Biological Processes | GO:0015718 | monocarboxylic acid transport |
| 1077 | GO Biological Processes | GO:0051897 | positive regulation of protein kinase B signaling |
| 1078 | GO Biological Processes | GO:1901568 | fatty acid derivative metabolic process |
| 1079 | GO Biological Processes | GO:0071347 | cellular response to interleukin-1 |
| 1080 | GO Biological Processes | GO:0045786 | negative regulation of cell cycle |
| 1081 | GO Biological Processes | GO:0032200 | telomere organization |
| 1082 | GO Biological Processes | GO:0071466 | cellular response to xenobiotic stimulus |
| 1083 | GO Biological Processes | GO:0001781 | neutrophil apoptotic process |
| 1084 | GO Biological Processes | GO:0010749 | regulation of nitric oxide mediated signal transduction |
| 1085 | GO Biological Processes | GO:0015959 | diadenosine polyphosphate metabolic process |
| 1086 | GO Biological Processes | GO:0031620 | regulation of fever generation |
| 1087 | GO Biological Processes | GO:0070255 | regulation of mucus secretion |
| 1088 | GO Biological Processes | GO:0071316 | cellular response to nicotine |
| 1089 | GO Biological Processes | GO:1905288 | vascular associated smooth muscle cell apoptotic process |
| 1090 | GO Biological Processes | GO:1905459 | regulation of vascular associated smooth muscle cell apoptotic process |
| 1091 | GO Biological Processes | GO:2000553 | positive regulation of T-helper 2 cell cytokine production |
| 1092 | GO Biological Processes | GO:2001280 | positive regulation of unsaturated fatty acid biosynthetic process |
| 1093 | GO Biological Processes | GO:0010469 | regulation of signaling receptor activity |
| 1094 | GO Biological Processes | GO:0051276 | chromosome organization |
| 1095 | GO Biological Processes | GO:0030308 | negative regulation of cell growth |
| 1096 | GO Biological Processes | GO:0035821 | modification of morphology or physiology of other organism |
| 1097 | GO Biological Processes | GO:0048167 | regulation of synaptic plasticity |
| 1098 | GO Biological Processes | GO:0051427 | hormone receptor binding |
| 1099 | GO Biological Processes | GO:1905114 | cell surface receptor signaling pathway involved in cell-cell signaling |
| 1100 | GO Biological Processes | GO:0061061 | muscle structure development |
| 1101 | GO Biological Processes | GO:0051216 | cartilage development |
| 1102 | GO Biological Processes | GO:0051186 | cofactor metabolic process |
| 1103 | GO Biological Processes | GO:0009414 | response to water deprivation |
| 1104 | GO Biological Processes | GO:0009750 | response to fructose |
| 1105 | GO Biological Processes | GO:0031392 | regulation of prostaglandin biosynthetic process |
| 1106 | GO Biological Processes | GO:0031915 | positive regulation of synaptic plasticity |
| 1107 | GO Biological Processes | GO:0032000 | positive regulation of fatty acid beta-oxidation |
| 1108 | GO Biological Processes | GO:0045899 | positive regulation of RNA polymerase II transcriptional preinitiation complex assembly |
| 1109 | GO Biological Processes | GO:0060068 | vagina development |
| 1110 | GO Biological Processes | GO:0060525 | prostate glandular acinus development |
| 1111 | GO Biological Processes | GO:0060736 | prostate gland growth |
| 1112 | GO Biological Processes | GO:0061052 | negative regulation of cell growth involved in cardiac muscle cell development |
| 1113 | GO Biological Processes | GO:0070091 | glucagon secretion |
| 1114 | GO Biological Processes | GO:0070092 | regulation of glucagon secretion |
| 1115 | GO Biological Processes | GO:0070391 | response to lipoteichoic acid |
| 1116 | GO Biological Processes | GO:0071223 | cellular response to lipoteichoic acid |
| 1117 | GO Biological Processes | GO:2000674 | regulation of type B pancreatic cell apoptotic process |
| 1118 | GO Biological Processes | GO:0019362 | pyridine nucleotide metabolic process |
| 1119 | GO Biological Processes | GO:0046496 | nicotinamide nucleotide metabolic process |
| 1120 | GO Biological Processes | GO:0051336 | regulation of hydrolase activity |
| 1121 | GO Biological Processes | GO:0098805 | whole membrane |
| 1122 | GO Biological Processes | GO:0005635 | nuclear envelope |
| 1123 | GO Biological Processes | GO:1904813 | ficolin-1-rich granule lumen |
| 1124 | GO Biological Processes | GO:0097487 | multivesicular body, internal vesicle |
| 1125 | GO Biological Processes | GO:0071478 | cellular response to radiation |
| 1126 | GO Biological Processes | GO:0043902 | positive regulation of multi-organism process |
| 1127 | GO Biological Processes | GO:0006464 | cellular protein modification process |
| 1128 | GO Biological Processes | GO:0036211 | protein modification process |
| 1129 | GO Biological Processes | GO:0002757 | immune response-activating signal transduction |
| 1130 | GO Biological Processes | GO:0060429 | epithelium development |
| 1131 | GO Biological Processes | GO:0043065 | positive regulation of apoptotic process |
| 1132 | GO Biological Processes | GO:0002252 | immune effector process |
| 1133 | GO Biological Processes | GO:0002862 | negative regulation of inflammatory response to antigenic stimulus |
| 1134 | GO Biological Processes | GO:0072524 | pyridine-containing compound metabolic process |
| 1135 | GO Biological Processes | GO:0043068 | positive regulation of programmed cell death |
| 1136 | GO Biological Processes | GO:0016192 | vesicle-mediated transport |
| 1137 | GO Biological Processes | GO:0002274 | myeloid leukocyte activation |
| 1138 | GO Biological Processes | GO:0070374 | positive regulation of ERK1 and ERK2 cascade |
| 1139 | GO Biological Processes | GO:0002683 | negative regulation of immune system process |
| 1140 | GO Biological Processes | GO:0001660 | fever generation |
| 1141 | GO Biological Processes | GO:0010960 | magnesium ion homeostasis |
| 1142 | GO Biological Processes | GO:0019371 | cyclooxygenase pathway |
| 1143 | GO Biological Processes | GO:0031652 | positive regulation of heat generation |
| 1144 | GO Biological Processes | GO:0042416 | dopamine biosynthetic process |
| 1145 | GO Biological Processes | GO:0097011 | cellular response to granulocyte macrophage colony-stimulating factor stimulus |
| 1146 | GO Biological Processes | GO:0097012 | response to granulocyte macrophage colony-stimulating factor |
| 1147 | GO Biological Processes | GO:0097050 | type B pancreatic cell apoptotic process |
| 1148 | GO Biological Processes | GO:0098792 | xenophagy |
| 1149 | GO Biological Processes | GO:0106049 | regulation of cellular response to osmotic stress |
| 1150 | GO Biological Processes | GO:1902510 | regulation of apoptotic DNA fragmentation |
| 1151 | GO Biological Processes | GO:2001252 | positive regulation of chromosome organization |
| 1152 | GO Biological Processes | GO:0032940 | secretion by cell |
| 1153 | GO Biological Processes | GO:0045892 | negative regulation of transcription, DNA-templated |
| 1154 | GO Biological Processes | GO:1903507 | negative regulation of nucleic acid-templated transcription |
| 1155 | GO Biological Processes | GO:0043393 | regulation of protein binding |
| 1156 | GO Biological Processes | GO:0030003 | cellular cation homeostasis |
| 1157 | GO Biological Processes | GO:1902679 | negative regulation of RNA biosynthetic process |
| 1158 | GO Biological Processes | GO:0071704 | organic substance metabolic process |
| 1159 | GO Biological Processes | GO:0070555 | response to interleukin-1 |
| 1160 | GO Biological Processes | GO:0048812 | neuron projection morphogenesis |
| 1161 | GO Biological Processes | GO:0006006 | glucose metabolic process |
| 1162 | GO Biological Processes | GO:0009746 | response to hexose |
| 1163 | GO Biological Processes | GO:0004955 | prostaglandin receptor activity |
| 1164 | GO Biological Processes | GO:0006873 | cellular ion homeostasis |
| 1165 | GO Biological Processes | GO:0043281 | regulation of cysteine-type endopeptidase activity involved in apoptotic process |
| 1166 | GO Biological Processes | GO:0032096 | negative regulation of response to food |
| 1167 | GO Biological Processes | GO:0032099 | negative regulation of appetite |
| 1168 | GO Biological Processes | GO:0033197 | response to vitamin E |
| 1169 | GO Biological Processes | GO:0060442 | branching involved in prostate gland morphogenesis |
| 1170 | GO Biological Processes | GO:2001279 | regulation of unsaturated fatty acid biosynthetic process |
| 1171 | GO Biological Processes | GO:0002764 | immune response-regulating signaling pathway |
| 1172 | GO Biological Processes | GO:0034655 | nucleobase-containing compound catabolic process |
| 1173 | GO Biological Processes | GO:0120039 | plasma membrane bounded cell projection morphogenesis |
| 1174 | GO Biological Processes | GO:0002861 | regulation of inflammatory response to antigenic stimulus |
| 1175 | GO Biological Processes | GO:0006733 | oxidoreduction coenzyme metabolic process |
| 1176 | GO Biological Processes | GO:0034284 | response to monosaccharide |
| 1177 | GO Biological Processes | GO:0043542 | endothelial cell migration |
| 1178 | GO Biological Processes | GO:0048858 | cell projection morphogenesis |
| 1179 | GO Biological Processes | GO:0002573 | myeloid leukocyte differentiation |
| 1180 | GO Biological Processes | GO:0000981 | DNA-binding transcription factor activity, RNA polymerase II-specific |
| 1181 | GO Biological Processes | GO:0004954 | prostanoid receptor activity |
| 1182 | GO Biological Processes | GO:0008199 | ferric iron binding |
| 1183 | GO Biological Processes | GO:0090276 | regulation of peptide hormone secretion |
| 1184 | GO Biological Processes | GO:0051649 | establishment of localization in cell |
| 1185 | GO Biological Processes | GO:0043583 | ear development |
| 1186 | GO Biological Processes | GO:0090407 | organophosphate biosynthetic process |
| 1187 | GO Biological Processes | GO:0002376 | immune system process |
| 1188 | GO Biological Processes | GO:0002674 | negative regulation of acute inflammatory response |
| 1189 | GO Biological Processes | GO:0006570 | tyrosine metabolic process |
| 1190 | GO Biological Processes | GO:0007494 | midgut development |
| 1191 | GO Biological Processes | GO:0009635 | response to herbicide |
| 1192 | GO Biological Processes | GO:0032429 | regulation of phospholipase A2 activity |
| 1193 | GO Biological Processes | GO:0032494 | response to peptidoglycan |
| 1194 | GO Biological Processes | GO:0033127 | regulation of histone phosphorylation |
| 1195 | GO Biological Processes | GO:0035331 | negative regulation of hippo signaling |
| 1196 | GO Biological Processes | GO:0035745 | T-helper 2 cell cytokine production |
| 1197 | GO Biological Processes | GO:0042415 | norepinephrine metabolic process |
| 1198 | GO Biological Processes | GO:0045722 | positive regulation of gluconeogenesis |
| 1199 | GO Biological Processes | GO:0045898 | regulation of RNA polymerase II transcriptional preinitiation complex assembly |
| 1200 | GO Biological Processes | GO:0050872 | white fat cell differentiation |
| 1201 | GO Biological Processes | GO:0060100 | positive regulation of phagocytosis, engulfment |
| 1202 | GO Biological Processes | GO:0070254 | mucus secretion |
| 1203 | GO Biological Processes | GO:0071287 | cellular response to manganese ion |
| 1204 | GO Biological Processes | GO:0097284 | hepatocyte apoptotic process |
| 1205 | GO Biological Processes | GO:1903624 | regulation of DNA catabolic process |
| 1206 | GO Biological Processes | GO:1905155 | positive regulation of membrane invagination |
| 1207 | GO Biological Processes | GO:2000551 | regulation of T-helper 2 cell cytokine production |
| 1208 | GO Biological Processes | GO:0032990 | cell part morphogenesis |
| 1209 | GO Biological Processes | GO:0008285 | negative regulation of cell proliferation |
| 1210 | GO Biological Processes | GO:0000977 | RNA polymerase II regulatory region sequence-specific DNA binding |
| 1211 | GO Biological Processes | GO:0001012 | RNA polymerase II regulatory region DNA binding |
| 1212 | GO Biological Processes | GO:0008353 | RNA polymerase II CTD heptapeptide repeat kinase activity |
| 1213 | GO Biological Processes | GO:0097371 | MDM2/MDM4 family protein binding |
| 1214 | GO Biological Processes | GO:0019904 | protein domain specific binding |
| 1215 | GO Biological Processes | GO:0003700 | DNA-binding transcription factor activity |
| 1216 | GO Biological Processes | GO:0031406 | carboxylic acid binding |
| 1217 | GO Biological Processes | GO:0010942 | positive regulation of cell death |
| 1218 | GO Biological Processes | GO:0010632 | regulation of epithelial cell migration |
| 1219 | GO Biological Processes | GO:0071453 | cellular response to oxygen levels |
| 1220 | GO Biological Processes | GO:0031053 | primary miRNA processing |
| 1221 | GO Biological Processes | GO:0031650 | regulation of heat generation |
| 1222 | GO Biological Processes | GO:0032105 | negative regulation of response to extracellular stimulus |
| 1223 | GO Biological Processes | GO:0032108 | negative regulation of response to nutrient levels |
| 1224 | GO Biological Processes | GO:0034356 | NAD biosynthesis via nicotinamide riboside salvage pathway |
| 1225 | GO Biological Processes | GO:0034393 | positive regulation of smooth muscle cell apoptotic process |
| 1226 | GO Biological Processes | GO:0036295 | cellular response to increased oxygen levels |
| 1227 | GO Biological Processes | GO:0042921 | glucocorticoid receptor signaling pathway |
| 1228 | GO Biological Processes | GO:0042953 | lipoprotein transport |
| 1229 | GO Biological Processes | GO:0045472 | response to ether |
| 1230 | GO Biological Processes | GO:0045986 | negative regulation of smooth muscle contraction |
| 1231 | GO Biological Processes | GO:0047484 | regulation of response to osmotic stress |
| 1232 | GO Biological Processes | GO:0051974 | negative regulation of telomerase activity |
| 1233 | GO Biological Processes | GO:0070141 | response to UV-A |
| 1234 | GO Biological Processes | GO:0072540 | T-helper 17 cell lineage commitment |
| 1235 | GO Biological Processes | GO:0150078 | positive regulation of neuroinflammatory response |
| 1236 | GO Biological Processes | GO:1902894 | negative regulation of pri-miRNA transcription by RNA polymerase II |
| 1237 | GO Biological Processes | GO:0002366 | leukocyte activation involved in immune response |
| 1238 | GO Biological Processes | GO:0043412 | macromolecule modification |
| 1239 | GO Biological Processes | GO:0002263 | cell activation involved in immune response |
| 1240 | GO Biological Processes | GO:0030155 | regulation of cell adhesion |
| 1241 | GO Biological Processes | GO:0046777 | protein autophosphorylation |
| 1242 | GO Biological Processes | GO:0090257 | regulation of muscle system process |
| 1243 | GO Biological Processes | GO:0009117 | nucleotide metabolic process |
| 1244 | GO Biological Processes | GO:0002253 | activation of immune response |
| 1245 | GO Biological Processes | GO:2000116 | regulation of cysteine-type endopeptidase activity |
| 1246 | GO Biological Processes | GO:0038093 | Fc receptor signaling pathway |
| 1247 | GO Biological Processes | GO:0010715 | regulation of extracellular matrix disassembly |
| 1248 | GO Biological Processes | GO:0031958 | corticosteroid receptor signaling pathway |
| 1249 | GO Biological Processes | GO:0044872 | lipoprotein localization |
| 1250 | GO Biological Processes | GO:0045820 | negative regulation of glycolytic process |
| 1251 | GO Biological Processes | GO:0060099 | regulation of phagocytosis, engulfment |
| 1252 | GO Biological Processes | GO:0006753 | nucleoside phosphate metabolic process |
| 1253 | GO Biological Processes | GO:0046700 | heterocycle catabolic process |
| 1254 | GO Biological Processes | GO:0001894 | tissue homeostasis |
| 1255 | GO Biological Processes | GO:0044459 | plasma membrane part |
| 1256 | GO Biological Processes | GO:0044270 | cellular nitrogen compound catabolic process |
| 1257 | GO Biological Processes | GO:0043177 | organic acid binding |
| 1258 | GO Biological Processes | GO:1901338 | catecholamine binding |
| 1259 | GO Biological Processes | GO:0004953 | icosanoid receptor activity |
| 1260 | GO Biological Processes | GO:0036041 | long-chain fatty acid binding |
| 1261 | GO Biological Processes | GO:0000976 | transcription regulatory region sequence-specific DNA binding |
| 1262 | GO Biological Processes | GO:0044212 | transcription regulatory region DNA binding |
| 1263 | GO Biological Processes | GO:0005524 | ATP binding |
| 1264 | GO Biological Processes | GO:0001067 | regulatory region nucleic acid binding |
| 1265 | GO Biological Processes | GO:0009743 | response to carbohydrate |
| 1266 | GO Biological Processes | GO:0007155 | cell adhesion |
| 1267 | GO Biological Processes | GO:0002437 | inflammatory response to antigenic stimulus |
| 1268 | GO Biological Processes | GO:0045926 | negative regulation of growth |
| 1269 | GO Biological Processes | GO:0071560 | cellular response to transforming growth factor beta stimulus |
| 1270 | GO Biological Processes | GO:0019439 | aromatic compound catabolic process |
| 1271 | GO Biological Processes | GO:0022610 | biological adhesion |
| 1272 | GO Biological Processes | GO:0006206 | pyrimidine nucleobase metabolic process |
| 1273 | GO Biological Processes | GO:0019372 | lipoxygenase pathway |
| 1274 | GO Biological Processes | GO:0030540 | female genitalia development |
| 1275 | GO Biological Processes | GO:0032225 | regulation of synaptic transmission, dopaminergic |
| 1276 | GO Biological Processes | GO:1901741 | positive regulation of myoblast fusion |
| 1277 | GO Biological Processes | GO:1905153 | regulation of membrane invagination |
| 1278 | GO Biological Processes | GO:0046903 | secretion |
| 1279 | GO Biological Processes | GO:0019318 | hexose metabolic process |
| 1280 | GO Biological Processes | GO:0061448 | connective tissue development |
| 1281 | GO Biological Processes | GO:0071559 | response to transforming growth factor beta |
| 1282 | GO Biological Processes | GO:0005912 | adherens junction |
| 1283 | GO Biological Processes | GO:0005654 | nucleoplasm |
| 1284 | GO Biological Processes | GO:0043204 | perikaryon |
| 1285 | GO Biological Processes | GO:0070161 | anchoring junction |
| 1286 | GO Biological Processes | GO:0098590 | plasma membrane region |
| 1287 | GO Biological Processes | GO:0090092 | regulation of transmembrane receptor protein serine/threonine kinase signaling pathway |
| 1288 | GO Biological Processes | GO:0002830 | positive regulation of type 2 immune response |
| 1289 | GO Biological Processes | GO:0017085 | response to insecticide |
| 1290 | GO Biological Processes | GO:0030812 | negative regulation of nucleotide catabolic process |
| 1291 | GO Biological Processes | GO:0045779 | negative regulation of bone resorption |
| 1292 | GO Biological Processes | GO:0051198 | negative regulation of coenzyme metabolic process |
| 1293 | GO Biological Processes | GO:0060033 | anatomical structure regression |
| 1294 | GO Biological Processes | GO:0070166 | enamel mineralization |
| 1295 | GO Biological Processes | GO:0071380 | cellular response to prostaglandin E stimulus |
| 1296 | GO Biological Processes | GO:1900119 | positive regulation of execution phase of apoptosis |
| 1297 | GO Biological Processes | GO:2000726 | negative regulation of cardiac muscle cell differentiation |
| 1298 | GO Biological Processes | GO:0043231 | intracellular membrane-bounded organelle |
| 1299 | GO Biological Processes | GO:0000785 | chromatin |
| 1300 | GO Biological Processes | GO:0030072 | peptide hormone secretion |
| 1301 | GO Biological Processes | GO:0045165 | cell fate commitment |
| 1302 | GO Biological Processes | GO:0050730 | regulation of peptidyl-tyrosine phosphorylation |
| 1303 | GO Biological Processes | GO:0051345 | positive regulation of hydrolase activity |
| 1304 | GO Biological Processes | GO:0046965 | retinoid X receptor binding |
| 1305 | GO Biological Processes | GO:0009108 | coenzyme biosynthetic process |
| 1306 | GO Biological Processes | GO:0032559 | adenyl ribonucleotide binding |
| 1307 | GO Biological Processes | GO:1990837 | sequence-specific double-stranded DNA binding |
| 1308 | GO Biological Processes | GO:0001226 | RNA polymerase II transcription corepressor binding |
| 1309 | GO Biological Processes | GO:0031435 | mitogen-activated protein kinase kinase kinase binding |
| 1310 | GO Biological Processes | GO:0030554 | adenyl nucleotide binding |
| 1311 | GO Biological Processes | GO:1901361 | organic cyclic compound catabolic process |
| 1312 | GO Biological Processes | GO:0008152 | metabolic process |
| 1313 | GO Biological Processes | GO:0002295 | T-helper cell lineage commitment |
| 1314 | GO Biological Processes | GO:0009415 | response to water |
| 1315 | GO Biological Processes | GO:0031649 | heat generation |
| 1316 | GO Biological Processes | GO:0032095 | regulation of response to food |
| 1317 | GO Biological Processes | GO:0035743 | CD4-positive, alpha-beta T cell cytokine production |
| 1318 | GO Biological Processes | GO:0060716 | labyrinthine layer blood vessel development |
| 1319 | GO Biological Processes | GO:0061050 | regulation of cell growth involved in cardiac muscle cell development |
| 1320 | GO Biological Processes | GO:0071318 | cellular response to ATP |
| 1321 | GO Biological Processes | GO:0071391 | cellular response to estrogen stimulus |
| 1322 | GO Biological Processes | GO:1903978 | regulation of microglial cell activation |
| 1323 | GO Biological Processes | GO:2001170 | negative regulation of ATP biosynthetic process |
| 1324 | GO Biological Processes | GO:2000113 | negative regulation of cellular macromolecule biosynthetic process |
| 1325 | GO Biological Processes | GO:0050789 | regulation of biological process |
| 1326 | GO Biological Processes | GO:0010558 | negative regulation of macromolecule biosynthetic process |
| 1327 | GO Biological Processes | GO:0032991 | protein-containing complex |
| 1328 | GO Biological Processes | GO:0048872 | homeostasis of number of cells |
| 1329 | GO Biological Processes | GO:0001780 | neutrophil homeostasis |
| 1330 | GO Biological Processes | GO:0009713 | catechol-containing compound biosynthetic process |
| 1331 | GO Biological Processes | GO:0016137 | glycoside metabolic process |
| 1332 | GO Biological Processes | GO:0030728 | ovulation |
| 1333 | GO Biological Processes | GO:0031000 | response to caffeine |
| 1334 | GO Biological Processes | GO:0032930 | positive regulation of superoxide anion generation |
| 1335 | GO Biological Processes | GO:0036270 | response to diuretic |
| 1336 | GO Biological Processes | GO:0042423 | catecholamine biosynthetic process |
| 1337 | GO Biological Processes | GO:0046851 | negative regulation of bone remodeling |
| 1338 | GO Biological Processes | GO:0060602 | branch elongation of an epithelium |
| 1339 | GO Biological Processes | GO:0071404 | cellular response to low-density lipoprotein particle stimulus |
| 1340 | GO Biological Processes | GO:0071498 | cellular response to fluid shear stress |
| 1341 | GO Biological Processes | GO:1900016 | negative regulation of cytokine production involved in inflammatory response |
| 1342 | GO Biological Processes | GO:1901739 | regulation of myoblast fusion |
| 1343 | GO Biological Processes | GO:0051146 | striated muscle cell differentiation |
| 1344 | GO Biological Processes | GO:0060322 | head development |
| 1345 | GO Biological Processes | GO:0055086 | nucleobase-containing small molecule metabolic process |
| 1346 | GO Biological Processes | GO:0046883 | regulation of hormone secretion |
| 1347 | GO Biological Processes | GO:0031998 | regulation of fatty acid beta-oxidation |
| 1348 | GO Biological Processes | GO:0032495 | response to muramyl dipeptide |
| 1349 | GO Biological Processes | GO:0033189 | response to vitamin A |
| 1350 | GO Biological Processes | GO:0035330 | regulation of hippo signaling |
| 1351 | GO Biological Processes | GO:0035357 | peroxisome proliferator activated receptor signaling pathway |
| 1352 | GO Biological Processes | GO:0043586 | tongue development |
| 1353 | GO Biological Processes | GO:0045780 | positive regulation of bone resorption |
| 1354 | GO Biological Processes | GO:0046852 | positive regulation of bone remodeling |
| 1355 | GO Biological Processes | GO:0060065 | uterus development |
| 1356 | GO Biological Processes | GO:0060749 | mammary gland alveolus development |
| 1357 | GO Biological Processes | GO:0061377 | mammary gland lobule development |
| 1358 | GO Biological Processes | GO:0101002 | ficolin-1-rich granule |
| 1359 | GO Biological Processes | GO:0001091 | RNA polymerase II basal transcription factor binding |
| 1360 | GO Biological Processes | GO:0031330 | negative regulation of cellular catabolic process |
| 1361 | GO Biological Processes | GO:0120036 | plasma membrane bounded cell projection organization |
| 1362 | GO Biological Processes | GO:0007411 | axon guidance |
| 1363 | GO Biological Processes | GO:0050708 | regulation of protein secretion |
| 1364 | GO Biological Processes | GO:0001818 | negative regulation of cytokine production |
| 1365 | GO Biological Processes | GO:0097485 | neuron projection guidance |
| 1366 | GO Biological Processes | GO:0043170 | macromolecule metabolic process |
| 1367 | GO Biological Processes | GO:0050767 | regulation of neurogenesis |
| 1368 | GO Biological Processes | GO:0006925 | inflammatory cell apoptotic process |
| 1369 | GO Biological Processes | GO:0030809 | negative regulation of nucleotide biosynthetic process |
| 1370 | GO Biological Processes | GO:0043373 | CD4-positive, alpha-beta T cell lineage commitment |
| 1371 | GO Biological Processes | GO:0045723 | positive regulation of fatty acid biosynthetic process |
| 1372 | GO Biological Processes | GO:0090050 | positive regulation of cell migration involved in sprouting angiogenesis |
| 1373 | GO Biological Processes | GO:0101023 | vascular endothelial cell proliferation |
| 1374 | GO Biological Processes | GO:1900017 | positive regulation of cytokine production involved in inflammatory response |
| 1375 | GO Biological Processes | GO:1900372 | negative regulation of purine nucleotide biosynthetic process |
| 1376 | GO Biological Processes | GO:1904996 | positive regulation of leukocyte adhesion to vascular endothelial cell |
| 1377 | GO Biological Processes | GO:1905562 | regulation of vascular endothelial cell proliferation |
| 1378 | GO Biological Processes | GO:0006325 | chromatin organization |
| 1379 | GO Biological Processes | GO:0032868 | response to insulin |
| 1380 | GO Biological Processes | GO:0070372 | regulation of ERK1 and ERK2 cascade |
| 1381 | GO Biological Processes | GO:0031327 | negative regulation of cellular biosynthetic process |
| 1382 | GO Biological Processes | GO:0060828 | regulation of canonical Wnt signaling pathway |
| 1383 | GO Biological Processes | GO:0001502 | cartilage condensation |
| 1384 | GO Biological Processes | GO:0007617 | mating behavior |
| 1385 | GO Biological Processes | GO:0032098 | regulation of appetite |
| 1386 | GO Biological Processes | GO:0032740 | positive regulation of interleukin-17 production |
| 1387 | GO Biological Processes | GO:0048714 | positive regulation of oligodendrocyte differentiation |
| 1388 | GO Biological Processes | GO:0051412 | response to corticosterone |
| 1389 | GO Biological Processes | GO:0070102 | interleukin-6-mediated signaling pathway |
| 1390 | GO Biological Processes | GO:0071379 | cellular response to prostaglandin stimulus |
| 1391 | GO Biological Processes | GO:0071636 | positive regulation of transforming growth factor beta production |
| 1392 | GO Biological Processes | GO:0030030 | cell projection organization |
| 1393 | GO Biological Processes | GO:0010631 | epithelial cell migration |
| 1394 | GO Biological Processes | GO:0005102 | signaling receptor binding |
| 1395 | GO Biological Processes | GO:0043565 | sequence-specific DNA binding |
| 1396 | GO Biological Processes | GO:0005996 | monosaccharide metabolic process |
| 1397 | GO Biological Processes | GO:0090132 | epithelium migration |
| 1398 | GO Biological Processes | GO:0090287 | regulation of cellular response to growth factor stimulus |
| 1399 | GO Biological Processes | GO:0002363 | alpha-beta T cell lineage commitment |
| 1400 | GO Biological Processes | GO:0010226 | response to lithium ion |
| 1401 | GO Biological Processes | GO:0032682 | negative regulation of chemokine production |
| 1402 | GO Biological Processes | GO:0032928 | regulation of superoxide anion generation |
| 1403 | GO Biological Processes | GO:0034104 | negative regulation of tissue remodeling |
| 1404 | GO Biological Processes | GO:0034390 | smooth muscle cell apoptotic process |
| 1405 | GO Biological Processes | GO:0034391 | regulation of smooth muscle cell apoptotic process |
| 1406 | GO Biological Processes | GO:0035994 | response to muscle stretch |
| 1407 | GO Biological Processes | GO:0045663 | positive regulation of myoblast differentiation |
| 1408 | GO Biological Processes | GO:0045932 | negative regulation of muscle contraction |
| 1409 | GO Biological Processes | GO:0055093 | response to hyperoxia |
| 1410 | GO Biological Processes | GO:0060444 | branching involved in mammary gland duct morphogenesis |
| 1411 | GO Biological Processes | GO:0072574 | hepatocyte proliferation |
| 1412 | GO Biological Processes | GO:0072575 | epithelial cell proliferation involved in liver morphogenesis |
| 1413 | GO Biological Processes | GO:0098743 | cell aggregation |
| 1414 | GO Biological Processes | GO:0097550 | transcriptional preinitiation complex |
| 1415 | GO Biological Processes | GO:0045202 | synapse |
| 1416 | GO Biological Processes | GO:0031981 | nuclear lumen |
| 1417 | GO Biological Processes | GO:0120025 | plasma membrane bounded cell projection |
| 1418 | GO Biological Processes | GO:0090130 | tissue migration |
| 1419 | GO Biological Processes | GO:0002726 | positive regulation of T cell cytokine production |
| 1420 | GO Biological Processes | GO:0010288 | response to lead ion |
| 1421 | GO Biological Processes | GO:0034505 | tooth mineralization |
| 1422 | GO Biological Processes | GO:0043369 | CD4-positive or CD8-positive, alpha-beta T cell lineage commitment |
| 1423 | GO Biological Processes | GO:0051195 | negative regulation of cofactor metabolic process |
| 1424 | GO Biological Processes | GO:0055022 | negative regulation of cardiac muscle tissue growth |
| 1425 | GO Biological Processes | GO:0060143 | positive regulation of syncytium formation by plasma membrane fusion |
| 1426 | GO Biological Processes | GO:0061117 | negative regulation of heart growth |
| 1427 | GO Biological Processes | GO:0072576 | liver morphogenesis |
| 1428 | GO Biological Processes | GO:0097186 | amelogenesis |
| 1429 | GO Biological Processes | GO:1903055 | positive regulation of extracellular matrix organization |
| 1430 | GO Biological Processes | GO:1904706 | negative regulation of vascular smooth muscle cell proliferation |
| 1431 | GO Biological Processes | GO:0002443 | leukocyte mediated immunity |
| 1432 | GO Biological Processes | GO:0001224 | RNA polymerase II transcription cofactor binding |
| 1433 | GO Biological Processes | GO:0006309 | apoptotic DNA fragmentation |
| 1434 | GO Biological Processes | GO:0032104 | regulation of response to extracellular stimulus |
| 1435 | GO Biological Processes | GO:0032107 | regulation of response to nutrient levels |
| 1436 | GO Biological Processes | GO:0034695 | response to prostaglandin E |
| 1437 | GO Biological Processes | GO:0042537 | benzene-containing compound metabolic process |
| 1438 | GO Biological Processes | GO:0060740 | prostate gland epithelium morphogenesis |
| 1439 | GO Biological Processes | GO:0070935 | 3'-UTR-mediated mRNA stabilization |
| 1440 | GO Biological Processes | GO:0002791 | regulation of peptide secretion |
| 1441 | GO Biological Processes | GO:0004709 | MAP kinase kinase kinase activity |
| 1442 | GO Biological Processes | GO:0008198 | ferrous iron binding |
| 1443 | GO Biological Processes | GO:0016702 | oxidoreductase activity, acting on single donors with incorporation of molecular oxygen, incorporation of two atoms of oxygen |
| 1444 | GO Biological Processes | GO:0017025 | TBP-class protein binding |
| 1445 | GO Biological Processes | GO:0042974 | retinoic acid receptor binding |
| 1446 | GO Biological Processes | GO:0010614 | negative regulation of cardiac muscle hypertrophy |
| 1447 | GO Biological Processes | GO:0060330 | regulation of response to interferon-gamma |
| 1448 | GO Biological Processes | GO:0060334 | regulation of interferon-gamma-mediated signaling pathway |
| 1449 | GO Biological Processes | GO:0046879 | hormone secretion |
| 1450 | GO Biological Processes | GO:0016701 | oxidoreductase activity, acting on single donors with incorporation of molecular oxygen |
| 1451 | GO Biological Processes | GO:1903037 | regulation of leukocyte cell-cell adhesion |
| 1452 | GO Biological Processes | GO:0003298 | physiological muscle hypertrophy |
| 1453 | GO Biological Processes | GO:0003301 | physiological cardiac muscle hypertrophy |
| 1454 | GO Biological Processes | GO:0007263 | nitric oxide mediated signal transduction |
| 1455 | GO Biological Processes | GO:0009651 | response to salt stress |
| 1456 | GO Biological Processes | GO:0010661 | positive regulation of muscle cell apoptotic process |
| 1457 | GO Biological Processes | GO:0042745 | circadian sleep/wake cycle |
| 1458 | GO Biological Processes | GO:0048596 | embryonic camera-type eye morphogenesis |
| 1459 | GO Biological Processes | GO:0048710 | regulation of astrocyte differentiation |
| 1460 | GO Biological Processes | GO:0050996 | positive regulation of lipid catabolic process |
| 1461 | GO Biological Processes | GO:0060142 | regulation of syncytium formation by plasma membrane fusion |
| 1462 | GO Biological Processes | GO:0060512 | prostate gland morphogenesis |
| 1463 | GO Biological Processes | GO:0061049 | cell growth involved in cardiac muscle cell development |
| 1464 | GO Biological Processes | GO:0099560 | synaptic membrane adhesion |
| 1465 | GO Biological Processes | GO:0051393 | alpha-actinin binding |
| 1466 | GO Biological Processes | GO:1901567 | fatty acid derivative binding |
| 1467 | GO Biological Processes | GO:0010721 | negative regulation of cell development |
| 1468 | GO Biological Processes | GO:0006898 | receptor-mediated endocytosis |
| 1469 | GO Biological Processes | GO:0002313 | mature B cell differentiation involved in immune response |
| 1470 | GO Biological Processes | GO:0014741 | negative regulation of muscle hypertrophy |
| 1471 | GO Biological Processes | GO:0060261 | positive regulation of transcription initiation from RNA polymerase II promoter |
| 1472 | GO Biological Processes | GO:0072539 | T-helper 17 cell differentiation |
| 1473 | GO Biological Processes | GO:1903579 | negative regulation of ATP metabolic process |
| 1474 | GO Biological Processes | GO:1904994 | regulation of leukocyte adhesion to vascular endothelial cell |
| 1475 | GO Biological Processes | GO:1990776 | response to angiotensin |
| 1476 | GO Biological Processes | GO:0009914 | hormone transport |
| 1477 | GO Biological Processes | GO:0051188 | cofactor biosynthetic process |
| 1478 | GO Biological Processes | GO:0030097 | hemopoiesis |
| 1479 | GO Biological Processes | GO:0003676 | nucleic acid binding |
| 1480 | GO Biological Processes | GO:0001516 | prostaglandin biosynthetic process |
| 1481 | GO Biological Processes | GO:0010800 | positive regulation of peptidyl-threonine phosphorylation |
| 1482 | GO Biological Processes | GO:0033028 | myeloid cell apoptotic process |
| 1483 | GO Biological Processes | GO:0035066 | positive regulation of histone acetylation |
| 1484 | GO Biological Processes | GO:0046457 | prostanoid biosynthetic process |
| 1485 | GO Biological Processes | GO:0055094 | response to lipoprotein particle |
| 1486 | GO Biological Processes | GO:0060674 | placenta blood vessel development |
| 1487 | GO Biological Processes | GO:0060070 | canonical Wnt signaling pathway |
| 1488 | GO Biological Processes | GO:0001222 | transcription corepressor binding |
| 1489 | GO Biological Processes | GO:0051960 | regulation of nervous system development |
| 1490 | GO Biological Processes | GO:0042995 | cell projection |
| 1491 | GO Biological Processes | GO:0050678 | regulation of epithelial cell proliferation |
| 1492 | GO Biological Processes | GO:0072330 | monocarboxylic acid biosynthetic process |
| 1493 | GO Biological Processes | GO:0002360 | T cell lineage commitment |
| 1494 | GO Biological Processes | GO:0003401 | axis elongation |
| 1495 | GO Biological Processes | GO:0008209 | androgen metabolic process |
| 1496 | GO Biological Processes | GO:0019098 | reproductive behavior |
| 1497 | GO Biological Processes | GO:0036296 | response to increased oxygen levels |
| 1498 | GO Biological Processes | GO:0045648 | positive regulation of erythrocyte differentiation |
| 1499 | GO Biological Processes | GO:0045737 | positive regulation of cyclin-dependent protein serine/threonine kinase activity |
| 1500 | GO Biological Processes | GO:0045987 | positive regulation of smooth muscle contraction |
| 1501 | GO Biological Processes | GO:0048147 | negative regulation of fibroblast proliferation |
| 1502 | GO Biological Processes | GO:0009056 | catabolic process |
| 1503 | GO Biological Processes | GO:0046942 | carboxylic acid transport |
| 1504 | GO Biological Processes | GO:0015849 | organic acid transport |
| 1505 | GO Biological Processes | GO:0035639 | purine ribonucleoside triphosphate binding |
| 1506 | GO Biological Processes | GO:0071168 | protein localization to chromatin |
| 1507 | GO Biological Processes | GO:0071295 | cellular response to vitamin |
| 1508 | GO Biological Processes | GO:0071402 | cellular response to lipoprotein particle stimulus |
| 1509 | GO Biological Processes | GO:1900543 | negative regulation of purine nucleotide metabolic process |
| 1510 | GO Biological Processes | GO:0001505 | regulation of neurotransmitter levels |
| 1511 | GO Biological Processes | GO:0051019 | mitogen-activated protein kinase binding |
| 1512 | GO Biological Processes | GO:0009615 | response to virus |
| 1513 | GO Biological Processes | GO:0048471 | perinuclear region of cytoplasm |
| 1514 | GO Biological Processes | GO:0031624 | ubiquitin conjugating enzyme binding |
| 1515 | GO Biological Processes | GO:0000737 | DNA catabolic process, endonucleolytic |
| 1516 | GO Biological Processes | GO:0002828 | regulation of type 2 immune response |
| 1517 | GO Biological Processes | GO:0034694 | response to prostaglandin |
| 1518 | GO Biological Processes | GO:0038128 | ERBB2 signaling pathway |
| 1519 | GO Biological Processes | GO:0042759 | long-chain fatty acid biosynthetic process |
| 1520 | GO Biological Processes | GO:0045980 | negative regulation of nucleotide metabolic process |
| 1521 | GO Biological Processes | GO:0046621 | negative regulation of organ growth |
| 1522 | GO Biological Processes | GO:0060603 | mammary gland duct morphogenesis |
| 1523 | GO Biological Processes | GO:0072538 | T-helper 17 type immune response |
| 1524 | GO Biological Processes | GO:0007159 | leukocyte cell-cell adhesion |
| 1525 | GO Biological Processes | GO:0001975 | response to amphetamine |
| 1526 | GO Biological Processes | GO:0048384 | retinoic acid receptor signaling pathway |
| 1527 | GO Biological Processes | GO:0055026 | negative regulation of cardiac muscle tissue development |
| 1528 | GO Biological Processes | GO:1903427 | negative regulation of reactive oxygen species biosynthetic process |
| 1529 | GO Biological Processes | GO:2000191 | regulation of fatty acid transport |
| 1530 | GO Biological Processes | GO:0042692 | muscle cell differentiation |
| 1531 | GO Biological Processes | GO:0046907 | intracellular transport |
| 1532 | GO Biological Processes | GO:0002335 | mature B cell differentiation |
| 1533 | GO Biological Processes | GO:0007202 | activation of phospholipase C activity |
| 1534 | GO Biological Processes | GO:0009112 | nucleobase metabolic process |
| 1535 | GO Biological Processes | GO:0030262 | apoptotic nuclear changes |
| 1536 | GO Biological Processes | GO:0033198 | response to ATP |
| 1537 | GO Biological Processes | GO:0034105 | positive regulation of tissue remodeling |
| 1538 | GO Biological Processes | GO:0034405 | response to fluid shear stress |
| 1539 | GO Biological Processes | GO:0043537 | negative regulation of blood vessel endothelial cell migration |
| 1540 | GO Biological Processes | GO:0070050 | neuron cellular homeostasis |
| 1541 | GO Biological Processes | GO:1900087 | positive regulation of G1/S transition of mitotic cell cycle |
| 1542 | GO Biological Processes | GO:1904031 | positive regulation of cyclin-dependent protein kinase activity |
| 1543 | GO Biological Processes | GO:2000144 | positive regulation of DNA-templated transcription, initiation |
| 1544 | GO Biological Processes | GO:2000725 | regulation of cardiac muscle cell differentiation |
| 1545 | GO Biological Processes | GO:2000758 | positive regulation of peptidyl-lysine acetylation |
| 1546 | GO Biological Processes | GO:0031410 | cytoplasmic vesicle |
| 1547 | GO Biological Processes | GO:0097708 | intracellular vesicle |
| 1548 | GO Biological Processes | GO:0009306 | protein secretion |
| 1549 | GO Biological Processes | GO:0006396 | RNA processing |
| 1550 | GO Biological Processes | GO:0009404 | toxin metabolic process |
| 1551 | GO Biological Processes | GO:0010831 | positive regulation of myotube differentiation |
| 1552 | GO Biological Processes | GO:0032620 | interleukin-17 production |
| 1553 | GO Biological Processes | GO:0032660 | regulation of interleukin-17 production |
| 1554 | GO Biological Processes | GO:0033280 | response to vitamin D |
| 1555 | GO Biological Processes | GO:0042462 | eye photoreceptor cell development |
| 1556 | GO Biological Processes | GO:0042755 | eating behavior |
| 1557 | GO Biological Processes | GO:0048048 | embryonic eye morphogenesis |
| 1558 | GO Biological Processes | GO:0032555 | purine ribonucleotide binding |
| 1559 | GO Biological Processes | GO:0065007 | biological regulation |
| 1560 | GO Biological Processes | GO:0017076 | purine nucleotide binding |
| 1561 | GO Biological Processes | GO:0032553 | ribonucleotide binding |
| 1562 | GO Biological Processes | GO:0006869 | lipid transport |
| 1563 | GO Biological Processes | GO:0019058 | viral life cycle |
| 1564 | GO Biological Processes | GO:0033044 | regulation of chromosome organization |
| 1565 | GO Biological Processes | GO:0006921 | cellular component disassembly involved in execution phase of apoptosis |
| 1566 | GO Biological Processes | GO:0010259 | multicellular organism aging |
| 1567 | GO Biological Processes | GO:0030224 | monocyte differentiation |
| 1568 | GO Biological Processes | GO:0042092 | type 2 immune response |
| 1569 | GO Biological Processes | GO:0051154 | negative regulation of striated muscle cell differentiation |
| 1570 | GO Biological Processes | GO:0051385 | response to mineralocorticoid |
| 1571 | GO Biological Processes | GO:0090322 | regulation of superoxide metabolic process |
| 1572 | GO Biological Processes | GO:1903131 | mononuclear cell differentiation |
| 1573 | GO Biological Processes | GO:0042805 | actinin binding |
| 1574 | GO Biological Processes | GO:0140110 | transcription regulator activity |
| 1575 | GO Biological Processes | GO:0019825 | oxygen binding |
| 1576 | GO Biological Processes | GO:0044390 | ubiquitin-like protein conjugating enzyme binding |
| 1577 | GO Biological Processes | GO:0002369 | T cell cytokine production |
| 1578 | GO Biological Processes | GO:0002724 | regulation of T cell cytokine production |
| 1579 | GO Biological Processes | GO:0006361 | transcription initiation from RNA polymerase I promoter |
| 1580 | GO Biological Processes | GO:0031076 | embryonic camera-type eye development |
| 1581 | GO Biological Processes | GO:0045070 | positive regulation of viral genome replication |
| 1582 | GO Biological Processes | GO:0051123 | RNA polymerase II preinitiation complex assembly |
| 1583 | GO Biological Processes | GO:0005504 | fatty acid binding |
| 1584 | GO Biological Processes | GO:0044433 | cytoplasmic vesicle part |
| 1585 | GO Biological Processes | GO:0046872 | metal ion binding |
| 1586 | GO Biological Processes | GO:0010907 | positive regulation of glucose metabolic process |
| 1587 | GO Biological Processes | GO:0016572 | histone phosphorylation |
| 1588 | GO Biological Processes | GO:0032941 | secretion by tissue |
| 1589 | GO Biological Processes | GO:0042417 | dopamine metabolic process |
| 1590 | GO Biological Processes | GO:0042554 | superoxide anion generation |
| 1591 | GO Biological Processes | GO:0043368 | positive T cell selection |
| 1592 | GO Biological Processes | GO:0046326 | positive regulation of glucose import |
| 1593 | GO Biological Processes | GO:0090049 | regulation of cell migration involved in sprouting angiogenesis |
| 1594 | GO Biological Processes | GO:2001171 | positive regulation of ATP biosynthetic process |
| 1595 | GO Biological Processes | GO:0009790 | embryo development |
| 1596 | GO Biological Processes | GO:0033162 | melanosome membrane |
| 1597 | GO Biological Processes | GO:0045009 | chitosome |
| 1598 | GO Biological Processes | GO:0090741 | pigment granule membrane |
| 1599 | GO Biological Processes | GO:0005794 | Golgi apparatus |
| 1600 | GO Biological Processes | GO:0007618 | mating |
| 1601 | GO Biological Processes | GO:0032094 | response to food |
| 1602 | GO Biological Processes | GO:0032733 | positive regulation of interleukin-10 production |
| 1603 | GO Biological Processes | GO:0045740 | positive regulation of DNA replication |
| 1604 | GO Biological Processes | GO:0045746 | negative regulation of Notch signaling pathway |
| 1605 | GO Biological Processes | GO:0048713 | regulation of oligodendrocyte differentiation |
| 1606 | GO Biological Processes | GO:0001046 | core promoter sequence-specific DNA binding |
| 1607 | GO Biological Processes | GO:0001047 | core promoter binding |
| 1608 | GO Biological Processes | GO:0051879 | Hsp90 protein binding |
| 1609 | GO Biological Processes | GO:0032735 | positive regulation of interleukin-12 production |
| 1610 | GO Biological Processes | GO:0045429 | positive regulation of nitric oxide biosynthetic process |
| 1611 | GO Biological Processes | GO:0071354 | cellular response to interleukin-6 |
| 1612 | GO Biological Processes | GO:0071470 | cellular response to osmotic stress |
| 1613 | GO Biological Processes | GO:0071634 | regulation of transforming growth factor beta production |
| 1614 | GO Biological Processes | GO:1900117 | regulation of execution phase of apoptosis |
| 1615 | GO Biological Processes | GO:0043169 | cation binding |
| 1616 | GO Biological Processes | GO:0006308 | DNA catabolic process |
| 1617 | GO Biological Processes | GO:0009072 | aromatic amino acid family metabolic process |
| 1618 | GO Biological Processes | GO:0071312 | cellular response to alkaloid |
| 1619 | GO Biological Processes | GO:1904037 | positive regulation of epithelial cell apoptotic process |
| 1620 | GO Biological Processes | GO:1904407 | positive regulation of nitric oxide metabolic process |
| 1621 | GO Biological Processes | GO:0002790 | peptide secretion |
| 1622 | GO Biological Processes | GO:0045687 | positive regulation of glial cell differentiation |
| 1623 | GO Biological Processes | GO:0071604 | transforming growth factor beta production |
| 1624 | GO Biological Processes | GO:2000279 | negative regulation of DNA biosynthetic process |
| 1625 | GO Biological Processes | GO:0044427 | chromosomal part |
| 1626 | GO Biological Processes | GO:0001667 | ameboidal-type cell migration |
| 1627 | GO Biological Processes | GO:0006886 | intracellular protein transport |
| 1628 | GO Biological Processes | GO:0006732 | coenzyme metabolic process |
| 1629 | GO Biological Processes | GO:0001937 | negative regulation of endothelial cell proliferation |
| 1630 | GO Biological Processes | GO:0033146 | regulation of intracellular estrogen receptor signaling pathway |
| 1631 | GO Biological Processes | GO:1903672 | positive regulation of sprouting angiogenesis |
| 1632 | GO Biological Processes | GO:0052548 | regulation of endopeptidase activity |
| 1633 | GO Biological Processes | GO:0044444 | cytoplasmic part |
| 1634 | GO Biological Processes | GO:0043232 | intracellular non-membrane-bounded organelle |
| 1635 | GO Biological Processes | GO:0043228 | non-membrane-bounded organelle |
| 1636 | GO Biological Processes | GO:0031965 | nuclear membrane |
| 1637 | GO Biological Processes | GO:0007520 | myoblast fusion |
| 1638 | GO Biological Processes | GO:0045646 | regulation of erythrocyte differentiation |
| 1639 | GO Biological Processes | GO:0045776 | negative regulation of blood pressure |
| 1640 | GO Biological Processes | GO:0046189 | phenol-containing compound biosynthetic process |
| 1641 | GO Biological Processes | GO:0051602 | response to electrical stimulus |
| 1642 | GO Biological Processes | GO:0060711 | labyrinthine layer development |
| 1643 | GO Biological Processes | GO:0070741 | response to interleukin-6 |
| 1644 | GO Biological Processes | GO:1901985 | positive regulation of protein acetylation |
| 1645 | GO Biological Processes | GO:0070888 | E-box binding |
| 1646 | GO Biological Processes | GO:0001774 | microglial cell activation |
| 1647 | GO Biological Processes | GO:0002251 | organ or tissue specific immune response |
| 1648 | GO Biological Processes | GO:0010799 | regulation of peptidyl-threonine phosphorylation |
| 1649 | GO Biological Processes | GO:0010828 | positive regulation of glucose transmembrane transport |
| 1650 | GO Biological Processes | GO:0022602 | ovulation cycle process |
| 1651 | GO Biological Processes | GO:0030850 | prostate gland development |
| 1652 | GO Biological Processes | GO:0038066 | p38MAPK cascade |
| 1653 | GO Biological Processes | GO:0060443 | mammary gland morphogenesis |
| 1654 | GO Biological Processes | GO:0061756 | leukocyte adhesion to vascular endothelial cell |
| 1655 | GO Biological Processes | GO:1903053 | regulation of extracellular matrix organization |
| 1656 | GO Biological Processes | GO:0030099 | myeloid cell differentiation |
| 1657 | GO Biological Processes | GO:0006111 | regulation of gluconeogenesis |
| 1658 | GO Biological Processes | GO:0045668 | negative regulation of osteoblast differentiation |
| 1659 | GO Biological Processes | GO:0045933 | positive regulation of muscle contraction |
| 1660 | GO Biological Processes | GO:0048806 | genitalia development |
| 1661 | GO Biological Processes | GO:0051898 | negative regulation of protein kinase B signaling |
| 1662 | GO Biological Processes | GO:0071364 | cellular response to epidermal growth factor stimulus |
| 1663 | GO Biological Processes | GO:0043900 | regulation of multi-organism process |
| 1664 | GO Biological Processes | GO:0030810 | positive regulation of nucleotide biosynthetic process |
| 1665 | GO Biological Processes | GO:0035329 | hippo signaling |
| 1666 | GO Biological Processes | GO:0042059 | negative regulation of epidermal growth factor receptor signaling pathway |
| 1667 | GO Biological Processes | GO:0042304 | regulation of fatty acid biosynthetic process |
| 1668 | GO Biological Processes | GO:0043124 | negative regulation of I-kappaB kinase/NF-kappaB signaling |
| 1669 | GO Biological Processes | GO:0045599 | negative regulation of fat cell differentiation |
| 1670 | GO Biological Processes | GO:0045843 | negative regulation of striated muscle tissue development |
| 1671 | GO Biological Processes | GO:0050999 | regulation of nitric-oxide synthase activity |
| 1672 | GO Biological Processes | GO:1900373 | positive regulation of purine nucleotide biosynthetic process |
| 1673 | GO Biological Processes | GO:1904036 | negative regulation of epithelial cell apoptotic process |
| 1674 | GO Biological Processes | GO:0003012 | muscle system process |
| 1675 | GO Biological Processes | GO:0035327 | transcriptionally active chromatin |
| 1676 | GO Biological Processes | GO:0001754 | eye photoreceptor cell differentiation |
| 1677 | GO Biological Processes | GO:0002269 | leukocyte activation involved in inflammatory response |
| 1678 | GO Biological Processes | GO:0009409 | response to cold |
| 1679 | GO Biological Processes | GO:0010677 | negative regulation of cellular carbohydrate metabolic process |
| 1680 | GO Biological Processes | GO:0048512 | circadian behavior |
| 1681 | GO Biological Processes | GO:0048635 | negative regulation of muscle organ development |
| 1682 | GO Biological Processes | GO:1902808 | positive regulation of cell cycle G1/S phase transition |
| 1683 | GO Biological Processes | GO:1903580 | positive regulation of ATP metabolic process |
| 1684 | GO Biological Processes | GO:0052547 | regulation of peptidase activity |
| 1685 | GO Biological Processes | GO:0007622 | rhythmic behavior |
| 1686 | GO Biological Processes | GO:0010718 | positive regulation of epithelial to mesenchymal transition |
| 1687 | GO Biological Processes | GO:0014075 | response to amine |
| 1688 | GO Biological Processes | GO:0031018 | endocrine pancreas development |
| 1689 | GO Biological Processes | GO:0042461 | photoreceptor cell development |
| 1690 | GO Biological Processes | GO:0048146 | positive regulation of fibroblast proliferation |
| 1691 | GO Biological Processes | GO:0070897 | transcription preinitiation complex assembly |
| 1692 | GO Biological Processes | GO:1901862 | negative regulation of muscle tissue development |
| 1693 | GO Biological Processes | GO:0034774 | secretory granule lumen |
| 1694 | GO Biological Processes | GO:0002639 | positive regulation of immunoglobulin production |
| 1695 | GO Biological Processes | GO:0006692 | prostanoid metabolic process |
| 1696 | GO Biological Processes | GO:0006693 | prostaglandin metabolic process |
| 1697 | GO Biological Processes | GO:0046394 | carboxylic acid biosynthetic process |
| 1698 | GO Biological Processes | GO:0016053 | organic acid biosynthetic process |
| 1699 | GO Biological Processes | GO:0004601 | peroxidase activity |
| 1700 | GO Biological Processes | GO:0023061 | signal release |
| 1701 | GO Biological Processes | GO:0035176 | social behavior |
| 1702 | GO Biological Processes | GO:0045058 | T cell selection |
| 1703 | GO Biological Processes | GO:0048662 | negative regulation of smooth muscle cell proliferation |
| 1704 | GO Biological Processes | GO:0051703 | intraspecies interaction between organisms |
| 1705 | GO Biological Processes | GO:0060688 | regulation of morphogenesis of a branching structure |
| 1706 | GO Biological Processes | GO:0000166 | nucleotide binding |
| 1707 | GO Biological Processes | GO:1901265 | nucleoside phosphate binding |
| 1708 | GO Biological Processes | GO:0001098 | basal transcription machinery binding |
| 1709 | GO Biological Processes | GO:0001099 | basal RNA polymerase II transcription machinery binding |
| 1710 | GO Biological Processes | GO:0043621 | protein self-association |
| 1711 | GO Biological Processes | GO:0002711 | positive regulation of T cell mediated immunity |
| 1712 | GO Biological Processes | GO:0007566 | embryo implantation |
| 1713 | GO Biological Processes | GO:0010596 | negative regulation of endothelial cell migration |
| 1714 | GO Biological Processes | GO:0045661 | regulation of myoblast differentiation |
| 1715 | GO Biological Processes | GO:1901185 | negative regulation of ERBB signaling pathway |
| 1716 | GO Biological Processes | GO:0016684 | oxidoreductase activity, acting on peroxide as acceptor |
| 1717 | GO Biological Processes | GO:0045088 | regulation of innate immune response |
| 1718 | GO Biological Processes | GO:0009435 | NAD biosynthetic process |
| 1719 | GO Biological Processes | GO:0043536 | positive regulation of blood vessel endothelial cell migration |
| 1720 | GO Biological Processes | GO:0045912 | negative regulation of carbohydrate metabolic process |
| 1721 | GO Biological Processes | GO:0097366 | response to bronchodilator |
| 1722 | GO Biological Processes | GO:0002429 | immune response-activating cell surface receptor signaling pathway |
| 1723 | GO Biological Processes | GO:0043227 | membrane-bounded organelle |
| 1724 | GO Biological Processes | GO:0060205 | cytoplasmic vesicle lumen |
| 1725 | GO Biological Processes | GO:0005640 | nuclear outer membrane |
| 1726 | GO Biological Processes | GO:0005694 | chromosome |
| 1727 | GO Biological Processes | GO:0002042 | cell migration involved in sprouting angiogenesis |
| 1728 | GO Biological Processes | GO:0010611 | regulation of cardiac muscle hypertrophy |
| 1729 | GO Biological Processes | GO:1903428 | positive regulation of reactive oxygen species biosynthetic process |
| 1730 | GO Biological Processes | GO:0016597 | amino acid binding |
| 1731 | GO Biological Processes | GO:0001541 | ovarian follicle development |
| 1732 | GO Biological Processes | GO:0002833 | positive regulation of response to biotic stimulus |
| 1733 | GO Biological Processes | GO:0006584 | catecholamine metabolic process |
| 1734 | GO Biological Processes | GO:0009712 | catechol-containing compound metabolic process |
| 1735 | GO Biological Processes | GO:0032613 | interleukin-10 production |
| 1736 | GO Biological Processes | GO:0032653 | regulation of interleukin-10 production |
| 1737 | GO Biological Processes | GO:0035065 | regulation of histone acetylation |
| 1738 | GO Biological Processes | GO:0008544 | epidermis development |
| 1739 | GO Biological Processes | GO:0097110 | scaffold protein binding |
| 1740 | GO Biological Processes | GO:0140096 | catalytic activity, acting on a protein |
| 1741 | GO Biological Processes | GO:0005829 | cytosol |
| 1742 | GO Biological Processes | GO:0051726 | regulation of cell cycle |
| 1743 | GO Biological Processes | GO:0007409 | axonogenesis |
| 1744 | GO Biological Processes | GO:0042743 | hydrogen peroxide metabolic process |
| 1745 | GO Biological Processes | GO:0046324 | regulation of glucose import |
| 1746 | GO Biological Processes | GO:0048260 | positive regulation of receptor-mediated endocytosis |
| 1747 | GO Biological Processes | GO:0048037 | cofactor binding |
| 1748 | GO Biological Processes | GO:0043312 | neutrophil degranulation |
| 1749 | GO Biological Processes | GO:0000768 | syncytium formation by plasma membrane fusion |
| 1750 | GO Biological Processes | GO:0006636 | unsaturated fatty acid biosynthetic process |
| 1751 | GO Biological Processes | GO:0010043 | response to zinc ion |
| 1752 | GO Biological Processes | GO:0014743 | regulation of muscle hypertrophy |
| 1753 | GO Biological Processes | GO:0043030 | regulation of macrophage activation |
| 1754 | GO Biological Processes | GO:0045981 | positive regulation of nucleotide metabolic process |
| 1755 | GO Biological Processes | GO:0046456 | icosanoid biosynthetic process |
| 1756 | GO Biological Processes | GO:0048255 | mRNA stabilization |
| 1757 | GO Biological Processes | GO:0071398 | cellular response to fatty acid |
| 1758 | GO Biological Processes | GO:0140253 | cell-cell fusion |
| 1759 | GO Biological Processes | GO:1900544 | positive regulation of purine nucleotide metabolic process |
| 1760 | GO Biological Processes | GO:0002283 | neutrophil activation involved in immune response |
| 1761 | GO Biological Processes | GO:0010676 | positive regulation of cellular carbohydrate metabolic process |
| 1762 | GO Biological Processes | GO:0010830 | regulation of myotube differentiation |
| 1763 | GO Biological Processes | GO:0032768 | regulation of monooxygenase activity |
| 1764 | GO Biological Processes | GO:0045428 | regulation of nitric oxide biosynthetic process |
| 1765 | GO Biological Processes | GO:1904705 | regulation of vascular smooth muscle cell proliferation |
| 1766 | GO Biological Processes | GO:1990874 | vascular smooth muscle cell proliferation |
| 1767 | GO Biological Processes | GO:0005768 | endosome |
| 1768 | GO Biological Processes | GO:0097367 | carbohydrate derivative binding |
| 1769 | GO Biological Processes | GO:0019637 | organophosphate metabolic process |
| 1770 | GO Biological Processes | GO:0006949 | syncytium formation |
| 1771 | GO Biological Processes | GO:0032615 | interleukin-12 production |
| 1772 | GO Biological Processes | GO:0032655 | regulation of interleukin-12 production |
| 1773 | GO Biological Processes | GO:0032731 | positive regulation of interleukin-1 beta production |
| 1774 | GO Biological Processes | GO:0006940 | regulation of smooth muscle contraction |
| 1775 | GO Biological Processes | GO:0032371 | regulation of sterol transport |
| 1776 | GO Biological Processes | GO:0032374 | regulation of cholesterol transport |
| 1777 | GO Biological Processes | GO:0051148 | negative regulation of muscle cell differentiation |
| 1778 | GO Biological Processes | GO:0051926 | negative regulation of calcium ion transport |
| 1779 | GO Biological Processes | GO:0015711 | organic anion transport |
| 1780 | GO Biological Processes | GO:0042119 | neutrophil activation |
| 1781 | GO Biological Processes | GO:0002768 | immune response-regulating cell surface receptor signaling pathway |
| 1782 | GO Biological Processes | GO:0030520 | intracellular estrogen receptor signaling pathway |
| 1783 | GO Biological Processes | GO:0050994 | regulation of lipid catabolic process |
| 1784 | GO Biological Processes | GO:0051155 | positive regulation of striated muscle cell differentiation |
| 1785 | GO Biological Processes | GO:2000378 | negative regulation of reactive oxygen species metabolic process |
| 1786 | GO Biological Processes | GO:0030514 | negative regulation of BMP signaling pathway |
| 1787 | GO Biological Processes | GO:0035924 | cellular response to vascular endothelial growth factor stimulus |
| 1788 | GO Biological Processes | GO:0043388 | positive regulation of DNA binding |
| 1789 | GO Biological Processes | GO:0045669 | positive regulation of osteoblast differentiation |
| 1790 | GO Biological Processes | GO:0036230 | granulocyte activation |
| 1791 | GO Biological Processes | GO:0072507 | divalent inorganic cation homeostasis |
| 1792 | GO Biological Processes | GO:0099503 | secretory vesicle |
| 1793 | GO Biological Processes | GO:0005790 | smooth endoplasmic reticulum |
| 1794 | GO Biological Processes | GO:0032839 | dendrite cytoplasm |
| 1795 | GO Biological Processes | GO:0001501 | skeletal system development |
| 1796 | GO Biological Processes | GO:0010256 | endomembrane system organization |
| 1797 | GO Biological Processes | GO:0032757 | positive regulation of interleukin-8 production |
| 1798 | GO Biological Processes | GO:1903078 | positive regulation of protein localization to plasma membrane |
| 1799 | GO Biological Processes | GO:0070405 | ammonium ion binding |
| 1800 | GO Biological Processes | GO:0090066 | regulation of anatomical structure size |
| 1801 | GO Biological Processes | GO:0016740 | transferase activity |
| 1802 | GO Biological Processes | GO:0002720 | positive regulation of cytokine production involved in immune response |
| 1803 | GO Biological Processes | GO:1902373 | negative regulation of mRNA catabolic process |
| 1804 | GO Biological Processes | GO:2000756 | regulation of peptidyl-lysine acetylation |
| 1805 | GO Biological Processes | GO:0042093 | T-helper cell differentiation |
| 1806 | GO Biological Processes | GO:0046530 | photoreceptor cell differentiation |
| 1807 | GO Biological Processes | GO:0050766 | positive regulation of phagocytosis |
| 1808 | GO Biological Processes | GO:0051705 | multi-organism behavior |
| 1809 | GO Biological Processes | GO:0061564 | axon development |
| 1810 | GO Biological Processes | GO:0043229 | intracellular organelle |
| 1811 | GO Biological Processes | GO:0005886 | plasma membrane |
| 1812 | GO Biological Processes | GO:0043489 | RNA stabilization |
| 1813 | GO Biological Processes | GO:0002294 | CD4-positive, alpha-beta T cell differentiation involved in immune response |
| 1814 | GO Biological Processes | GO:0002548 | monocyte chemotaxis |
| 1815 | GO Biological Processes | GO:0010660 | regulation of muscle cell apoptotic process |
| 1816 | GO Biological Processes | GO:0032922 | circadian regulation of gene expression |
| 1817 | GO Biological Processes | GO:0045824 | negative regulation of innate immune response |
| 1818 | GO Biological Processes | GO:0033293 | monocarboxylic acid binding |
| 1819 | GO Biological Processes | GO:0002287 | alpha-beta T cell activation involved in immune response |
| 1820 | GO Biological Processes | GO:0002293 | alpha-beta T cell differentiation involved in immune response |
| 1821 | GO Biological Processes | GO:0006360 | transcription by RNA polymerase I |
| 1822 | GO Biological Processes | GO:1903902 | positive regulation of viral life cycle |
| 1823 | GO Biological Processes | GO:0001618 | virus receptor activity |
| 1824 | GO Biological Processes | GO:0104005 | hijacked molecular function |
| 1825 | GO Biological Processes | GO:0010633 | negative regulation of epithelial cell migration |
| 1826 | GO Biological Processes | GO:0016239 | positive regulation of macroautophagy |
| 1827 | GO Biological Processes | GO:0032722 | positive regulation of chemokine production |
| 1828 | GO Biological Processes | GO:0042531 | positive regulation of tyrosine phosphorylation of STAT protein |
| 1829 | GO Biological Processes | GO:0071300 | cellular response to retinoic acid |
| 1830 | GO Biological Processes | GO:0043254 | regulation of protein complex assembly |
| 1831 | GO Biological Processes | GO:0043299 | leukocyte degranulation |
| 1832 | GO Biological Processes | GO:0032732 | positive regulation of interleukin-1 production |
| 1833 | GO Biological Processes | GO:1904377 | positive regulation of protein localization to cell periphery |
| 1834 | GO Biological Processes | GO:1901575 | organic substance catabolic process |
| 1835 | GO Biological Processes | GO:0050794 | regulation of cellular process |
| 1836 | GO Biological Processes | GO:0046323 | glucose import |
| 1837 | GO Biological Processes | GO:0071479 | cellular response to ionizing radiation |
| 1838 | GO Biological Processes | GO:0090398 | cellular senescence |
| 1839 | GO Biological Processes | GO:0045321 | leukocyte activation |
| 1840 | GO Biological Processes | GO:0006801 | superoxide metabolic process |
| 1841 | GO Biological Processes | GO:0006809 | nitric oxide biosynthetic process |
| 1842 | GO Biological Processes | GO:0010657 | muscle cell apoptotic process |
| 1843 | GO Biological Processes | GO:0034121 | regulation of toll-like receptor signaling pathway |
| 1844 | GO Biological Processes | GO:0050805 | negative regulation of synaptic transmission |
| 1845 | GO Biological Processes | GO:0002275 | myeloid cell activation involved in immune response |
| 1846 | GO Biological Processes | GO:0002637 | regulation of immunoglobulin production |
| 1847 | GO Biological Processes | GO:0016607 | nuclear speck |
| 1848 | GO Biological Processes | GO:0002292 | T cell differentiation involved in immune response |
| 1849 | GO Biological Processes | GO:0071944 | cell periphery |
| 1850 | GO Biological Processes | GO:0051493 | regulation of cytoskeleton organization |
| 1851 | GO Biological Processes | GO:0006635 | fatty acid beta-oxidation |
| 1852 | GO Biological Processes | GO:0010827 | regulation of glucose transmembrane transport |
| 1853 | GO Biological Processes | GO:0015909 | long-chain fatty acid transport |
| 1854 | GO Biological Processes | GO:0055013 | cardiac muscle cell development |
| 1855 | GO Biological Processes | GO:1903670 | regulation of sprouting angiogenesis |
| 1856 | GO Biological Processes | GO:0033613 | activating transcription factor binding |
| 1857 | GO Biological Processes | GO:0032890 | regulation of organic acid transport |
| 1858 | GO Biological Processes | GO:0045739 | positive regulation of DNA repair |
| 1859 | GO Biological Processes | GO:1902369 | negative regulation of RNA catabolic process |
| 1860 | GO Biological Processes | GO:0019674 | NAD metabolic process |
| 1861 | GO Biological Processes | GO:0030279 | negative regulation of ossification |
| 1862 | GO Biological Processes | GO:0031016 | pancreas development |
| 1863 | GO Biological Processes | GO:0046209 | nitric oxide metabolic process |
| 1864 | GO Biological Processes | GO:1901983 | regulation of protein acetylation |
| 1865 | GO Biological Processes | GO:0019748 | secondary metabolic process |
| 1866 | GO Biological Processes | GO:2001057 | reactive nitrogen species metabolic process |
| 1867 | GO Biological Processes | GO:0008013 | beta-catenin binding |
| 1868 | GO Biological Processes | GO:0016209 | antioxidant activity |
| 1869 | GO Biological Processes | GO:0043154 | negative regulation of cysteine-type endopeptidase activity involved in apoptotic process |
| 1870 | GO Biological Processes | GO:1903530 | regulation of secretion by cell |
| 1871 | GO Biological Processes | GO:0002709 | regulation of T cell mediated immunity |
| 1872 | GO Biological Processes | GO:0003300 | cardiac muscle hypertrophy |
| 1873 | GO Biological Processes | GO:0055006 | cardiac cell development |
| 1874 | GO Biological Processes | GO:0002312 | B cell activation involved in immune response |
| 1875 | GO Biological Processes | GO:0006110 | regulation of glycolytic process |
| 1876 | GO Biological Processes | GO:0022617 | extracellular matrix disassembly |
| 1877 | GO Biological Processes | GO:0042509 | regulation of tyrosine phosphorylation of STAT protein |
| 1878 | GO Biological Processes | GO:0045913 | positive regulation of carbohydrate metabolic process |
| 1879 | GO Biological Processes | GO:0051205 | protein insertion into membrane |
| 1880 | GO Biological Processes | GO:1901992 | positive regulation of mitotic cell cycle phase transition |
| 1881 | GO Biological Processes | GO:0030512 | negative regulation of transforming growth factor beta receptor signaling pathway |
| 1882 | GO Biological Processes | GO:0001892 | embryonic placenta development |
| 1883 | GO Biological Processes | GO:0007260 | tyrosine phosphorylation of STAT protein |
| 1884 | GO Biological Processes | GO:0014897 | striated muscle hypertrophy |
| 1885 | GO Biological Processes | GO:0030811 | regulation of nucleotide catabolic process |
| 1886 | GO Biological Processes | GO:0043502 | regulation of muscle adaptation |
| 1887 | GO Biological Processes | GO:1901224 | positive regulation of NIK/NF-kappaB signaling |
| 1888 | GO Biological Processes | GO:0001942 | hair follicle development |
| 1889 | GO Biological Processes | GO:0032637 | interleukin-8 production |
| 1890 | GO Biological Processes | GO:0032677 | regulation of interleukin-8 production |
| 1891 | GO Biological Processes | GO:0043367 | CD4-positive, alpha-beta T cell differentiation |
| 1892 | GO Biological Processes | GO:0045445 | myoblast differentiation |
| 1893 | GO Biological Processes | GO:0014896 | muscle hypertrophy |
| 1894 | GO Biological Processes | GO:1903845 | negative regulation of cellular response to transforming growth factor beta stimulus |
| 1895 | GO Biological Processes | GO:0048667 | cell morphogenesis involved in neuron differentiation |
| 1896 | GO Biological Processes | GO:0051213 | dioxygenase activity |
| 1897 | GO Biological Processes | GO:0006094 | gluconeogenesis |
| 1898 | GO Biological Processes | GO:0022404 | molting cycle process |
| 1899 | GO Biological Processes | GO:0022405 | hair cycle process |
| 1900 | GO Biological Processes | GO:2000117 | negative regulation of cysteine-type endopeptidase activity |
| 1901 | GO Biological Processes | GO:0098542 | defense response to other organism |
| 1902 | GO Biological Processes | GO:0050829 | defense response to Gram-negative bacterium |
| 1903 | GO Biological Processes | GO:0060333 | interferon-gamma-mediated signaling pathway |
| 1904 | GO Biological Processes | GO:0061912 | selective autophagy |
| 1905 | GO Biological Processes | GO:0098773 | skin epidermis development |
| 1906 | GO Biological Processes | GO:0042058 | regulation of epidermal growth factor receptor signaling pathway |
| 1907 | GO Biological Processes | GO:0060291 | long-term synaptic potentiation |
| 1908 | GO Biological Processes | GO:0009925 | basal plasma membrane |
| 1909 | GO Biological Processes | GO:0001776 | leukocyte homeostasis |
| 1910 | GO Biological Processes | GO:0019319 | hexose biosynthetic process |
| 1911 | GO Biological Processes | GO:0034644 | cellular response to UV |
| 1912 | GO Biological Processes | GO:0042475 | odontogenesis of dentin-containing tooth |
| 1913 | GO Biological Processes | GO:0045778 | positive regulation of ossification |
| 1914 | GO Biological Processes | GO:0006919 | activation of cysteine-type endopeptidase activity involved in apoptotic process |
| 1915 | GO Biological Processes | GO:0032611 | interleukin-1 beta production |
| 1916 | GO Biological Processes | GO:0032651 | regulation of interleukin-1 beta production |
| 1917 | GO Biological Processes | GO:0032755 | positive regulation of interleukin-6 production |
| 1918 | GO Biological Processes | GO:0043470 | regulation of carbohydrate catabolic process |
| 1919 | GO Biological Processes | GO:0046427 | positive regulation of JAK-STAT cascade |
| 1920 | GO Biological Processes | GO:0051149 | positive regulation of muscle cell differentiation |
| 1921 | GO Biological Processes | GO:0070301 | cellular response to hydrogen peroxide |
| 1922 | GO Biological Processes | GO:0097306 | cellular response to alcohol |
| 1923 | GO Biological Processes | GO:0044248 | cellular catabolic process |
| 1924 | GO Biological Processes | GO:0007589 | body fluid secretion |
| 1925 | GO Biological Processes | GO:0010717 | regulation of epithelial to mesenchymal transition |
| 1926 | GO Biological Processes | GO:0032642 | regulation of chemokine production |
| 1927 | GO Biological Processes | GO:0002367 | cytokine production involved in immune response |
| 1928 | GO Biological Processes | GO:0002718 | regulation of cytokine production involved in immune response |
| 1929 | GO Biological Processes | GO:0031058 | positive regulation of histone modification |
| 1930 | GO Biological Processes | GO:0032602 | chemokine production |
| 1931 | GO Biological Processes | GO:0034502 | protein localization to chromosome |
| 1932 | GO Biological Processes | GO:0048010 | vascular endothelial growth factor receptor signaling pathway |
| 1933 | GO Biological Processes | GO:1904894 | positive regulation of STAT cascade |
| 1934 | GO Biological Processes | GO:0009057 | macromolecule catabolic process |
| 1935 | GO Biological Processes | GO:0000079 | regulation of cyclin-dependent protein serine/threonine kinase activity |
| 1936 | GO Biological Processes | GO:1903312 | negative regulation of mRNA metabolic process |
| 1937 | GO Biological Processes | GO:0043255 | regulation of carbohydrate biosynthetic process |
| 1938 | GO Biological Processes | GO:0046364 | monosaccharide biosynthetic process |
| 1939 | GO Biological Processes | GO:0050764 | regulation of phagocytosis |
| 1940 | GO Biological Processes | GO:0097194 | execution phase of apoptosis |
| 1941 | GO Biological Processes | GO:1901184 | regulation of ERBB signaling pathway |
| 1942 | GO Biological Processes | GO:1901264 | carbohydrate derivative transport |
| 1943 | GO Biological Processes | GO:0051046 | regulation of secretion |
| 1944 | GO Biological Processes | GO:0008585 | female gonad development |
| 1945 | GO Biological Processes | GO:0030316 | osteoclast differentiation |
| 1946 | GO Biological Processes | GO:0051196 | regulation of coenzyme metabolic process |
| 1947 | GO Biological Processes | GO:2001243 | negative regulation of intrinsic apoptotic signaling pathway |
| 1948 | GO Biological Processes | GO:0032760 | positive regulation of tumor necrosis factor production |
| 1949 | GO Biological Processes | GO:0043473 | pigmentation |
| 1950 | GO Biological Processes | GO:2001169 | regulation of ATP biosynthetic process |
| 1951 | GO Biological Processes | GO:0004497 | monooxygenase activity |
| 1952 | GO Biological Processes | GO:0006820 | anion transport |
| 1953 | GO Biological Processes | GO:0007631 | feeding behavior |
| 1954 | GO Biological Processes | GO:0030301 | cholesterol transport |
| 1955 | GO Biological Processes | GO:0042116 | macrophage activation |
| 1956 | GO Biological Processes | GO:0043648 | dicarboxylic acid metabolic process |
| 1957 | GO Biological Processes | GO:0072527 | pyrimidine-containing compound metabolic process |
| 1958 | GO Biological Processes | GO:1904029 | regulation of cyclin-dependent protein kinase activity |
| 1959 | GO Biological Processes | GO:0005975 | carbohydrate metabolic process |
| 1960 | GO Biological Processes | GO:0002377 | immunoglobulin production |
| 1961 | GO Biological Processes | GO:0045069 | regulation of viral genome replication |
| 1962 | GO Biological Processes | GO:1901989 | positive regulation of cell cycle phase transition |
| 1963 | GO Biological Processes | GO:0055065 | metal ion homeostasis |
| 1964 | GO Biological Processes | GO:0042102 | positive regulation of T cell proliferation |
| 1965 | GO Biological Processes | GO:0045639 | positive regulation of myeloid cell differentiation |
| 1966 | GO Biological Processes | GO:0048709 | oligodendrocyte differentiation |
| 1967 | GO Biological Processes | GO:0050830 | defense response to Gram-positive bacterium |
| 1968 | GO Biological Processes | GO:0098869 | cellular oxidant detoxification |
| 1969 | GO Biological Processes | GO:1901570 | fatty acid derivative biosynthetic process |
| 1970 | GO Biological Processes | GO:0002456 | T cell mediated immunity |
| 1971 | GO Biological Processes | GO:0015837 | amine transport |
| 1972 | GO Biological Processes | GO:0030510 | regulation of BMP signaling pathway |
| 1973 | GO Biological Processes | GO:0046545 | development of primary female sexual characteristics |
| 1974 | GO Biological Processes | GO:1903557 | positive regulation of tumor necrosis factor superfamily cytokine production |
| 1975 | GO Biological Processes | GO:0002824 | positive regulation of adaptive immune response based on somatic recombination of immune receptors built from immunoglobulin superfamily domains |
| 1976 | GO Biological Processes | GO:0007200 | phospholipase C-activating G protein-coupled receptor signaling pathway |
| 1977 | GO Biological Processes | GO:0035710 | CD4-positive, alpha-beta T cell activation |
| 1978 | GO Biological Processes | GO:0006939 | smooth muscle contraction |
| 1979 | GO Biological Processes | GO:0051341 | regulation of oxidoreductase activity |
| 1980 | GO Biological Processes | GO:0010638 | positive regulation of organelle organization |
| 1981 | GO Biological Processes | GO:0031967 | organelle envelope |
| 1982 | GO Biological Processes | GO:0031975 | envelope |
| 1983 | GO Biological Processes | GO:0043195 | terminal bouton |
| 1984 | GO Biological Processes | GO:0090277 | positive regulation of peptide hormone secretion |
| 1985 | GO Biological Processes | GO:0008593 | regulation of Notch signaling pathway |
| 1986 | GO Biological Processes | GO:0010595 | positive regulation of endothelial cell migration |
| 1987 | GO Biological Processes | GO:0043500 | muscle adaptation |
| 1988 | GO Biological Processes | GO:0048640 | negative regulation of developmental growth |
| 1989 | GO Biological Processes | GO:1903076 | regulation of protein localization to plasma membrane |
| 1990 | GO Biological Processes | GO:0002708 | positive regulation of lymphocyte mediated immunity |
| 1991 | GO Biological Processes | GO:0007569 | cell aging |
| 1992 | GO Biological Processes | GO:0032612 | interleukin-1 production |
| 1993 | GO Biological Processes | GO:0032652 | regulation of interleukin-1 production |
| 1994 | GO Biological Processes | GO:0062014 | negative regulation of small molecule metabolic process |
| 1995 | GO Biological Processes | GO:1904659 | glucose transmembrane transport |
| 1996 | GO Biological Processes | GO:0002062 | chondrocyte differentiation |
| 1997 | GO Biological Processes | GO:0002821 | positive regulation of adaptive immune response |
| 1998 | GO Biological Processes | GO:0055007 | cardiac muscle cell differentiation |
| 1999 | GO Biological Processes | GO:0071887 | leukocyte apoptotic process |
| 2000 | GO Biological Processes | GO:0048259 | regulation of receptor-mediated endocytosis |
| 2001 | GO Biological Processes | GO:0006275 | regulation of DNA replication |
| 2002 | GO Biological Processes | GO:0002286 | T cell activation involved in immune response |
| 2003 | GO Biological Processes | GO:0008645 | hexose transmembrane transport |
| 2004 | GO Biological Processes | GO:0018958 | phenol-containing compound metabolic process |
| 2005 | GO Biological Processes | GO:0048524 | positive regulation of viral process |
| 2006 | GO Biological Processes | GO:2001022 | positive regulation of response to DNA damage stimulus |
| 2007 | GO Biological Processes | GO:0015918 | sterol transport |
| 2008 | GO Biological Processes | GO:0032526 | response to retinoic acid |
| 2009 | GO Biological Processes | GO:0046632 | alpha-beta T cell differentiation |
| 2010 | GO Biological Processes | GO:0015749 | monosaccharide transmembrane transport |
| 2011 | GO Biological Processes | GO:0016101 | diterpenoid metabolic process |
| 2012 | GO Biological Processes | GO:0009062 | fatty acid catabolic process |
| 2013 | GO Biological Processes | GO:0043025 | neuronal cell body |
| 2014 | GO Biological Processes | GO:0005637 | nuclear inner membrane |
| 2015 | GO Biological Processes | GO:0034219 | carbohydrate transmembrane transport |
| 2016 | GO Biological Processes | GO:1990748 | cellular detoxification |
| 2017 | GO Biological Processes | GO:0005771 | multivesicular body |
| 2018 | GO Biological Processes | GO:0002702 | positive regulation of production of molecular mediator of immune response |
| 2019 | GO Biological Processes | GO:0006096 | glycolytic process |
| 2020 | GO Biological Processes | GO:0014902 | myotube differentiation |
| 2021 | GO Biological Processes | GO:0030282 | bone mineralization |
| 2022 | GO Biological Processes | GO:0033143 | regulation of intracellular steroid hormone receptor signaling pathway |
| 2023 | GO Biological Processes | GO:0046660 | female sex differentiation |
| 2024 | GO Biological Processes | GO:1901222 | regulation of NIK/NF-kappaB signaling |
| 2025 | GO Biological Processes | GO:0015630 | microtubule cytoskeleton |
| 2026 | GO Biological Processes | GO:0006757 | ATP generation from ADP |
| 2027 | GO Biological Processes | GO:0018107 | peptidyl-threonine phosphorylation |
| 2028 | GO Biological Processes | GO:0044070 | regulation of anion transport |
| 2029 | GO Biological Processes | GO:0030218 | erythrocyte differentiation |
| 2030 | GO Biological Processes | GO:0045178 | basal part of cell |
| 2031 | GO Biological Processes | GO:0010906 | regulation of glucose metabolic process |
| 2032 | GO Biological Processes | GO:0016525 | negative regulation of angiogenesis |
| 2033 | GO Biological Processes | GO:0048593 | camera-type eye morphogenesis |
| 2034 | GO Biological Processes | GO:1900371 | regulation of purine nucleotide biosynthetic process |
| 2035 | GO Biological Processes | GO:0030808 | regulation of nucleotide biosynthetic process |
| 2036 | GO Biological Processes | GO:0042866 | pyruvate biosynthetic process |
| 2037 | GO Biological Processes | GO:0008610 | lipid biosynthetic process |
| 2038 | GO Biological Processes | GO:0043269 | regulation of ion transport |
| 2039 | GO Biological Processes | GO:0007173 | epidermal growth factor receptor signaling pathway |
| 2040 | GO Biological Processes | GO:0005856 | cytoskeleton |
| 2041 | GO Biological Processes | GO:0051193 | regulation of cofactor metabolic process |
| 2042 | GO Biological Processes | GO:2000181 | negative regulation of blood vessel morphogenesis |
| 2043 | GO Biological Processes | GO:0043226 | organelle |
| 2044 | GO Biological Processes | GO:0004714 | transmembrane receptor protein tyrosine kinase activity |
| 2045 | GO Biological Processes | GO:0031072 | heat shock protein binding |
| 2046 | GO Biological Processes | GO:0006721 | terpenoid metabolic process |
| 2047 | GO Biological Processes | GO:0021987 | cerebral cortex development |
| 2048 | GO Biological Processes | GO:0002040 | sprouting angiogenesis |
| 2049 | GO Biological Processes | GO:0017015 | regulation of transforming growth factor beta receptor signaling pathway |
| 2050 | GO Biological Processes | GO:1903578 | regulation of ATP metabolic process |
| 2051 | GO Biological Processes | GO:0030669 | clathrin-coated endocytic vesicle membrane |
| 2052 | GO Biological Processes | GO:0006911 | phagocytosis, engulfment |
| 2053 | GO Biological Processes | GO:0051101 | regulation of DNA binding |
| 2054 | GO Biological Processes | GO:0016491 | oxidoreductase activity |
| 2055 | GO Biological Processes | GO:0018210 | peptidyl-threonine modification |
| 2056 | GO Biological Processes | GO:0048565 | digestive tract development |
| 2057 | GO Biological Processes | GO:0019079 | viral genome replication |
| 2058 | GO Biological Processes | GO:0034101 | erythrocyte homeostasis |
| 2059 | GO Biological Processes | GO:0046031 | ADP metabolic process |
| 2060 | GO Biological Processes | GO:1903844 | regulation of cellular response to transforming growth factor beta stimulus |
| 2061 | GO Biological Processes | GO:1904375 | regulation of protein localization to cell periphery |
| 2062 | GO Biological Processes | GO:0043005 | neuron projection |
| 2063 | GO Biological Processes | GO:0030054 | cell junction |
| 2064 | GO Biological Processes | GO:0000904 | cell morphogenesis involved in differentiation |
| 2065 | GO Biological Processes | GO:0042476 | odontogenesis |
| 2066 | GO Biological Processes | GO:0042177 | negative regulation of protein catabolic process |
| 2067 | GO Biological Processes | GO:1903038 | negative regulation of leukocyte cell-cell adhesion |
| 2068 | GO Biological Processes | GO:1905477 | positive regulation of protein localization to membrane |
| 2069 | GO Biological Processes | GO:0006811 | ion transport |
| 2070 | GO Biological Processes | GO:0050680 | negative regulation of epithelial cell proliferation |
| 2071 | GO Biological Processes | GO:0050853 | B cell receptor signaling pathway |
| 2072 | GO Biological Processes | GO:0098754 | detoxification |
| 2073 | GO Biological Processes | GO:0006282 | regulation of DNA repair |
| 2074 | GO Biological Processes | GO:1905269 | positive regulation of chromatin organization |
| 2075 | GO Biological Processes | GO:0099024 | plasma membrane invagination |
| 2076 | GO Biological Processes | GO:1901343 | negative regulation of vasculature development |
| 2077 | GO Biological Processes | GO:0001936 | regulation of endothelial cell proliferation |
| 2078 | GO Biological Processes | GO:0006165 | nucleoside diphosphate phosphorylation |
| 2079 | GO Biological Processes | GO:0030534 | adult behavior |
| 2080 | GO Biological Processes | GO:0042770 | signal transduction in response to DNA damage |
| 2081 | GO Biological Processes | GO:0071482 | cellular response to light stimulus |
| 2082 | GO Biological Processes | GO:0044297 | cell body |
| 2083 | GO Biological Processes | GO:0098797 | plasma membrane protein complex |
| 2084 | GO Biological Processes | GO:0002705 | positive regulation of leukocyte mediated immunity |
| 2085 | GO Biological Processes | GO:0042157 | lipoprotein metabolic process |
| 2086 | GO Biological Processes | GO:0043280 | positive regulation of cysteine-type endopeptidase activity involved in apoptotic process |
| 2087 | GO Biological Processes | GO:0046683 | response to organophosphorus |
| 2088 | GO Biological Processes | GO:0055123 | digestive system development |
| 2089 | GO Biological Processes | GO:0010508 | positive regulation of autophagy |
| 2090 | GO Biological Processes | GO:0046939 | nucleotide phosphorylation |
| 2091 | GO Biological Processes | GO:0019838 | growth factor binding |
| 2092 | GO Biological Processes | GO:0070851 | growth factor receptor binding |
| 2093 | GO Biological Processes | GO:0004713 | protein tyrosine kinase activity |
| 2094 | GO Biological Processes | GO:0020037 | heme binding |
| 2095 | GO Biological Processes | GO:0019199 | transmembrane receptor protein kinase activity |
| 2096 | GO Biological Processes | GO:0050671 | positive regulation of lymphocyte proliferation |
| 2097 | GO Biological Processes | GO:0071333 | cellular response to glucose stimulus |
| 2098 | GO Biological Processes | GO:0072329 | monocarboxylic acid catabolic process |
| 2099 | GO Biological Processes | GO:0005178 | integrin binding |
| 2100 | GO Biological Processes | GO:0030855 | epithelial cell differentiation |
| 2101 | GO Biological Processes | GO:0044283 | small molecule biosynthetic process |
| 2102 | GO Biological Processes | GO:0032635 | interleukin-6 production |
| 2103 | GO Biological Processes | GO:0032675 | regulation of interleukin-6 production |
| 2104 | GO Biological Processes | GO:0032946 | positive regulation of mononuclear cell proliferation |
| 2105 | GO Biological Processes | GO:0042542 | response to hydrogen peroxide |
| 2106 | GO Biological Processes | GO:0046887 | positive regulation of hormone secretion |
| 2107 | GO Biological Processes | GO:0045055 | regulated exocytosis |
| 2108 | GO Biological Processes | GO:0009135 | purine nucleoside diphosphate metabolic process |
| 2109 | GO Biological Processes | GO:0009179 | purine ribonucleoside diphosphate metabolic process |
| 2110 | GO Biological Processes | GO:0050714 | positive regulation of protein secretion |
| 2111 | GO Biological Processes | GO:0071331 | cellular response to hexose stimulus |
| 2112 | GO Biological Processes | GO:0046425 | regulation of JAK-STAT cascade |
| 2113 | GO Biological Processes | GO:0071326 | cellular response to monosaccharide stimulus |
| 2114 | GO Biological Processes | GO:0010324 | membrane invagination |
| 2115 | GO Biological Processes | GO:0008584 | male gonad development |
| 2116 | GO Biological Processes | GO:0009185 | ribonucleoside diphosphate metabolic process |
| 2117 | GO Biological Processes | GO:0034754 | cellular hormone metabolic process |
| 2118 | GO Biological Processes | GO:0046718 | viral entry into host cell |
| 2119 | GO Biological Processes | GO:0071236 | cellular response to antibiotic |
| 2120 | GO Biological Processes | GO:0046546 | development of primary male sexual characteristics |
| 2121 | GO Biological Processes | GO:0007292 | female gamete generation |
| 2122 | GO Biological Processes | GO:1900542 | regulation of purine nucleotide metabolic process |
| 2123 | GO Biological Processes | GO:1904892 | regulation of STAT cascade |
| 2124 | GO Biological Processes | GO:0030183 | B cell differentiation |
| 2125 | GO Biological Processes | GO:0043086 | negative regulation of catalytic activity |
| 2126 | GO Biological Processes | GO:0046906 | tetrapyrrole binding |
| 2127 | GO Biological Processes | GO:1901657 | glycosyl compound metabolic process |
| 2128 | GO Biological Processes | GO:0005506 | iron ion binding |
| 2129 | GO Biological Processes | GO:0005515 | protein binding |
| 2130 | GO Biological Processes | GO:0010212 | response to ionizing radiation |
| 2131 | GO Biological Processes | GO:0010634 | positive regulation of epithelial cell migration |
| 2132 | GO Biological Processes | GO:0006140 | regulation of nucleotide metabolic process |
| 2133 | GO Biological Processes | GO:0010675 | regulation of cellular carbohydrate metabolic process |
| 2134 | GO Biological Processes | GO:0090263 | positive regulation of canonical Wnt signaling pathway |
| 2135 | GO Biological Processes | GO:0001837 | epithelial to mesenchymal transition |
| 2136 | GO Biological Processes | GO:0008643 | carbohydrate transport |
| 2137 | GO Biological Processes | GO:0055088 | lipid homeostasis |
| 2138 | GO Biological Processes | GO:0001935 | endothelial cell proliferation |
| 2139 | GO Biological Processes | GO:0043271 | negative regulation of ion transport |
| 2140 | GO Biological Processes | GO:0048754 | branching morphogenesis of an epithelial tube |
| 2141 | GO Biological Processes | GO:0050871 | positive regulation of B cell activation |
| 2142 | GO Biological Processes | GO:0071322 | cellular response to carbohydrate stimulus |
| 2143 | GO Biological Processes | GO:0000077 | DNA damage checkpoint |
| 2144 | GO Biological Processes | GO:0051592 | response to calcium ion |
| 2145 | GO Biological Processes | GO:2000045 | regulation of G1/S transition of mitotic cell cycle |
| 2146 | GO Biological Processes | GO:2000241 | regulation of reproductive process |
| 2147 | GO Biological Processes | GO:0045334 | clathrin-coated endocytic vesicle |
| 2148 | GO Biological Processes | GO:0035578 | azurophil granule lumen |
| 2149 | GO Biological Processes | GO:0030425 | dendrite |
| 2150 | GO Biological Processes | GO:0097447 | dendritic tree |
| 2151 | GO Biological Processes | GO:0120111 | neuron projection cytoplasm |
| 2152 | GO Biological Processes | GO:0031056 | regulation of histone modification |
| 2153 | GO Biological Processes | GO:2001056 | positive regulation of cysteine-type endopeptidase activity |
| 2154 | GO Biological Processes | GO:0002831 | regulation of response to biotic stimulus |
| 2155 | GO Biological Processes | GO:0006090 | pyruvate metabolic process |
| 2156 | GO Biological Processes | GO:0016999 | antibiotic metabolic process |
| 2157 | GO Biological Processes | GO:0045807 | positive regulation of endocytosis |
| 2158 | GO Biological Processes | GO:0008083 | growth factor activity |
| 2159 | GO Biological Processes | GO:0098772 | molecular function regulator |
| 2160 | GO Biological Processes | GO:0002224 | toll-like receptor signaling pathway |
| 2161 | GO Biological Processes | GO:0007605 | sensory perception of sound |
| 2162 | GO Biological Processes | GO:0002706 | regulation of lymphocyte mediated immunity |
| 2163 | GO Biological Processes | GO:0002822 | regulation of adaptive immune response based on somatic recombination of immune receptors built from immunoglobulin superfamily domains |
| 2164 | GO Biological Processes | GO:0030260 | entry into host cell |
| 2165 | GO Biological Processes | GO:0044409 | entry into host |
| 2166 | GO Biological Processes | GO:0046631 | alpha-beta T cell activation |
| 2167 | GO Biological Processes | GO:0048592 | eye morphogenesis |
| 2168 | GO Biological Processes | GO:0051806 | entry into cell of other organism involved in symbiotic interaction |
| 2169 | GO Biological Processes | GO:0055002 | striated muscle cell development |
| 2170 | GO Biological Processes | GO:0030162 | regulation of proteolysis |
| 2171 | GO Biological Processes | GO:0005737 | cytoplasm |
| 2172 | GO Biological Processes | GO:0016573 | histone acetylation |
| 2173 | GO Biological Processes | GO:0045931 | positive regulation of mitotic cell cycle |
| 2174 | GO Biological Processes | GO:0051828 | entry into other organism involved in symbiotic interaction |
| 2175 | GO Biological Processes | GO:0001678 | cellular glucose homeostasis |
| 2176 | GO Biological Processes | GO:0007519 | skeletal muscle tissue development |
| 2177 | GO Biological Processes | GO:0032640 | tumor necrosis factor production |
| 2178 | GO Biological Processes | GO:0032680 | regulation of tumor necrosis factor production |
| 2179 | GO Biological Processes | GO:0030424 | axon |
| 2180 | GO Biological Processes | GO:0032587 | ruffle membrane |
| 2181 | GO Biological Processes | GO:0031570 | DNA integrity checkpoint |
| 2182 | GO Biological Processes | GO:2001242 | regulation of intrinsic apoptotic signaling pathway |
| 2183 | GO Biological Processes | GO:0044463 | cell projection part |
| 2184 | GO Biological Processes | GO:0120038 | plasma membrane bounded cell projection part |
| 2185 | GO Biological Processes | GO:0009132 | nucleoside diphosphate metabolic process |
| 2186 | GO Biological Processes | GO:0018393 | internal peptidyl-lysine acetylation |
| 2187 | GO Biological Processes | GO:0002700 | regulation of production of molecular mediator of immune response |
| 2188 | GO Biological Processes | GO:0002793 | positive regulation of peptide secretion |
| 2189 | GO Biological Processes | GO:0006937 | regulation of muscle contraction |
| 2190 | GO Biological Processes | GO:0071706 | tumor necrosis factor superfamily cytokine production |
| 2191 | GO Biological Processes | GO:1903555 | regulation of tumor necrosis factor superfamily cytokine production |
| 2192 | GO Biological Processes | GO:0065003 | protein-containing complex assembly |
| 2193 | GO Biological Processes | GO:0006475 | internal protein amino acid acetylation |
| 2194 | GO Biological Processes | GO:0006633 | fatty acid biosynthetic process |
| 2195 | GO Biological Processes | GO:0006754 | ATP biosynthetic process |
| 2196 | GO Biological Processes | GO:0007259 | JAK-STAT cascade |
| 2197 | GO Biological Processes | GO:0030307 | positive regulation of cell growth |
| 2198 | GO Biological Processes | GO:0043433 | negative regulation of DNA-binding transcription factor activity |
| 2199 | GO Biological Processes | GO:0046661 | male sex differentiation |
| 2200 | GO Biological Processes | GO:1903900 | regulation of viral life cycle |
| 2201 | GO Biological Processes | GO:0003824 | catalytic activity |
| 2202 | GO Biological Processes | GO:0031982 | vesicle |
| 2203 | GO Biological Processes | GO:0042470 | melanosome |
| 2204 | GO Biological Processes | GO:0048770 | pigment granule |
| 2205 | GO Biological Processes | GO:0043467 | regulation of generation of precursor metabolites and energy |
| 2206 | GO Biological Processes | GO:0060538 | skeletal muscle organ development |
| 2207 | GO Biological Processes | GO:0006665 | sphingolipid metabolic process |
| 2208 | GO Biological Processes | GO:0097696 | STAT cascade |
| 2209 | GO Biological Processes | GO:0002819 | regulation of adaptive immune response |
| 2210 | GO Biological Processes | GO:0019953 | sexual reproduction |
| 2211 | GO Biological Processes | GO:0018394 | peptidyl-lysine acetylation |
| 2212 | GO Biological Processes | GO:0042129 | regulation of T cell proliferation |
| 2213 | GO Biological Processes | GO:0045766 | positive regulation of angiogenesis |
| 2214 | GO Biological Processes | GO:0048469 | cell maturation |
| 2215 | GO Biological Processes | GO:0055001 | muscle cell development |
| 2216 | GO Biological Processes | GO:0001659 | temperature homeostasis |
| 2217 | GO Biological Processes | GO:1902806 | regulation of cell cycle G1/S phase transition |
| 2218 | GO Biological Processes | GO:0009206 | purine ribonucleoside triphosphate biosynthetic process |
| 2219 | GO Biological Processes | GO:0009145 | purine nucleoside triphosphate biosynthetic process |
| 2220 | GO Biological Processes | GO:0038061 | NIK/NF-kappaB signaling |
| 2221 | GO Biological Processes | GO:0050954 | sensory perception of mechanical stimulus |
| 2222 | GO Biological Processes | GO:0030665 | clathrin-coated vesicle membrane |
| 2223 | GO Biological Processes | GO:0071346 | cellular response to interferon-gamma |
| 2224 | GO Biological Processes | GO:0021543 | pallium development |
| 2225 | GO Biological Processes | GO:0006887 | exocytosis |
| 2226 | GO Biological Processes | GO:0010950 | positive regulation of endopeptidase activity |
| 2227 | GO Biological Processes | GO:0016241 | regulation of macroautophagy |
| 2228 | GO Biological Processes | GO:0051099 | positive regulation of binding |
| 2229 | GO Biological Processes | GO:0009201 | ribonucleoside triphosphate biosynthetic process |
| 2230 | GO Biological Processes | GO:0051053 | negative regulation of DNA metabolic process |
| 2231 | GO Biological Processes | GO:0061138 | morphogenesis of a branching epithelium |
| 2232 | GO Biological Processes | GO:0030177 | positive regulation of Wnt signaling pathway |
| 2233 | GO Biological Processes | GO:0046328 | regulation of JNK cascade |
| 2234 | GO Biological Processes | GO:0006508 | proteolysis |
| 2235 | GO Biological Processes | GO:0090090 | negative regulation of canonical Wnt signaling pathway |
| 2236 | GO Biological Processes | GO:0022408 | negative regulation of cell-cell adhesion |
| 2237 | GO Biological Processes | GO:1905330 | regulation of morphogenesis of an epithelium |
| 2238 | GO Biological Processes | GO:0009168 | purine ribonucleoside monophosphate biosynthetic process |
| 2239 | GO Biological Processes | GO:0050796 | regulation of insulin secretion |
| 2240 | GO Biological Processes | GO:0009127 | purine nucleoside monophosphate biosynthetic process |
| 2241 | GO Biological Processes | GO:0007219 | Notch signaling pathway |
| 2242 | GO Biological Processes | GO:0002440 | production of molecular mediator of immune response |
| 2243 | GO Biological Processes | GO:0007626 | locomotory behavior |
| 2244 | GO Biological Processes | GO:0009166 | nucleotide catabolic process |
| 2245 | GO Biological Processes | GO:0099504 | synaptic vesicle cycle |
| 2246 | GO Biological Processes | GO:0002285 | lymphocyte activation involved in immune response |
| 2247 | GO Biological Processes | GO:0009142 | nucleoside triphosphate biosynthetic process |
| 2248 | GO Biological Processes | GO:0006338 | chromatin remodeling |
| 2249 | GO Biological Processes | GO:0050679 | positive regulation of epithelial cell proliferation |
| 2250 | GO Biological Processes | GO:1904018 | positive regulation of vasculature development |
| 2251 | GO Biological Processes | GO:0001763 | morphogenesis of a branching structure |
| 2252 | GO Biological Processes | GO:0043488 | regulation of mRNA stability |
| 2253 | GO Biological Processes | GO:0050731 | positive regulation of peptidyl-tyrosine phosphorylation |
| 2254 | GO Biological Processes | GO:0008022 | protein C-terminus binding |
| 2255 | GO Biological Processes | GO:0050864 | regulation of B cell activation |
| 2256 | GO Biological Processes | GO:0005516 | calmodulin binding |
| 2257 | GO Biological Processes | GO:0007179 | transforming growth factor beta receptor signaling pathway |
| 2258 | GO Biological Processes | GO:0010952 | positive regulation of peptidase activity |
| 2259 | GO Biological Processes | GO:0050852 | T cell receptor signaling pathway |
| 2260 | GO Biological Processes | GO:0034341 | response to interferon-gamma |
| 2261 | GO Biological Processes | GO:1901292 | nucleoside phosphate catabolic process |
| 2262 | GO Biological Processes | GO:0043679 | axon terminus |
| 2263 | GO Biological Processes | GO:0009156 | ribonucleoside monophosphate biosynthetic process |
| 2264 | GO Biological Processes | GO:0071456 | cellular response to hypoxia |
| 2265 | GO Biological Processes | GO:1901796 | regulation of signal transduction by p53 class mediator |
| 2266 | GO Biological Processes | GO:0009749 | response to glucose |
| 2267 | GO Biological Processes | GO:0042098 | T cell proliferation |
| 2268 | GO Biological Processes | GO:1905475 | regulation of protein localization to membrane |
| 2269 | GO Biological Processes | GO:0016052 | carbohydrate catabolic process |
| 2270 | GO Biological Processes | GO:0008092 | cytoskeletal protein binding |
| 2271 | GO Biological Processes | GO:0006473 | protein acetylation |
| 2272 | GO Biological Processes | GO:1903708 | positive regulation of hemopoiesis |
| 2273 | GO Biological Processes | GO:0051015 | actin filament binding |
| 2274 | GO Biological Processes | GO:0043487 | regulation of RNA stability |
| 2275 | GO Biological Processes | GO:0060348 | bone development |
| 2276 | GO Biological Processes | GO:0002703 | regulation of leukocyte mediated immunity |
| 2277 | GO Biological Processes | GO:1901215 | negative regulation of neuron death |
| 2278 | GO Biological Processes | GO:0002221 | pattern recognition receptor signaling pathway |
| 2279 | GO Biological Processes | GO:0009124 | nucleoside monophosphate biosynthetic process |
| 2280 | GO Biological Processes | GO:0030659 | cytoplasmic vesicle membrane |
| 2281 | GO Biological Processes | GO:0000075 | cell cycle checkpoint |
| 2282 | GO Biological Processes | GO:0006109 | regulation of carbohydrate metabolic process |
| 2283 | GO Biological Processes | GO:0036294 | cellular response to decreased oxygen levels |
| 2284 | GO Biological Processes | GO:0019722 | calcium-mediated signaling |
| 2285 | GO Biological Processes | GO:0071229 | cellular response to acid chemical |
| 2286 | GO Biological Processes | GO:0012506 | vesicle membrane |
| 2287 | GO Biological Processes | GO:0005815 | microtubule organizing center |
| 2288 | GO Biological Processes | GO:0016604 | nuclear body |
| 2289 | GO Biological Processes | GO:0044425 | membrane part |
| 2290 | GO Biological Processes | GO:0044424 | intracellular part |
| 2291 | GO Biological Processes | GO:0005622 | intracellular |
| 2292 | GO Biological Processes | GO:0044306 | neuron projection terminus |
| 2293 | GO Biological Processes | GO:0044430 | cytoskeletal part |
| 2294 | GO Biological Processes | GO:0007254 | JNK cascade |
| 2295 | GO Biological Processes | GO:0030178 | negative regulation of Wnt signaling pathway |
| 2296 | GO Biological Processes | GO:0007601 | visual perception |
| 2297 | GO Biological Processes | GO:0030073 | insulin secretion |
| 2298 | GO Biological Processes | GO:0061013 | regulation of mRNA catabolic process |
| 2299 | GO Biological Processes | GO:0097458 | neuron part |
| 2300 | GO Biological Processes | GO:0006643 | membrane lipid metabolic process |
| 2301 | GO Biological Processes | GO:0050870 | positive regulation of T cell activation |
| 2302 | GO Biological Processes | GO:0050953 | sensory perception of light stimulus |
| 2303 | GO Biological Processes | GO:0050807 | regulation of synapse organization |
| 2304 | GO Biological Processes | GO:0005766 | primary lysosome |
| 2305 | GO Biological Processes | GO:0042582 | azurophil granule |
| 2306 | GO Biological Processes | GO:0016051 | carbohydrate biosynthetic process |
| 2307 | GO Biological Processes | GO:1902275 | regulation of chromatin organization |
| 2308 | GO Biological Processes | GO:0030141 | secretory granule |
| 2309 | GO Biological Processes | GO:0061024 | membrane organization |
| 2310 | GO Biological Processes | GO:0002064 | epithelial cell development |
| 2311 | GO Biological Processes | GO:0097164 | ammonium ion metabolic process |
| 2312 | GO Biological Processes | GO:0008406 | gonad development |
| 2313 | GO Biological Processes | GO:0048588 | developmental cell growth |
| 2314 | GO Biological Processes | GO:0072686 | mitotic spindle |
| 2315 | GO Biological Processes | GO:0036477 | somatodendritic compartment |
| 2316 | GO Biological Processes | GO:0048705 | skeletal system morphogenesis |
| 2317 | GO Biological Processes | GO:0050670 | regulation of lymphocyte proliferation |
| 2318 | GO Biological Processes | GO:0050792 | regulation of viral process |
| 2319 | GO Biological Processes | GO:2001020 | regulation of response to DNA damage stimulus |
| 2320 | GO Biological Processes | GO:0005125 | cytokine activity |
| 2321 | GO Biological Processes | GO:0044087 | regulation of cellular component biogenesis |
| 2322 | GO Biological Processes | GO:0032869 | cellular response to insulin stimulus |
| 2323 | GO Biological Processes | GO:0032944 | regulation of mononuclear cell proliferation |
| 2324 | GO Biological Processes | GO:0002699 | positive regulation of immune effector process |
| 2325 | GO Biological Processes | GO:0050803 | regulation of synapse structure or activity |
| 2326 | GO Biological Processes | GO:0045137 | development of primary sexual characteristics |
| 2327 | GO Biological Processes | GO:2001234 | negative regulation of apoptotic signaling pathway |
| 2328 | GO Biological Processes | GO:0031901 | early endosome membrane |
| 2329 | GO Biological Processes | GO:0000922 | spindle pole |
| 2330 | GO Biological Processes | GO:0009898 | cytoplasmic side of plasma membrane |
| 2331 | GO Biological Processes | GO:0000082 | G1/S transition of mitotic cell cycle |
| 2332 | GO Biological Processes | GO:0030234 | enzyme regulator activity |
| 2333 | GO Biological Processes | GO:0008016 | regulation of heart contraction |
| 2334 | GO Biological Processes | GO:0005775 | vacuolar lumen |
| 2335 | GO Biological Processes | GO:0030662 | coated vesicle membrane |
| 2336 | GO Biological Processes | GO:0090575 | RNA polymerase II transcription factor complex |
| 2337 | GO Biological Processes | GO:0044242 | cellular lipid catabolic process |
| 2338 | GO Biological Processes | GO:0060560 | developmental growth involved in morphogenesis |
| 2339 | GO Biological Processes | GO:0031256 | leading edge membrane |
| 2340 | GO Biological Processes | GO:0001726 | ruffle |
| 2341 | GO Biological Processes | GO:0034404 | nucleobase-containing small molecule biosynthetic process |
| 2342 | GO Biological Processes | GO:0034764 | positive regulation of transmembrane transport |
| 2343 | GO Biological Processes | GO:1903039 | positive regulation of leukocyte cell-cell adhesion |
| 2344 | GO Biological Processes | GO:0045087 | innate immune response |
| 2345 | GO Biological Processes | GO:0010951 | negative regulation of endopeptidase activity |
| 2346 | GO Biological Processes | GO:0043903 | regulation of symbiosis, encompassing mutualism through parasitism |
| 2347 | GO Biological Processes | GO:1901605 | alpha-amino acid metabolic process |
| 2348 | GO Biological Processes | GO:1901617 | organic hydroxy compound biosynthetic process |
| 2349 | GO Biological Processes | GO:0043543 | protein acylation |
| 2350 | GO Biological Processes | GO:0043122 | regulation of I-kappaB kinase/NF-kappaB signaling |
| 2351 | GO Biological Processes | GO:0003007 | heart morphogenesis |
| 2352 | GO Biological Processes | GO:0030666 | endocytic vesicle membrane |
| 2353 | GO Biological Processes | GO:0010466 | negative regulation of peptidase activity |
| 2354 | GO Biological Processes | GO:0044798 | nuclear transcription factor complex |
| 2355 | GO Biological Processes | GO:0030136 | clathrin-coated vesicle |
| 2356 | GO Biological Processes | GO:0098562 | cytoplasmic side of membrane |
| 2357 | GO Biological Processes | GO:0044456 | synapse part |
| 2358 | GO Biological Processes | GO:2000027 | regulation of animal organ morphogenesis |
| 2359 | GO Biological Processes | GO:0042445 | hormone metabolic process |
| 2360 | GO Biological Processes | GO:0051924 | regulation of calcium ion transport |
| 2361 | GO Biological Processes | GO:0098802 | plasma membrane receptor complex |
| 2362 | GO Biological Processes | GO:0045637 | regulation of myeloid cell differentiation |
| 2363 | GO Biological Processes | GO:0044843 | cell cycle G1/S phase transition |
| 2364 | GO Biological Processes | GO:0030217 | T cell differentiation |
| 2365 | GO Biological Processes | GO:0051248 | negative regulation of protein metabolic process |
| 2366 | GO Biological Processes | GO:0021537 | telencephalon development |
| 2367 | GO Biological Processes | GO:0008021 | synaptic vesicle |
| 2368 | GO Biological Processes | GO:0043933 | protein-containing complex subunit organization |
| 2369 | GO Biological Processes | GO:0051607 | defense response to virus |
| 2370 | GO Biological Processes | GO:0032993 | protein-DNA complex |
| 2371 | GO Biological Processes | GO:0015850 | organic hydroxy compound transport |
| 2372 | GO Biological Processes | GO:0005126 | cytokine receptor binding |
| 2373 | GO Biological Processes | GO:0060047 | heart contraction |
| 2374 | GO Biological Processes | GO:0030336 | negative regulation of cell migration |
| 2375 | GO Biological Processes | GO:0046434 | organophosphate catabolic process |
| 2376 | GO Biological Processes | GO:0006836 | neurotransmitter transport |
| 2377 | GO Biological Processes | GO:0098742 | cell-cell adhesion via plasma-membrane adhesion molecules |
| 2378 | GO Biological Processes | GO:0003015 | heart process |
| 2379 | GO Biological Processes | GO:0021700 | developmental maturation |
| 2380 | GO Biological Processes | GO:0007162 | negative regulation of cell adhesion |
| 2381 | GO Biological Processes | GO:0007548 | sex differentiation |
| 2382 | GO Biological Processes | GO:0022409 | positive regulation of cell-cell adhesion |
| 2383 | GO Biological Processes | GO:0032838 | plasma membrane bounded cell projection cytoplasm |
| 2384 | GO Biological Processes | GO:1903532 | positive regulation of secretion by cell |
| 2385 | GO Biological Processes | GO:0007249 | I-kappaB kinase/NF-kappaB signaling |
| 2386 | GO Biological Processes | GO:0070382 | exocytic vesicle |
| 2387 | GO Biological Processes | GO:0031968 | organelle outer membrane |
| 2388 | GO Biological Processes | GO:0019867 | outer membrane |
| 2389 | GO Biological Processes | GO:0098588 | bounding membrane of organelle |
| 2390 | GO Biological Processes | GO:0016054 | organic acid catabolic process |
| 2391 | GO Biological Processes | GO:0030100 | regulation of endocytosis |
| 2392 | GO Biological Processes | GO:0046395 | carboxylic acid catabolic process |
| 2393 | GO Biological Processes | GO:0009152 | purine ribonucleotide biosynthetic process |
| 2394 | GO Biological Processes | GO:0072659 | protein localization to plasma membrane |
| 2395 | GO Biological Processes | GO:0050768 | negative regulation of neurogenesis |
| 2396 | GO Biological Processes | GO:2000146 | negative regulation of cell motility |
| 2397 | GO Biological Processes | GO:0072331 | signal transduction by p53 class mediator |
| 2398 | GO Biological Processes | GO:0097193 | intrinsic apoptotic signaling pathway |
| 2399 | GO Biological Processes | GO:0046651 | lymphocyte proliferation |
| 2400 | GO Biological Processes | GO:0050662 | coenzyme binding |
| 2401 | GO Biological Processes | GO:0031625 | ubiquitin protein ligase binding |
| 2402 | GO Biological Processes | GO:0016579 | protein deubiquitination |
| 2403 | GO Biological Processes | GO:0032409 | regulation of transporter activity |
| 2404 | GO Biological Processes | GO:0032943 | mononuclear cell proliferation |
| 2405 | GO Biological Processes | GO:0031334 | positive regulation of protein complex assembly |
| 2406 | GO Biological Processes | GO:0044262 | cellular carbohydrate metabolic process |
| 2407 | GO Biological Processes | GO:0042277 | peptide binding |
| 2408 | GO Biological Processes | GO:0090068 | positive regulation of cell cycle process |
| 2409 | GO Biological Processes | GO:0009260 | ribonucleotide biosynthetic process |
| 2410 | GO Biological Processes | GO:0051348 | negative regulation of transferase activity |
| 2411 | GO Biological Processes | GO:0065004 | protein-DNA complex assembly |
| 2412 | GO Biological Processes | GO:0031647 | regulation of protein stability |
| 2413 | GO Biological Processes | GO:0090305 | nucleic acid phosphodiester bond hydrolysis |
| 2414 | GO Biological Processes | GO:0016323 | basolateral plasma membrane |
| 2415 | GO Biological Processes | GO:0046390 | ribose phosphate biosynthetic process |
| 2416 | GO Biological Processes | GO:0044389 | ubiquitin-like protein ligase binding |
| 2417 | GO Biological Processes | GO:0006164 | purine nucleotide biosynthetic process |
| 2418 | GO Biological Processes | GO:0044877 | protein-containing complex binding |
| 2419 | GO Biological Processes | GO:0002758 | innate immune response-activating signal transduction |
| 2420 | GO Biological Processes | GO:0051961 | negative regulation of nervous system development |
| 2421 | GO Biological Processes | GO:0044265 | cellular macromolecule catabolic process |
| 2422 | GO Biological Processes | GO:0070646 | protein modification by small protein removal |
| 2423 | GO Biological Processes | GO:0045930 | negative regulation of mitotic cell cycle |
| 2424 | GO Biological Processes | GO:0005789 | endoplasmic reticulum membrane |
| 2425 | GO Biological Processes | GO:0098827 | endoplasmic reticulum subcompartment |
| 2426 | GO Biological Processes | GO:0050851 | antigen receptor-mediated signaling pathway |
| 2427 | GO Biological Processes | GO:0046034 | ATP metabolic process |
| 2428 | GO Biological Processes | GO:1901214 | regulation of neuron death |
| 2429 | GO Biological Processes | GO:0007204 | positive regulation of cytosolic calcium ion concentration |
| 2430 | GO Biological Processes | GO:0051047 | positive regulation of secretion |
| 2431 | GO Biological Processes | GO:0072522 | purine-containing compound biosynthetic process |
| 2432 | GO Biological Processes | GO:0042175 | nuclear outer membrane-endoplasmic reticulum membrane network |
| 2433 | GO Biological Processes | GO:0051271 | negative regulation of cellular component movement |
| 2434 | GO Biological Processes | GO:0045296 | cadherin binding |
| 2435 | GO Biological Processes | GO:0043010 | camera-type eye development |
| 2436 | GO Biological Processes | GO:0008202 | steroid metabolic process |
| 2437 | GO Biological Processes | GO:0004888 | transmembrane signaling receptor activity |
| 2438 | GO Biological Processes | GO:1901135 | carbohydrate derivative metabolic process |
| 2439 | GO Biological Processes | GO:0060562 | epithelial tube morphogenesis |
| 2440 | GO Biological Processes | GO:0044451 | nucleoplasm part |
| 2441 | GO Biological Processes | GO:0002218 | activation of innate immune response |
| 2442 | GO Biological Processes | GO:0071375 | cellular response to peptide hormone stimulus |
| 2443 | GO Biological Processes | GO:0006260 | DNA replication |
| 2444 | GO Biological Processes | GO:0040013 | negative regulation of locomotion |
| 2445 | GO Biological Processes | GO:0042113 | B cell activation |
| 2446 | GO Biological Processes | GO:0016236 | macroautophagy |
| 2447 | GO Biological Processes | GO:0050863 | regulation of T cell activation |
| 2448 | GO Biological Processes | GO:0001655 | urogenital system development |
| 2449 | GO Biological Processes | GO:1990778 | protein localization to cell periphery |
| 2450 | GO Biological Processes | GO:0042742 | defense response to bacterium |
| 2451 | GO Biological Processes | GO:1903311 | regulation of mRNA metabolic process |
| 2452 | GO Biological Processes | GO:0009205 | purine ribonucleoside triphosphate metabolic process |
| 2453 | GO Biological Processes | GO:0071824 | protein-DNA complex subunit organization |
| 2454 | GO Biological Processes | GO:0010506 | regulation of autophagy |
| 2455 | GO Biological Processes | GO:0016042 | lipid catabolic process |
| 2456 | GO Biological Processes | GO:0009144 | purine nucleoside triphosphate metabolic process |
| 2457 | GO Biological Processes | GO:0006936 | muscle contraction |
| 2458 | GO Biological Processes | GO:0051480 | regulation of cytosolic calcium ion concentration |
| 2459 | GO Biological Processes | GO:0007265 | Ras protein signal transduction |
| 2460 | GO Biological Processes | GO:0009199 | ribonucleoside triphosphate metabolic process |
| 2461 | GO Biological Processes | GO:0007059 | chromosome segregation |
| 2462 | GO Biological Processes | GO:0098796 | membrane protein complex |
| 2463 | GO Biological Processes | GO:0030135 | coated vesicle |
| 2464 | GO Biological Processes | GO:0009167 | purine ribonucleoside monophosphate metabolic process |
| 2465 | GO Biological Processes | GO:0045861 | negative regulation of proteolysis |
| 2466 | GO Biological Processes | GO:0070997 | neuron death |
| 2467 | GO Biological Processes | GO:0033218 | amide binding |
| 2468 | GO Biological Processes | GO:0009126 | purine nucleoside monophosphate metabolic process |
| 2469 | GO Biological Processes | GO:0002449 | lymphocyte mediated immunity |
| 2470 | GO Biological Processes | GO:0033043 | regulation of organelle organization |
| 2471 | GO Biological Processes | GO:0043687 | post-translational protein modification |
| 2472 | GO Biological Processes | GO:0051251 | positive regulation of lymphocyte activation |
| 2473 | GO Biological Processes | GO:0002460 | adaptive immune response based on somatic recombination of immune receptors built from immunoglobulin superfamily domains |
| 2474 | GO Biological Processes | GO:0150034 | distal axon |
| 2475 | GO Biological Processes | GO:0007186 | G protein-coupled receptor signaling pathway |
| 2476 | GO Biological Processes | GO:0009141 | nucleoside triphosphate metabolic process |
| 2477 | GO Biological Processes | GO:0009161 | ribonucleoside monophosphate metabolic process |
| 2478 | GO Biological Processes | GO:0001701 | in utero embryonic development |
| 2479 | GO Biological Processes | GO:0001654 | eye development |
| 2480 | GO Biological Processes | GO:0006520 | cellular amino acid metabolic process |
| 2481 | GO Biological Processes | GO:0045862 | positive regulation of proteolysis |
| 2482 | GO Biological Processes | GO:0150063 | visual system development |
| 2483 | GO Biological Processes | GO:0006959 | humoral immune response |
| 2484 | GO Biological Processes | GO:0030098 | lymphocyte differentiation |
| 2485 | GO Biological Processes | GO:0090150 | establishment of protein localization to membrane |
| 2486 | GO Biological Processes | GO:0006402 | mRNA catabolic process |
| 2487 | GO Biological Processes | GO:0048880 | sensory system development |
| 2488 | GO Biological Processes | GO:0009123 | nucleoside monophosphate metabolic process |
| 2489 | GO Biological Processes | GO:0045089 | positive regulation of innate immune response |
| 2490 | GO Biological Processes | GO:0030900 | forebrain development |
| 2491 | GO Biological Processes | GO:0045787 | positive regulation of cell cycle |
| 2492 | GO Biological Processes | GO:1901653 | cellular response to peptide |
| 2493 | GO Biological Processes | GO:2001233 | regulation of apoptotic signaling pathway |
| 2494 | GO Biological Processes | GO:0030139 | endocytic vesicle |
| 2495 | GO Biological Processes | GO:0014069 | postsynaptic density |
| 2496 | GO Biological Processes | GO:0002696 | positive regulation of leukocyte activation |
| 2497 | GO Biological Processes | GO:0018205 | peptidyl-lysine modification |
| 2498 | GO Biological Processes | GO:0032279 | asymmetric synapse |
| 2499 | GO Biological Processes | GO:0042176 | regulation of protein catabolic process |
| 2500 | GO Biological Processes | GO:0010959 | regulation of metal ion transport |
| 2501 | GO Biological Processes | GO:0007010 | cytoskeleton organization |
| 2502 | GO Biological Processes | GO:0030198 | extracellular matrix organization |
| 2503 | GO Biological Processes | GO:0098978 | glutamatergic synapse |
| 2504 | GO Biological Processes | GO:0050867 | positive regulation of cell activation |
| 2505 | GO Biological Processes | GO:0031253 | cell projection membrane |
| 2506 | GO Biological Processes | GO:0099572 | postsynaptic specialization |
| 2507 | GO Biological Processes | GO:0016324 | apical plasma membrane |
| 2508 | GO Biological Processes | GO:0005667 | transcription factor complex |
| 2509 | GO Biological Processes | GO:0098984 | neuron to neuron synapse |
| 2510 | GO Biological Processes | GO:1901990 | regulation of mitotic cell cycle phase transition |
| 2511 | GO Biological Processes | GO:0043588 | skin development |
| 2512 | GO Biological Processes | GO:0006401 | RNA catabolic process |
| 2513 | GO Biological Processes | GO:0006816 | calcium ion transport |
| 2514 | GO Biological Processes | GO:0050808 | synapse organization |
| 2515 | GO Biological Processes | GO:0006996 | organelle organization |
| 2516 | GO Biological Processes | GO:0045785 | positive regulation of cell adhesion |
| 2517 | GO Biological Processes | GO:0034470 | ncRNA processing |
| 2518 | GO Biological Processes | GO:0051051 | negative regulation of transport |
| 2519 | GO Biological Processes | GO:0003779 | actin binding |
| 2520 | GO Biological Processes | GO:0019932 | second-messenger-mediated signaling |
| 2521 | GO Biological Processes | GO:0006874 | cellular calcium ion homeostasis |
| 2522 | GO Biological Processes | GO:0051346 | negative regulation of hydrolase activity |
| 2523 | GO Biological Processes | GO:0042326 | negative regulation of phosphorylation |
| 2524 | GO Biological Processes | GO:0044282 | small molecule catabolic process |
| 2525 | GO Biological Processes | GO:0055074 | calcium ion homeostasis |
| 2526 | GO Biological Processes | GO:1901987 | regulation of cell cycle phase transition |
| 2527 | GO Biological Processes | GO:0033267 | axon part |
| 2528 | GO Biological Processes | GO:0016570 | histone modification |
| 2529 | GO Biological Processes | GO:0030133 | transport vesicle |
| 2530 | GO Biological Processes | GO:0043062 | extracellular structure organization |
| 2531 | GO Biological Processes | GO:0002697 | regulation of immune effector process |
| 2532 | GO Biological Processes | GO:0070838 | divalent metal ion transport |
| 2533 | GO Biological Processes | GO:0031252 | cell leading edge |
| 2534 | GO Biological Processes | GO:0072511 | divalent inorganic cation transport |
| 2535 | GO Biological Processes | GO:0045177 | apical part of cell |
| 2536 | GO Biological Processes | GO:0016569 | covalent chromatin modification |
| 2537 | GO Biological Processes | GO:0048018 | receptor ligand activity |
| 2538 | GO Biological Processes | GO:0072503 | cellular divalent inorganic cation homeostasis |
| 2539 | GO Biological Processes | GO:0042110 | T cell activation |
| 2540 | GO Biological Processes | GO:1903706 | regulation of hemopoiesis |
| 2541 | GO Biological Processes | GO:0050839 | cell adhesion molecule binding |
| 2542 | GO Biological Processes | GO:0005576 | extracellular region |
| 2543 | GO Biological Processes | GO:0051249 | regulation of lymphocyte activation |
| 2544 | GO Biological Processes | GO:0007264 | small GTPase mediated signal transduction |
| 2545 | GO Biological Processes | GO:0006091 | generation of precursor metabolites and energy |
| 2546 | GO Biological Processes | GO:0030545 | receptor regulator activity |
| 2547 | GO Biological Processes | GO:0005759 | mitochondrial matrix |
| 2548 | GO Biological Processes | GO:0005887 | integral component of plasma membrane |
| 2549 | GO Biological Processes | GO:0031090 | organelle membrane |
| 2550 | GO Biological Processes | GO:1901615 | organic hydroxy compound metabolic process |
| 2551 | GO Biological Processes | GO:0044772 | mitotic cell cycle phase transition |
| 2552 | GO Biological Processes | GO:0002009 | morphogenesis of an epithelium |
| 2553 | GO Biological Processes | GO:0006914 | autophagy |
| 2554 | GO Biological Processes | GO:0061919 | process utilizing autophagic mechanism |
| 2555 | GO Biological Processes | GO:0009150 | purine ribonucleotide metabolic process |
| 2556 | GO Biological Processes | GO:0022607 | cellular component assembly |
| 2557 | GO Biological Processes | GO:0098793 | presynapse |
| 2558 | GO Biological Processes | GO:0010008 | endosome membrane |
| 2559 | GO Biological Processes | GO:0044089 | positive regulation of cellular component biogenesis |
| 2560 | GO Biological Processes | GO:0031226 | intrinsic component of plasma membrane |
| 2561 | GO Biological Processes | GO:0022411 | cellular component disassembly |
| 2562 | GO Biological Processes | GO:0009259 | ribonucleotide metabolic process |
| 2563 | GO Biological Processes | GO:0045936 | negative regulation of phosphate metabolic process |
| 2564 | GO Biological Processes | GO:0010563 | negative regulation of phosphorus metabolic process |
| 2565 | GO Biological Processes | GO:0099568 | cytoplasmic region |
| 2566 | GO Biological Processes | GO:0006281 | DNA repair |
| 2567 | GO Biological Processes | GO:0006875 | cellular metal ion homeostasis |
| 2568 | GO Biological Processes | GO:0031984 | organelle subcompartment |
| 2569 | GO Biological Processes | GO:0034762 | regulation of transmembrane transport |
| 2570 | GO Biological Processes | GO:0006163 | purine nucleotide metabolic process |
| 2571 | GO Biological Processes | GO:0019866 | organelle inner membrane |
| 2572 | GO Biological Processes | GO:0019693 | ribose phosphate metabolic process |
| 2573 | GO Biological Processes | GO:0044440 | endosomal part |
| 2574 | GO Biological Processes | GO:0002694 | regulation of leukocyte activation |
| 2575 | GO Biological Processes | GO:0097190 | apoptotic signaling pathway |
| 2576 | GO Biological Processes | GO:0044770 | cell cycle phase transition |
| 2577 | GO Biological Processes | GO:0002250 | adaptive immune response |
| 2578 | GO Biological Processes | GO:0034660 | ncRNA metabolic process |
| 2579 | GO Biological Processes | GO:0043009 | chordate embryonic development |
| 2580 | GO Biological Processes | GO:0051301 | cell division |
| 2581 | GO Biological Processes | GO:0072521 | purine-containing compound metabolic process |
| 2582 | GO Biological Processes | GO:0007346 | regulation of mitotic cell cycle |
| 2583 | GO Biological Processes | GO:0044437 | vacuolar part |
| 2584 | GO Biological Processes | GO:0050865 | regulation of cell activation |
| 2585 | GO Biological Processes | GO:0098552 | side of membrane |
| 2586 | GO Biological Processes | GO:0009792 | embryo development ending in birth or egg hatching |
| 2587 | GO Biological Processes | GO:0048729 | tissue morphogenesis |
| 2588 | GO Biological Processes | GO:0042803 | protein homodimerization activity |
| 2589 | GO Biological Processes | GO:0098794 | postsynapse |
| 2590 | GO Biological Processes | GO:0044085 | cellular component biogenesis |
| 2591 | GO Biological Processes | GO:0016020 | membrane |
| 2592 | GO Biological Processes | GO:0072657 | protein localization to membrane |
| 2593 | GO Biological Processes | GO:0007276 | gamete generation |
| 2594 | GO Biological Processes | GO:0000323 | lytic vacuole |
| 2595 | GO Biological Processes | GO:0005764 | lysosome |
| 2596 | GO Biological Processes | GO:0000139 | Golgi membrane |
| 2597 | GO Biological Processes | GO:0005488 | binding |
| 2598 | GO Biological Processes | GO:0009987 | cellular process |
| 2599 | GO Biological Processes | GO:0007420 | brain development |
| 2600 | GO Biological Processes | GO:0046649 | lymphocyte activation |
| 2601 | GO Biological Processes | GO:1901137 | carbohydrate derivative biosynthetic process |
| 2602 | GO Biological Processes | GO:0010564 | regulation of cell cycle process |
| 2603 | GO Biological Processes | GO:0004930 | G protein-coupled receptor activity |
| 2604 | GO Biological Processes | GO:0005623 | cell |
| 2605 | GO Biological Processes | GO:0044464 | cell part |
| 2606 | GO Biological Processes | GO:0005773 | vacuole |
| 2607 | GO Biological Processes | GO:0098791 | Golgi subcompartment |
| 2608 | GO Biological Processes | GO:0016071 | mRNA metabolic process |
| 2609 | GO Biological Processes | GO:0009986 | cell surface |
| 2610 | GO Biological Processes | GO:0030001 | metal ion transport |
| 2611 | GO Biological Processes | GO:1903047 | mitotic cell cycle process |
| 2612 | GO Biological Processes | GO:0044431 | Golgi apparatus part |
| 2613 | GO Biological Processes | GO:0030163 | protein catabolic process |
| 2614 | GO Biological Processes | GO:0046983 | protein dimerization activity |
| 2615 | GO Biological Processes | GO:0032269 | negative regulation of cellular protein metabolic process |
| 2616 | GO Biological Processes | GO:0000278 | mitotic cell cycle |
| 2617 | GO Biological Processes | GO:0044429 | mitochondrial part |
| 2618 | GO Biological Processes | GO:0006812 | cation transport |
| 2619 | GO Biological Processes | GO:0070647 | protein modification by small protein conjugation or removal |
| 2620 | GO Biological Processes | GO:0034622 | cellular protein-containing complex assembly |
| 2621 | GO Biological Processes | GO:1901565 | organonitrogen compound catabolic process |
| 2622 | GO Biological Processes | GO:0022402 | cell cycle process |
| 2623 | GO Biological Processes | GO:0005615 | extracellular space |
| 2624 | GO Biological Processes | GO:0055085 | transmembrane transport |
| 2625 | GO Biological Processes | GO:0003723 | RNA binding |
| 2626 | GO Biological Processes | GO:0044421 | extracellular region part |
| 2627 | GO Biological Processes | GO:0016021 | integral component of membrane |
| 2628 | GO Biological Processes | GO:0031224 | intrinsic component of membrane |
| 2629 | GO Biological Processes | GO:0003674 | molecular_function |
| 2630 | GO Biological Processes | GO:0005575 | cellular_component |
| 2631 | GO Biological Processes | GO:0008150 | biological_process |

1. **Supplementary Table 5. 140 pathways were enriched by KEGG.**

| NO. | Category | KEGG | Description |
| --- | --- | --- | --- |
| 1 | KEGG Pathway | ko04933 | AGE-RAGE signaling pathway in diabetic complications |
| 2 | KEGG Pathway | ko04066 | HIF-1 signaling pathway |
| 3 | KEGG Pathway | ko05167 | Kaposi sarcoma-associated herpesvirus infection |
| 4 | KEGG Pathway | ko05205 | Proteoglycans in cancer |
| 5 | KEGG Pathway | ko05235 | PD-L1 expression and PD-1 checkpoint pathway in cancer |
| 6 | KEGG Pathway | ko05163 | Human cytomegalovirus infection |
| 7 | KEGG Pathway | ko05144 | Malaria |
| 8 | KEGG Pathway | ko05134 | Legionellosis |
| 9 | KEGG Pathway | ko05323 | Rheumatoid arthritis |
| 10 | KEGG Pathway | ko05164 | Influenza A |
| 11 | KEGG Pathway | ko05133 | Pertussis |
| 12 | KEGG Pathway | ko05212 | Pancreatic cancer |
| 13 | KEGG Pathway | ko01521 | EGFR tyrosine kinase inhibitor resistance |
| 14 | KEGG Pathway | ko04620 | Toll-like receptor signaling pathway |
| 15 | KEGG Pathway | ko05131 | Shigellosis |
| 16 | KEGG Pathway | ko05142 | Chagas disease |
| 17 | KEGG Pathway | ko05135 | Yersinia infection |
| 18 | KEGG Pathway | ko05200 | Pathways in cancer |
| 19 | KEGG Pathway | ko05219 | Bladder cancer |
| 20 | KEGG Pathway | ko05418 | Fluid shear stress and atherosclerosis |
| 21 | KEGG Pathway | ko05162 | Measles |
| 22 | KEGG Pathway | ko05161 | Hepatitis B |
| 23 | KEGG Pathway | ko05146 | Amoebiasis |
| 24 | KEGG Pathway | ko04064 | NF-kappa B signaling pathway |
| 25 | KEGG Pathway | ko05223 | Non-small cell lung cancer |
| 26 | KEGG Pathway | ko05321 | Inflammatory bowel disease |
| 27 | KEGG Pathway | ko05221 | Acute myeloid leukemia |
| 28 | KEGG Pathway | ko05230 | Central carbon metabolism in cancer |
| 29 | KEGG Pathway | ko05211 | Renal cell carcinoma |
| 30 | KEGG Pathway | ko05132 | Salmonella infection |
| 31 | KEGG Pathway | ko04015 | Rap1 signaling pathway |
| 32 | KEGG Pathway | ko05152 | Tuberculosis |
| 33 | KEGG Pathway | ko05231 | Choline metabolism in cancer |
| 34 | KEGG Pathway | ko04659 | Th17 cell differentiation |
| 35 | KEGG Pathway | ko05145 | Toxoplasmosis |
| 36 | KEGG Pathway | ko04668 | TNF signaling pathway |
| 37 | KEGG Pathway | ko04010 | MAPK signaling pathway |
| 38 | KEGG Pathway | ko04926 | Relaxin signaling pathway |
| 39 | KEGG Pathway | ko04068 | FoxO signaling pathway |
| 40 | KEGG Pathway | ko05140 | Leishmaniasis |
| 41 | KEGG Pathway | ko05160 | Hepatitis C |
| 42 | KEGG Pathway | ko04932 | Non-alcoholic fatty liver disease |
| 43 | KEGG Pathway | ko04217 | Necroptosis |
| 44 | KEGG Pathway | ko04630 | JAK-STAT signaling pathway |
| 45 | KEGG Pathway | ko05206 | MicroRNAs in cancer |
| 46 | KEGG Pathway | ko04621 | NOD-like receptor signaling pathway |
| 47 | KEGG Pathway | ko04151 | PI3K-Akt signaling pathway |
| 48 | KEGG Pathway | ko04062 | Chemokine signaling pathway |
| 49 | KEGG Pathway | ko04510 | Focal adhesion |
| 50 | KEGG Pathway | ko04072 | Phospholipase D signaling pathway |
| 51 | KEGG Pathway | ko04370 | VEGF signaling pathway |
| 52 | KEGG Pathway | ko05213 | Endometrial cancer |
| 53 | KEGG Pathway | ko04014 | Ras signaling pathway |
| 54 | KEGG Pathway | ko04920 | Adipocytokine signaling pathway |
| 55 | KEGG Pathway | ko05120 | Epithelial cell signaling in Helicobacter pylori infection |
| 56 | KEGG Pathway | ko05218 | Melanoma |
| 57 | KEGG Pathway | ko04917 | Prolactin signaling pathway |
| 58 | KEGG Pathway | ko05214 | Glioma |
| 59 | KEGG Pathway | ko05169 | Epstein-Barr virus infection |
| 60 | KEGG Pathway | ko05130 | Pathogenic Escherichia coli infection |
| 61 | KEGG Pathway | ko04012 | ErbB signaling pathway |
| 62 | KEGG Pathway | ko05210 | Colorectal cancer |
| 63 | KEGG Pathway | ko04657 | IL-17 signaling pathway |
| 64 | KEGG Pathway | ko05215 | Prostate cancer |
| 65 | KEGG Pathway | ko01522 | Endocrine resistance |
| 66 | KEGG Pathway | ko05165 | Human papillomavirus infection |
| 67 | KEGG Pathway | ko04625 | C-type lectin receptor signaling pathway |
| 68 | KEGG Pathway | ko05143 | African trypanosomiasis |
| 69 | KEGG Pathway | ko04931 | Insulin resistance |
| 70 | KEGG Pathway | ko04670 | Leukocyte transendothelial migration |
| 71 | KEGG Pathway | ko04935 | Growth hormone synthesis, secretion and action |
| 72 | KEGG Pathway | ko04919 | Thyroid hormone signaling pathway |
| 73 | KEGG Pathway | ko04380 | Osteoclast differentiation |
| 74 | KEGG Pathway | ko04915 | Estrogen signaling pathway |
| 75 | KEGG Pathway | ko04140 | Autophagy - animal |
| 76 | KEGG Pathway | ko04550 | Signaling pathways regulating pluripotency of stem cells |
| 77 | KEGG Pathway | ko04514 | Cell adhesion molecules |
| 78 | KEGG Pathway | ko05226 | Gastric cancer |
| 79 | KEGG Pathway | ko05224 | Breast cancer |
| 80 | KEGG Pathway | ko04218 | Cellular senescence |
| 81 | KEGG Pathway | ko05225 | Hepatocellular carcinoma |
| 82 | KEGG Pathway | ko04640 | Hematopoietic cell lineage |
| 83 | KEGG Pathway | ko05150 | Staphylococcus aureus infection |
| 84 | KEGG Pathway | ko04810 | Regulation of actin cytoskeleton |
| 85 | KEGG Pathway | ko05170 | Human immunodeficiency virus 1 infection |
| 86 | KEGG Pathway | ko04145 | Phagosome |
| 87 | KEGG Pathway | ko05166 | Human T-cell leukemia virus 1 infection |
| 88 | KEGG Pathway | ko01523 | Antifolate resistance |
| 89 | KEGG Pathway | ko05202 | Transcriptional misregulation in cancer |
| 90 | KEGG Pathway | ko05020 | Prion disease |
| 91 | KEGG Pathway | ko05332 | Graft-versus-host disease |
| 92 | KEGG Pathway | ko04060 | Cytokine-cytokine receptor interaction |
| 93 | KEGG Pathway | ko04940 | Type I diabetes mellitus |
| 94 | KEGG Pathway | ko04973 | Carbohydrate digestion and absorption |
| 95 | KEGG Pathway | ko05010 | Alzheimer disease |
| 96 | KEGG Pathway | ko04923 | Regulation of lipolysis in adipocytes |
| 97 | KEGG Pathway | ko04623 | Cytosolic DNA-sensing pathway |
| 98 | KEGG Pathway | ko04929 | GnRH secretion |
| 99 | KEGG Pathway | ko04137 | Mitophagy - animal |
| 100 | KEGG Pathway | ko04213 | Longevity regulating pathway - multiple species |
| 101 | KEGG Pathway | ko04622 | RIG-I-like receptor signaling pathway |
| 102 | KEGG Pathway | ko04520 | Adherens junction |
| 103 | KEGG Pathway | ko01524 | Platinum drug resistance |
| 104 | KEGG Pathway | ko05220 | Chronic myeloid leukemia |
| 105 | KEGG Pathway | ko04610 | Complement and coagulation cascades |
| 106 | KEGG Pathway | ko04540 | Gap junction |
| 107 | KEGG Pathway | ko04912 | GnRH signaling pathway |
| 108 | KEGG Pathway | ko05222 | Small cell lung cancer |
| 109 | KEGG Pathway | ko04211 | Longevity regulating pathway |
| 110 | KEGG Pathway | ko05017 | Spinocerebellar ataxia |
| 111 | KEGG Pathway | ko04061 | Viral protein interaction with cytokine and cytokine receptor |
| 112 | KEGG Pathway | ko04750 | Inflammatory mediator regulation of TRP channels |
| 113 | KEGG Pathway | ko04922 | Glucagon signaling pathway |
| 114 | KEGG Pathway | ko04928 | Parathyroid hormone synthesis, secretion and action |
| 115 | KEGG Pathway | ko04914 | Progesterone-mediated oocyte maturation |
| 116 | KEGG Pathway | ko04660 | T cell receptor signaling pathway |
| 117 | KEGG Pathway | ko04725 | Cholinergic synapse |
| 118 | KEGG Pathway | ko04071 | Sphingolipid signaling pathway |
| 119 | KEGG Pathway | ko04152 | AMPK signaling pathway |
| 120 | KEGG Pathway | ko04722 | Neurotrophin signaling pathway |
| 121 | KEGG Pathway | ko04611 | Platelet activation |
| 122 | KEGG Pathway | ko05168 | Herpes simplex virus 1 infection |
| 123 | KEGG Pathway | ko05416 | Viral myocarditis |
| 124 | KEGG Pathway | ko04728 | Dopaminergic synapse |
| 125 | KEGG Pathway | ko04371 | Apelin signaling pathway |
| 126 | KEGG Pathway | ko04664 | Fc epsilon RI signaling pathway |
| 127 | KEGG Pathway | ko04210 | Apoptosis |
| 128 | KEGG Pathway | ko04910 | Insulin signaling pathway |
| 129 | KEGG Pathway | ko04261 | Adrenergic signaling in cardiomyocytes |
| 130 | KEGG Pathway | ko04662 | B cell receptor signaling pathway |
| 131 | KEGG Pathway | ko04921 | Oxytocin signaling pathway |
| 132 | KEGG Pathway | ko04150 | mTOR signaling pathway |
| 133 | KEGG Pathway | ko04934 | Cushing syndrome |
| 134 | KEGG Pathway | ko04666 | Fc gamma R-mediated phagocytosis |
| 135 | KEGG Pathway | ko04022 | cGMP-PKG signaling pathway |
| 136 | KEGG Pathway | ko04650 | Natural killer cell mediated cytotoxicity |
| 137 | KEGG Pathway | ko05203 | Viral carcinogenesis |
| 138 | KEGG Pathway | ko04024 | cAMP signaling pathway |
| 139 | KEGG Pathway | ko04144 | Endocytosis |
| 140 | KEGG Pathway | ko04020 | Calcium signaling pathway |

1. **Supplementary Table 6. Effects of CCPI on cell viability.**

| NO. | Group | Cell viability (%) |
| --- | --- | --- |
| 1 | Control group | 100.00 |
| 2 | Control group | 98.89 |
| 3 | Control group | 95.20 |
| 4 | Control group | 98.28 |
| 5 | Control group | 100.00 |
| 6 | Control group | 96.21 |
| 7 | CCPI 0μg/mL group | 95.39 |
| 8 | CCPI 0μg/mL group | 100.00 |
| 9 | CCPI 0μg/mL group | 99.18 |
| 10 | CCPI 0μg/mL group | 99.10 |
| 11 | CCPI 0μg/mL group | 98.06 |
| 12 | CCPI 0μg/mL group | 100.00 |
| 13 | CCPI 15μg/mL group | 99.17 |
| 14 | CCPI 15μg/mL group | 100.00 |
| 15 | CCPI 15μg/mL group | 98.26 |
| 16 | CCPI 15μg/mL group | 95.36 |
| 17 | CCPI 15μg/mL group | 93.28 |
| 18 | CCPI 15μg/mL group | 91.44 |
| 19 | CCPI 30μg/mL group | 89.35 |
| 20 | CCPI 30μg/mL group | 100.00 |
| 21 | CCPI 30μg/mL group | 96.34 |
| 22 | CCPI 30μg/mL group | 92.13 |
| 23 | CCPI 30μg/mL group | 95.65 |
| 24 | CCPI 30μg/mL group | 97.84 |
| 25 | CCPI 60μg/mL group | 88.13 |
| 26 | CCPI 60μg/mL group | 95.27 |
| 27 | CCPI 60μg/mL group | 100.00 |
| 28 | CCPI 60μg/mL group | 96.22 |
| 29 | CCPI 60μg/mL group | 98.57 |
| 30 | CCPI 60μg/mL group | 93.33 |
| 31 | CCPI 120μg/mL group | 95.21 |
| 32 | CCPI 120μg/mL group | 85.65 |
| 33 | CCPI 120μg/mL group | 97.97 |
| 34 | CCPI 120μg/mL group | 90.39 |
| 35 | CCPI 120μg/mL group | 92.47 |
| 36 | CCPI 120μg/mL group | 89.13 |
| 37 | CCPI 240μg/mL group | 38.71 |
| 38 | CCPI 240μg/mL group | 23.47 |
| 39 | CCPI 240μg/mL group | 31.58 |
| 40 | CCPI 240μg/mL group | 45.23 |
| 41 | CCPI 240μg/mL group | 41.45 |
| 42 | CCPI 240μg/mL group | 52.17 |

1. **Supplementary Table 7. Effect of 120μg/mL CCPI on cell viability (%) in AD model at different time points.**

| NO. | Group | 4h | 8h | 12h | 24h | 48h |
| --- | --- | --- | --- | --- | --- | --- |
| 1 | Control group | 100.00 | 100.00 | 100.00 | 100.00 | 100.00 |
| 2 | Control group | 100.00 | 100.00 | 100.00 | 100.00 | 100.00 |
| 3 | Control group | 100.00 | 100.00 | 100.00 | 100.00 | 100.00 |
| 4 | Control group | 100.00 | 100.00 | 100.00 | 100.00 | 100.00 |
| 5 | Control group | 100.00 | 100.00 | 100.00 | 100.00 | 100.00 |
| 6 | Control group | 100.00 | 100.00 | 100.00 | 100.00 | 100.00 |
| 7 | AD model group | 56.89 | 54.59 | 56.65 | 57.00 | 48.16 |
| 8 | AD model group | 55.98 | 56.12 | 42.89 | 64.98 | 55.69 |
| 9 | AD model group | 49.70 | 50.29 | 46.88 | 58.37 | 50.24 |
| 10 | AD model group | 53.63 | 59.07 | 58.94 | 58.51 | 59.16 |
| 11 | AD model group | 52.35 | 59.88 | 54.44 | 49.71 | 45.45 |
| 12 | AD model group | 47.62 | 57.82 | 52.58 | 50.90 | 53.19 |
| 13 | 120μg/mL CCPI group | 47.95 | 73.62 | 85.09 | 87.74 | 79.32 |
| 14 | 120μg/mL CCPI group | 44.8 | 62.08 | 74.20 | 91.37 | 81.85 |
| 15 | 120μg/mL CCPI group | 51.15 | 66.12 | 77.84 | 98.87 | 92.61 |
| 16 | 120μg/mL CCPI group | 61.79 | 76.58 | 76.66 | 94.84 | 78.23 |
| 17 | 120μg/mL CCPI group | 66.20 | 65.94 | 69.01 | 94.20 | 79.44 |
| 18 | 120μg/mL CCPI group | 49.92 | 64.15 | 77.54 | 91.13 | 79.85 |

1. **Supplementary Table 8. Effect of CCPI on cell viability in AD model at different concentrations.**

| NO. | Group | Cell viability (%) |
| --- | --- | --- |
| 1 | Control group | 98.40 |
| 2 | Control group | 100.00 |
| 3 | Control group | 98.96 |
| 4 | Control group | 100.00 |
| 5 | Control group | 100.00 |
| 6 | Control group | 93.52 |
| 7 | AD model group | 50.17 |
| 8 | AD model group | 48.48 |
| 9 | AD model group | 51.54 |
| 10 | AD model group | 53.96 |
| 11 | AD model group | 61.38 |
| 12 | AD model group | 54.04 |
| 13 | 15μg/mL CCPI group | 62.22 |
| 14 | 15μg/mL CCPI group | 80.75 |
| 15 | 15μg/mL CCPI group | 64.85 |
| 16 | 15μg/mL CCPI group | 77.24 |
| 17 | 15μg/mL CCPI group | 79.98 |
| 18 | 15μg/mL CCPI group | 68.67 |
| 19 | 60μg/mL CCPI group | 80.95 |
| 20 | 60μg/mL CCPI group | 83.09 |
| 21 | 60μg/mL CCPI group | 78.24 |
| 22 | 60μg/mL CCPI group | 85.36 |
| 23 | 60μg/mL CCPI group | 90.16 |
| 24 | 60μg/mL CCPI group | 94.09 |
| 25 | 120μg/mL CCPI group | 91.29 |
| 26 | 120μg/mL CCPI group | 92.50 |
| 27 | 120μg/mL CCPI group | 97.28 |
| 28 | 120μg/mL CCPI group | 96.32 |
| 29 | 120μg/mL CCPI group | 96.21 |
| 30 | 120μg/mL CCPI group | 99.94 |

1. **Supplementary Table 9. Effect of CCPI on pro-inflammatory cytokines in the AD model detected by ELISA.**

| NO. | Group | IL-6 (pg·mL^-1^) | IL-1β (pg·mL^-1^) | TNF-α (pg·mL^-1^) |
| --- | --- | --- | --- | --- |
| 1 | Control group | 102.18 | 6.83 | 370.70 |
| 2 | Control group | 116.33 | 4.72 | 337.88 |
| 3 | Control group | 115.21 | 6.52 | 411.83 |
| 4 | Control group | 115.93 | 5.58 | 270.64 |
| 5 | Control group | 136.75 | 5.42 | 271.37 |
| 6 | Control group | 108.66 | 4.59 | 291.54 |
| 7 | AD model group | 531.34 | 11.10 | 1795.18 |
| 8 | AD model group | 581.36 | 15.78 | 2046.72 |
| 9 | AD model group | 615.10 | 16.09 | 1874.88 |
| 10 | AD model group | 529.18 | 9.43 | 1827.48 |
| 11 | AD model group | 681.79 | 13.55 | 2091.46 |
| 12 | AD model group | 586.85 | 16.19 | 1770.10 |
| 13 | 15μg/mL CCPI group | 550.68 | 10.03 | 1397.34 |
| 14 | 15μg/mL CCPI group | 510.92 | 11.73 | 1683.10 |
| 15 | 15μg/mL CCPI group | 505.08 | 6.58 | 1684.43 |
| 16 | 15μg/mL CCPI group | 474.67 | 13.70 | 1781.95 |
| 17 | 15μg/mL CCPI group | 443.38 | 8.14 | 1626.93 |
| 18 | 15μg/mL CCPI group | 467.50 | 11.76 | 1596.88 |
| 19 | 60μg/mL CCPI group | 341.35 | 9.73 | 1286.32 |
| 20 | 60μg/mL CCPI group | 381.41 | 10.13 | 1305.31 |
| 21 | 60μg/mL CCPI group | 318.91 | 8.07 | 1202.28 |
| 22 | 60μg/mL CCPI group | 291.44 | 9.72 | 1432.79 |
| 23 | 60μg/mL CCPI group | 354.21 | 7.39 | 1103.30 |
| 24 | 60μg/mL CCPI group | 352.94 | 8.69 | 1218.27 |
| 25 | 120μg/mL CCPI group | 117.47 | 6.04 | 545.86 |
| 26 | 120μg/mL CCPI group | 167.66 | 7.02 | 594.10 |
| 27 | 120μg/mL CCPI group | 179.04 | 6.59 | 737.54 |
| 28 | 120μg/mL CCPI group | 195.24 | 7.53 | 802.82 |
| 29 | 120μg/mL CCPI group | 192.62 | 6.21 | 619.98 |
| 30 | 120μg/mL CCPI group | 137.67 | 5.55 | 762.16 |

1. **Supplementary Table 10. Quantitative analysis of the M1-type marker (iNOS) and the M2-type marker (CD206).**

| NO. | Group | iNOS | CD206 | GAPDH |
| --- | --- | --- | --- | --- |
| 1 | Control group | 131.58 | 280.44 | 838.34 |
| 2 | Control group | 123.97 | 290.90 | 822.56 |
| 3 | Control group | 137.80 | 269.94 | 806.61 |
| 4 | AD model group | 816.08 | 75.03 | 885.17 |
| 5 | AD model group | 728.15 | 75.78 | 797.16 |
| 6 | AD model group | 849.06 | 82.72 | 812.96 |
| 7 | 15μg/mL CCPI group | 670.54 | 402.57 | 878.28 |
| 8 | 15μg/mL CCPI group | 658.86 | 409.87 | 745.98 |
| 9 | 15μg/mL CCPI group | 621.76 | 350.45 | 804.83 |
| 10 | 60μg/mL CCPI group | 525.15 | 493.79 | 881.80 |
| 11 | 60μg/mL CCPI group | 496.00 | 454.65 | 768.38 |
| 12 | 60μg/mL CCPI group | 567.78 | 443.41 | 881.17 |
| 13 | 120μg/mL CCPI group | 264.47 | 814.86 | 850.55 |
| 14 | 120μg/mL CCPI group | 227.76 | 769.26 | 842.11 |
| 15 | 120μg/mL CCPI group | 274.60 | 839.20 | 742.32 |

1. **Supplementary Table 16. Quantitative analysis of the IL-6/STAT3/VEGF signaling pathway.**

| NO. | Group | IL-6 | STAT3 | VEGF | GAPDH |
| --- | --- | --- | --- | --- | --- |
| 1 | Control group | 281.50 | 242.92 | 783.79 | 833.94 |
| 2 | Control group | 293.14 | 288.63 | 756.89 | 868.92 |
| 3 | Control group | 272.59 | 254.07 | 745.17 | 831.42 |
| 4 | AD model group | 892.76 | 884.35 | 150.17 | 832.82 |
| 5 | AD model group | 866.94 | 871.45 | 141.94 | 851.31 |
| 6 | AD model group | 875.15 | 825.79 | 153.56 | 866.55 |
| 7 | 15μg/mL CCPI group | 779.13 | 718.42 | 368.44 | 859.13 |
| 8 | 15μg/mL CCPI group | 748.36 | 694.66 | 356.54 | 841.21 |
| 9 | 15μg/mL CCPI group | 713.23 | 684.60 | 371.02 | 858.66 |
| 10 | 60μg/mL CCPI group | 556.57 | 569.54 | 399.91 | 858.69 |
| 11 | 60μg/mL CCPI group | 529.40 | 544.86 | 422.56 | 865.89 |
| 12 | 60μg/mL CCPI group | 495.71 | 525.32 | 445.19 | 877.16 |
| 13 | 120μg/mL CCPI group | 196.83 | 258.23 | 670.32 | 806.34 |
| 14 | 120μg/mL CCPI group | 208.40 | 261.51 | 693.40 | 857.22 |
| 15 | 120μg/mL CCPI group | 201.69 | 270.86 | 576.71 | 814.21 |

1. **Supplementary Table 12. Effects of LA on cell viability.**

| NO. | Group | Cell viability (%) |
| --- | --- | --- |
| 1 | Control group | 100.00 |
| 2 | Control group | 97.95 |
| 3 | Control group | 95.96 |
| 4 | Control group | 99.59 |
| 5 | Control group | 100.00 |
| 6 | Control group | 100.00 |
| 7 | NaOH group | 97.19 |
| 8 | NaOH group | 97.40 |
| 9 | NaOH group | 98.10 |
| 10 | NaOH group | 96.24 |
| 11 | NaOH group | 96.51 |
| 12 | NaOH group | 100.00 |
| 13 | LA 0μM group | 96.13 |
| 14 | LA 0μM group | 100.00 |
| 15 | LA 0μM group | 99.67 |
| 16 | LA 0μM group | 100.00 |
| 17 | LA 0μM group | 94.59 |
| 18 | LA 0μM group | 96.55 |
| 19 | LA 10μM group | 96.36 |
| 20 | LA 10μM group | 95.94 |
| 21 | LA 10μM group | 88.35 |
| 22 | LA 10μM group | 95.45 |
| 23 | LA 10μM group | 96.79 |
| 24 | LA 10μM group | 99.30 |
| 25 | LA 20μM group | 94.66 |
| 26 | LA 20μM group | 99.57 |
| 27 | LA 20μM group | 95.65 |
| 28 | LA 20μM group | 95.07 |
| 29 | LA 20μM group | 99.74 |
| 30 | LA 20μM group | 89.45 |
| 31 | LA 40μM group | 97.79 |
| 32 | LA 40μM group | 99.02 |
| 33 | LA 40μM group | 82.09 |
| 34 | LA 40μM group | 99.50 |
| 35 | LA 40μM group | 91.67 |
| 36 | LA 40μM group | 91.67 |
| 37 | LA 80μM group | 96.17 |
| 38 | LA 80μM group | 96.61 |
| 39 | LA 80μM group | 95.62 |
| 40 | LA 80μM group | 94.80 |
| 41 | LA 80μM group | 84.04 |
| 42 | LA 80μM group | 92.74 |
| 43 | LA 160μM group | 57.91 |
| 44 | LA 160μM group | 55.55 |
| 45 | LA 160μM group | 57.40 |
| 46 | LA 160μM group | 49.71 |
| 47 | LA 160μM group | 45.45 |
| 48 | LA 160μM group | 57.72 |

1. **Supplementary Table 13. Effect of LA on cell viability in AD model at different concentrations.**

| NO. | Group | Cell viability (%) |
| --- | --- | --- |
| 1 | Control group | 100.00 |
| 2 | Control group | 97.22 |
| 3 | Control group | 95.94 |
| 4 | Control group | 99.30 |
| 5 | Control group | 100.00 |
| 6 | Control group | 90.35 |
| 7 | AD model group | 60.17 |
| 8 | AD model group | 46.89 |
| 9 | AD model group | 55.29 |
| 10 | AD model group | 52.55 |
| 11 | AD model group | 44.37 |
| 12 | AD model group | 50.10 |
| 13 | LA 10μM group | 61.46 |
| 14 | LA 10μM group | 65.91 |
| 15 | LA 10μM group | 76.46 |
| 16 | LA 10μM group | 65.58 |
| 17 | LA 10μM group | 66.40 |
| 18 | LA 10μM group | 70.48 |
| 19 | LA 40μM group | 71.70 |
| 20 | LA 40μM group | 76.81 |
| 21 | LA 40μM group | 80.13 |
| 22 | LA 40μM group | 83.74 |
| 23 | LA 40μM group | 86.24 |
| 24 | LA 40μM group | 78.41 |
| 25 | LA 80μM group | 88.10 |
| 26 | LA 80μM group | 93.96 |
| 27 | LA 80μM group | 85.46 |
| 28 | LA 80μM group | 94.16 |
| 29 | LA 80μM group | 94.68 |
| 30 | LA 80μM group | 97.27 |

1. **Supplementary Table 14. Effect of LA on IL-6 levels in the AD model as determined by ELISA.**

| NO. | Group | IL-6 (pg·mL^-1^) |
| --- | --- | --- |
| 1 | Control group | 106.23 |
| 2 | Control group | 124.21 |
| 3 | Control group | 147.77 |
| 4 | Control group | 101.39 |
| 5 | Control group | 122.04 |
| 6 | Control group | 121.61 |
| 7 | AD model group | 468.53 |
| 8 | AD model group | 713.24 |
| 9 | AD model group | 726.16 |
| 10 | AD model group | 632.00 |
| 11 | AD model group | 372.41 |
| 12 | AD model group | 760.68 |
| 13 | LA 10μM group | 564.85 |
| 14 | LA 10μM group | 496.70 |
| 15 | LA 10μM group | 462.63 |
| 16 | LA 10μM group | 575.83 |
| 17 | LA 10μM group | 546.68 |
| 18 | LA 10μM group | 422.36 |
| 19 | LA 40μM group | 445.70 |
| 20 | LA 40μM group | 338.05 |
| 21 | LA 40μM group | 334.62 |
| 22 | LA 40μM group | 405.70 |
| 23 | LA 40μM group | 446.60 |
| 24 | LA 40μM group | 512.33 |
| 25 | LA 80μM group | 244.97 |
| 26 | LA 80μM group | 270.31 |
| 27 | LA 80μM group | 373.25 |
| 28 | LA 80μM group | 284.33 |
| 29 | LA 80μM group | 137.24 |
| 30 | LA 80μM group | 156.21 |

1. **Supplementary Table 15. Effects of CCPI and LA on IL-6 levels in AD model cells measured by ELISA.**

| NO. | Group | IL-6 (pg·mL^-1^) |
| --- | --- | --- |
| 1 | Control group | 130.03 |
| 2 | Control group | 145.16 |
| 3 | Control group | 113.49 |
| 4 | Control group | 140.71 |
| 5 | Control group | 100.61 |
| 6 | Control group | 123.66 |
| 7 | AD model group | 690.24 |
| 8 | AD model group | 607.71 |
| 9 | AD model group | 591.64 |
| 10 | AD model group | 519.83 |
| 11 | AD model group | 472.71 |
| 12 | AD model group | 495.14 |
| 13 | 120μg/mL CCPI group | 173.57 |
| 14 | 120μg/mL CCPI group | 148.32 |
| 15 | 120μg/mL CCPI group | 162.95 |
| 16 | 120μg/mL CCPI group | 176.33 |
| 17 | 120μg/mL CCPI group | 180.77 |
| 18 | 120μg/mL CCPI group | 171.40 |
| 19 | LA 80μM group | 235.87 |
| 20 | LA 80μM group | 267.23 |
| 21 | LA 80μM group | 237.67 |
| 22 | LA 80μM group | 213.33 |
| 23 | LA 80μM group | 227.32 |
| 24 | LA 80μM group | 226.03 |

1. **Supplementary Table 16. Quantitative analysis of the iNOS, CD206, and STAT3 phosphorylation proteins.**

| NO. | Group | iNOS | CD206 | GAPDH | p-STAT3 | STAT3 |
| --- | --- | --- | --- | --- | --- | --- |
| 1 | Control group | 167.76 | 731.95 | 976.43 | 127.73 | 561.31 |
| 2 | Control group | 201.07 | 781.35 | 898.26 | 110.54 | 554.42 |
| 3 | Control group | 175.86 | 746.51 | 983.94 | 103.46 | 611.64 |
| 4 | AD model group | 985.88 | 60.65 | 1002.72 | 1236.16 | 1074.66 |
| 5 | AD model group | 993.04 | 59.28 | 974.50 | 1311.74 | 1041.53 |
| 6 | AD model group | 1040.18 | 73.79 | 988.57 | 1259.72 | 1120.52 |
| 7 | 120μg/mL CCPI group | 326.01 | 910.75 | 1052.90 | 290.07 | 733.81 |
| 8 | 120μg/mL CCPI group | 377.65 | 985.47 | 999.33 | 301.89 | 768.86 |
| 9 | 120μg/mL CCPI group | 354.70 | 932.07 | 1095.02 | 325.35 | 814.56 |
| 10 | LA 80μM group | 427.49 | 729.25 | 971.25 | 381.90 | 748.66 |
| 11 | LA 80μM group | 403.70 | 766.75 | 1021.43 | 370.94 | 810.16 |
| 12 | LA 80μM group | 421.05 | 719.12 | 944.11 | 359.85 | 757.45 |
